# A randomised study of rituximab and belimumab sequential therapy in PR3 ANCA-associated vasculitis (COMBIVAS): design of the study protocol

**DOI:** 10.1186/s13063-023-07218-y

**Published:** 2023-03-11

**Authors:** Mark E. McClure, Seerapani Gopaluni, James Wason, Robert B. Henderson, Andre Van Maurik, Caroline C.O. Savage, Charles D. Pusey, Alan D. Salama, Paul A. Lyons, Jacinta Lee, Kim Mynard, David R. Jayne, Rachel B. Jones, on behalf the COMBIVAS investigators

**Affiliations:** 1 Vasculitis and Lupus Clinic, Cambridge University Hospitals, Cambridge, UK; 2 Department of Medicine, University of Cambridge, Cambridge, UK; 3 Population Health Sciences Institute, Newcastle University, Newcastle, UK; 4 GlaxoSmithKline, Stevenage, UK; 5 Department of Immunology and Inflammation, Imperial College London, London, UK; 6 UCL Centre for Nephrology, Royal Free Hospital, London, UK; 7 Cambridge Institute of Therapeutic Immunology and Infectious Disease, Jeffrey Cheah Biomedical Centre, Cambridge Biomedical Campus, Cambridge, UK

**Keywords:** ANCA, Vasculitis, Rituximab, Belimumab

## Abstract

**Background:**

Sequential B cell-targeted immunotherapy with BAFF antagonism (belimumab) and B cell depletion (rituximab) may enhance B cell targeting in ANCA-associated vasculitis (AAV) through several mechanisms.

**Methods:**

Study design: COMBIVAS is a randomised, double-blind, placebo-controlled trial designed to assess the mechanistic effects of sequential therapy of belimumab and rituximab in patients with active PR3 AAV. The recruitment target is 30 patients who meet the criteria for inclusion in the per-protocol analysis. Thirty-six participants have been randomised to one of the two treatment groups in a 1:1 ratio: either rituximab plus belimumab or rituximab plus placebo (both groups with the same tapering corticosteroid regimen), and recruitment is now closed (final patient enrolled April 2021). For each patient, the trial will last for 2 years comprising a 12-month treatment period followed by a 12-month follow-up period.

Participants: Participants have been recruited from five of seven UK trial sites. Eligibility criteria were age ≥ 18 years and a diagnosis of AAV with active disease (newly diagnosed or relapsing disease), along with a concurrent positive test for PR3 ANCA by ELISA.

Interventions: Rituximab 1000 mg was administered by intravenous infusions on day 8 and day 22. Weekly subcutaneous injections of 200 mg belimumab or placebo were initiated a week before rituximab on day 1 and then weekly through to week 51. All participants received a relatively low prednisolone (20 mg/day) starting dose from day 1 followed by a protocol-specified corticosteroid taper aiming for complete cessation by 3 months.

Outcomes: The primary endpoint of this study is time to PR3 ANCA negativity. Key secondary outcomes include change from baseline in naïve, transitional, memory, plasmablast B cell subsets (by flow cytometry) in the blood at months 3, 12, 18 and 24; time to clinical remission; time to relapse; and incidence of serious adverse events. Exploratory biomarker assessments include assessment of B cell receptor clonality, B cell and T cell functional assays, whole blood transcriptomic analysis and urinary lymphocyte and proteomic analysis. Inguinal lymph node and nasal mucosal biopsies have been performed on a subgroup of patients at baseline and month 3.

**Discussion:**

This experimental medicine study provides a unique opportunity to gain detailed insights into the immunological mechanisms of belimumab-rituximab sequential therapy across multiple body compartments in the setting of AAV.

**Trial registration:**

ClinicalTrials.gov NCT03967925. Registered on May 30, 2019.

**Supplementary Information:**

The online version contains supplementary material available at 10.1186/s13063-023-07218-y.

## Administrative information

**Table:** 

Title	A randomised study of rituximab and belimumab sequential therapy in PR3 ANCA-associated vasculitis (COMBIVAS): design of the study protocol
Trial registration	ClinicalTrials.gov Identifier: NCT03967925, May 30, 2019
Protocol version	5.0
Funding	This study is funded by the MRC GSK EMINENT programme
Author details	1 Vasculitis and Lupus Clinic, Cambridge University Hospitals, Cambridge, UK 2 Department of Medicine, University of Cambridge, Cambridge, UK 3 Population Health Sciences Institute, Newcastle University, Newcastle, UK 4 GlaxoSmithKline, Stevenage, UK 5 Department of Immunology and Inflammation, Imperial College London, London, UK 6 UCL Centre for Nephrology, Royal Free Hospital, London, UK 7 Cambridge Institute of Therapeutic Immunology and Infectious Disease, Jeffrey Cheah Biomedical Centre, Cambridge Biomedical Campus, Cambridge, UK * at the time of the study ^Ψ^ contributed equally
Name and contact information for trial sponsor	Joint sponsorship by Cambridge University Hospitals NHS Foundation Trust and the University of Cambridge Dr. Stephen Kelleher, Research and Development Director, Cambridge University Hospitals NHS Foundation Trust. add-tr.cctu@nhs.net Professor Ken Smith, Head of Department of Medicine, University of Cambridge. hodmed@medschl.cam.ac.uk
Role of sponsor	The Cambridge Clinical Trials Unit (CCTU) provides regulatory oversight of the study on behalf of the sponsors. The CCTU roles include pharmacovigilance, monitoring, clinical database build and management and facilitating set-up with oversight of protocol development and ethics and regulatory submission

## Introduction

### Background and rationale

ANCA-associated vasculitis (AAV) encompasses a group of multisystem autoimmune diseases characterised by necrotising small-vessel vasculitis and the presence of circulating autoantibodies (ANCAs) directed against the neutrophil cytoplasmic antigens proteinase 3 (PR3) and myeloperoxidase (MPO). B cell depletion with rituximab in combination with corticosteroids is associated with a reduction in vasculitis activity and ANCA levels in the majority of patients [1]. However, those with the PR3 ANCA subtype and predominantly granulomatous disease have a slower time to remission after rituximab and glucocorticoids, with a high subsequent relapse risk of 44% by 13 months [2, 3]. There is a need for newer therapies to reduce the time to remission, to spare glucocorticoid use and to promote long-lasting relapse-free remission.

The success of rituximab in AAV has underscored the importance of B cells in its pathogenesis and has paved the way for other B cell-targeted therapies. Elevated levels of circulating BAFF, a B cell survival cytokine, are observed in patients with AAV [4, 5] and may promote the positive selection of autoreactive B cells [6]. Belimumab binds soluble BAFF with high affinity and inhibits its biological activity, resulting in apoptotic B cell death and reduction in circulating B cell numbers. BAFF antagonism with belimumab has proven to be efficacious in systemic lupus erythematosus (SLE), including lupus nephritis [7, 8], and is undergoing evaluation in other autoimmune diseases. To date, the experience of belimumab use in AAV is limited to the Belimumab in Remission of Vasculitis (BREVAS) trial [9], a study comparing belimumab versus placebo (plus azathioprine and low-dose glucocorticoids in both groups) as maintenance therapy following a standard induction regimen with cyclophosphamide or rituximab. Changes in treatment practice led to the truncation of the study population after initiation, resulting in reduced sample size and power for some analyses. No difference in relapse rate (the primary efficacy endpoint) was observed between treatment arms in the overall patient population; however, a positive trend of improved efficacy (and higher infections) was seen in the subgroup of patients who received rituximab induction therapy followed by belimumab compared to placebo.

Sequential therapy with belimumab (BAFF antagonism) and rituximab (CD20 + B cell depletion) may enhance B cell targeting in AAV through several distinct but complementary mechanisms: (1) as belimumab targets both CD20 + and CD20 − plasmablast populations, but appears to spare CD27 + memory cells [10], and rituximab effectively depletes CD20 + cells (including circulating CD27 + memory cells) but has no effect on CD20 − cells, sequential therapy may impact a broader B cell population than targeting either BAFF or CD20 alone; (2) as high BAFF levels in tissue niches may retain B cells and protect against tissue B cell depletion by rituximab, sequential therapy may enhance B cell depletion at the tissue level; (3) in patients with SLE, belimumab therapy was associated with an early rise in peripheral blood memory B cells, possibly due to mobilisation from lymphoid tissue [11]. Once in the circulation, these memory B cells may be more amenable to rituximab-induced depletion; and (4) high BAFF levels during B cell reconstitution post rituximab promotes the return of autoreactive B cell resulting in more severe flares [12]. Therefore, regulation of BAFF levels post-depletion may allow greater stringency for B cell reconstitution, preventing re-emergence of autoreactive B cells and would directly target the effect of the rebound rise in BAFF levels seen after rituximab.

### Objectives

#### Primary: efficacy

- To compare the efficacy of belimumab (versus placebo) in combination with a single cycle of rituximab and corticosteroids in achieving PR3 ANCA negativity in participants with active AAV

#### Secondary: efficacy

- To assess the changes in PR3 ANCA following belimumab (or placebo) in combination with a single cycle of rituximab and corticosteroids in patients with active AAV

#### Secondary: pharmacodynamic

- To assess the changes in key leukocyte populations and B and T cell subsets in the blood during B cell depletion and B cell reconstitution

#### Secondary: efficacy (clinical)

- To compare the clinical efficacy of belimumab (versus placebo) in combination with a single cycle of rituximab and corticosteroids in participants with active AAV

#### Secondary: safety

- To compare the safety and tolerability of belimumab (versus placebo) in combination with a single cycle of rituximab and corticosteroids in participants with active AAV

#### Exploratory

- To assess the changes in key leukocyte populations and B and T cell subsets in lymph node and nasal tissue during B cell depletion using flow cytometry
- To assess the mechanistic changes in lymph node and nasal tissue during B cell depletion
- To assess the clonality of the reconstituted repertoire of B cells to investigate the differences in central and peripheral selection between individuals under different treatment regimens
- To assess the biomarker response and mechanism of action of belimumab combination with rituximab
- To compare the effect on patient-reported outcomes (PROs) of belimumab (versus placebo) in combination with rituximab and corticosteroids in participants with AAV
- To compare the effect on persistent damage of belimumab (versus placebo) in combination with rituximab and corticosteroids in participants with AAV

### Trial design

This experimental medicine, mechanistic, multicentre, randomised, double-blind, placebo-controlled trial aims to evaluate the effect of belimumab combined with rituximab on time to PR3 ANCA antibody negativity in participants with PR3 ANCA associated vasculitis (see Additional file 1 for the SPIRIT 2013 Checklist). The recruitment target is 30 patients who meet the criteria for inclusion in the per-protocol analysis. Thirty-six participants have been randomised to one of two treatment groups in a 1:1 ratio: either rituximab plus belimumab or rituximab plus placebo (both groups with the same tapering corticosteroid regimen), and recruitment is now closed (final patient enrolled April 2021). For each patient, the trial will last for 2 years comprising a 12-month treatment period followed by a 12-month follow-up period.

## Methods: participants, interventions and outcomes

### Study setting

Participants have been recruited from five of seven UK trial sites, including Cambridge University Hospitals NHS Trust (primary site), Imperial College London NHS Trust, University College London Hospitals NHS Foundation Trust, University Hospitals of Leicester NHS Trust and Nottingham University Hospitals NHS Trust.

### Eligibility criteria

The eligibility criteria include age ≥ 18 years and a diagnosis of AAV (according to Chapel Hill Consensus Conference definitions [13]) with active disease (newly diagnosed or relapsing disease) defined by one major or three minor disease activity items on the Birmingham Vasculitis Activity Score for Wegener's Granulomatosis (BVAS/WG) [14], along with a concurrent positive test for PR3 ANCA by ELISA. Proteinase 3 (PR3) ANCA was chosen instead of myeloperoxidase (MPO)-ANCA as this serotype is associated with deep tissue inflammation, slower time to remission and higher relapse rate. PR3 ANCA patients represent a subtype of AAV where maximal benefit may be gained by enhancing tissue B cell depletion [15]. Furthermore, MPO and PR3 AAV subgroups display clinical and genetic differences, and the inclusion of only PR3-positive patients would ensure greater homogeneity in this small experimental medicine study, which was considered important to allow interrogation of drug mechanisms [16].

The key exclusion criteria included MPO ANCA or anti-GBM antibody positivity, the presence of pulmonary haemorrhage with hypoxia and an estimated glomerular filtration rate (eGFR) < 15 mL/min/1.73 m^2^. Concerns about the impact of prior immunosuppression and/or glucocorticoids on mechanistic endpoints were balanced against the feasibility of being able to recruit sufficient numbers of patients, including some with potentially life-threatening active diseases. Thus, participants were permitted a maximum of 3 g intravenous (IV) methylprednisolone or an equivalent dose of oral prednisolone in the 30 days prior to enrolment and screening period, as deemed necessary by investigators to treat any major disease activity. The other exclusion criteria included receipt of any biological B cell-depleting agents within 12 months of screening or more than three doses of IV cyclophosphamide within 6 months of screening, patients with undetectable peripheral blood B cells at screening and IgG < 400 mg/dL at screening. Patients with acute or chronic infection or a history of malignancy within 5 years of screening were also excluded. Additional file 2 lists the full eligibility criteria for the study.

### Who will take informed consent?

Written informed consent was obtained by the site investigator or designee from each participant before any trial-specific activity was performed. The informed consent form was approved by the research ethics committee (REC) and was compliant with Good Clinical Practice (GCP), local regulatory requirements and legal requirements.

### Additional consent provisions for the collection and use of participant data and biological specimens

Consent was obtained for the collection and storage of biological samples within the University of Cambridge for up to 1 year after the end of the trial. After this 1-year period, samples will either be destroyed, transferred to an approved licenced storage facility (e.g. tissue bank) or transferred for use in a separate ethically approved research study. Consent was obtained for the storage of fully anonymised data collected during the trial in a secure database for up to 25 years and the sharing of trial data with GSK and other external agencies including regulatory bodies.

### Interventions

#### Explanation for the choice of comparators

Rituximab 1000 mg was administered by IV infusions on day 8 (± 3 days) and day 22 (± 3 days). This dose was selected because it causes rapid depletion of peripheral B cells below the lower limit of quantification by flow cytometry. Although published trials of rituximab in AAV used doses of 375 mg/m^2^ a week for four consecutive weeks, the use of 1000 mg repeated after 2 weeks is more practical and is a commonly adopted protocol. A retrospective review (*n* = 65) that compared the two regimens for AAV showed no difference in the duration of B cell depletion or disease-free interval [17]. Both dosing regimens are approved and commissioned for AAV by NHS England in the UK. Patients were recruited in line with the policy for use of rituximab for relapsing disease or new disease where cyclophosphamide is contraindicated [18].

Weekly subcutaneous (SC) injections of 200 mg belimumab or placebo were initiated a week before rituximab on day 1 and then weekly through to week 51. Belimumab 200 mg SC once weekly for 52 weeks was the dose and duration of therapy for the primary endpoint in the positive phase 3 trial of SC belimumab (BEL112341) in SLE [19]. The belimumab 200 mg SC weekly dose was selected because it delivers a substantial molar excess of belimumab above free BAFF levels in the blood, resulting in rapid suppression of serum BAFF levels and is understood to be equivalent (by area under the curve (AUC)) to the belimumab 10 mg/kg IV dose, which was originally approved for SLE based on efficacy and safety data alongside reductions in the autoantibody dsDNA. Furthermore, infections with belimumab plus standard of care were similar to that of placebo plus standard of care [19].

Given the observation of the immediate rise in circulating memory B cells after belimumab from prior treatment trial (within the first week of treatment) [20], rituximab was initiated 1 week after belimumab to effectively deplete the increased number of circulating memory B cells.

#### Intervention description

**Fig. 1:**
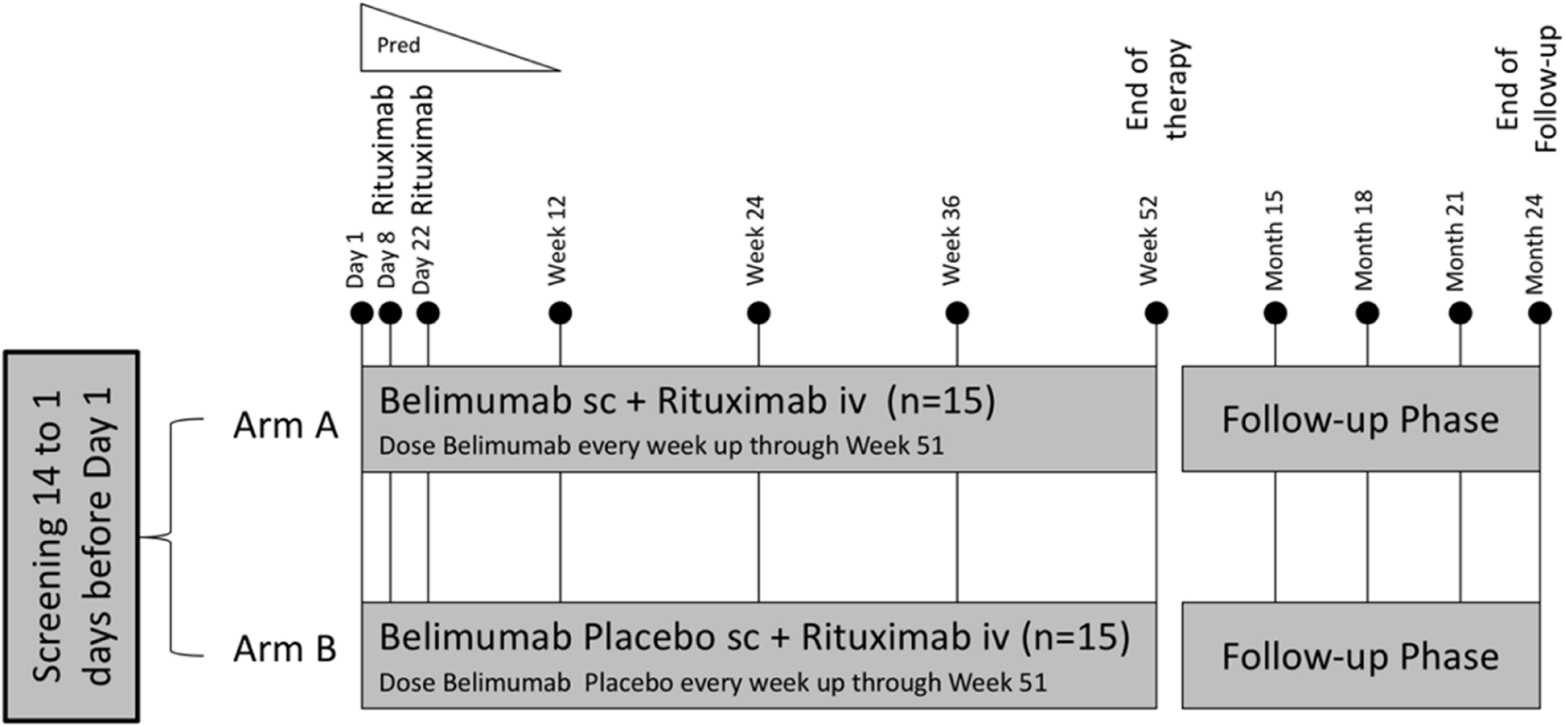
COMBIVAS study flow chart

The intervention description is presented in Fig. 1.

#### Criteria for discontinuing or modifying allocated interventions

Criteria for discontinuation from trial treatment include:

- Prohibited concurrent medication or therapy (including other investigational agents, co-enrolment into another trial of a different investigational agent, anti-TNF therapy, other biologics with effects on the immune system (e.g. abatacept, interleukin-1 receptor antagonist [anakinra]), IVIG, cyclophosphamide or plasmapheresis).
- Unacceptable toxicity in the opinion of the principal investigator.
- Participants who become pregnant.
- Non-compliance with trial treatment defined as missing 12 or more consecutive doses of belimumab/belimumab-placebo or > 50% of scheduled doses of belimumab/belimumab-placebo.
- Life-threatening infection (including progressive multifocal leukoencephalopathy (PML)).
- Severe skin reactions such as toxic epidermal necrolysis and Stevens-Johnson syndrome
- Liver dysfunction meeting the liver stopping criteria defined in Section 9.5.1.2 of the protocol.
- Presents with suicidal ideation of type 4 or 5 on the Columbia Suicide Severity Rating Scale (C-SSRS) or, in the investigator’s judgement, the participant is at risk for a suicide attempt.
- In case of worsening vasculitis (with major BVAS-WG items) before remission, trial treatment was discontinued and treatment started as per best local practice. If a participant developed a second minor or first major relapse after an initial remission, while on treatment, trial treatment was discontinued and subsequent treatment was delivered according to best local practice.

Participants who develop IgG < 250 mg/dL confirmed by a repeat test 1 week (at least > 2 days) after the initial result will have trial treatment withheld. Those with a decrease in IgG below 400 mg/dL confirmed by a repeat test 1 week after the initial result and associated with a serious infection (i.e. an infection reported as an SAE) will have trial treatment withheld. In addition, if any participant develops a life-threatening infection regardless of IgG status, trial treatment will be discontinued.

#### Strategies to improve adherence to interventions

The first dose of belimumab or belimumab-placebo was administered by the participant/their carer at the clinical site under the supervision and assistance of the trial nurse who provided training in the subcutaneous delivery technique. The date and time of the dose administered were recorded in the patient diary and reviewed by the study team at each visit.

#### Relevant concomitant care permitted or prohibited during the trial

In line with current practice favouring steroid avoidance/minimization strategies [21, 22] and to allow interrogation of drug mechanism which may be masked if the concomitant corticosteroid dose was too high, both groups received a relatively low prednisolone (20 mg/day) starting dose from day 1 followed by a protocol-specified corticosteroid taper aiming for complete cessation by 3 months (Table 1).

**Table 1:** Recommended prednisolone taper

Trial week	Prednisolone (mg/day)
0 (days 1–7)	20
1 (days 8–14)	20
2 (days 15–21)	15
3 (days 22–28)	15
4 (days 29–35)	15
5 (days 36–42)	10
6 (days 43–49)	10
7 (days 50–56)	10
8 (days 57–63)	5
9 (days 64–70)	5
10 (days 71–77)	5 every other day
11 (days 78–84)	5 every other day
12 (days 85–91)	0

#### Treatment of relapse

Relapse is defined as an increase in disease activity after remission (BVAS = 0) has been achieved. Major and minor relapses are differentiated by the presence or absence of major BVAS items, respectively. Participants experiencing a first minor relapse, after remission, at any time during the trial are permitted to receive prednisolone up to 30 mg/day for 1 week decreasing in 5 mg decrements every 2 weeks to reach 5 mg/day. A second minor relapse or first major relapse occurring at any time point during the trial triggers a withdrawal from protocol-assigned treatment and treatment according to best practice. Patients with new/worsening major disease manifestations prior to achieving remission (major progressive disease) also trigger a withdrawal from protocol-assigned treatment and best practice treatment for refractory disease.

#### Other therapies

Recommended prophylactic medications while on corticosteroids include bone protection with a bisphosphonate if post-menopausal and, in all patients, calcium and vitamin D supplements and gastric protection with a proton pump inhibitor. Pneumocystis jirovecii pneumonia (PJP) prophylaxis with co-trimoxazole is recommended for all patients for 12 months following trial entry.

#### Provisions for post-trial care

Participants are considered to have completed the trial once they have completed 12 months of the treatment phase and 12 further months of follow-up (i.e. month 24 trial visit). If a participant is withdrawn from trial treatment early, they will still be encouraged to attend the remaining visits and undergo the trial assessments as detailed in the schedule of activities (see Additional file 3). If the participants are not willing to continue attending the remaining trial visits, the end of the trial would be the 8 weeks post-last dose visit. Participants will return to their standard of care treatment pathway following their final trial visit.

### Outcomes

The primary endpoint of this study is time to PR3 ANCA negativity. B cell-derived ANCAs are implicated in disease pathogenesis and an effect on ANCA is of major mechanistic significance [23]. Because this study is investigating the differential effects of B cell monotherapy (rituximab) versus B cell sequential therapy (rituximab and belimumab), we predict not only differential effects in terms of magnitude, breadth and duration of B cell depletion, but also differential effects on B cell-derived ANCA. For PR3 ANCA, our own preliminary data (using the same ANCA assay, in a similar patient population, receiving the same control rituximab regimen) suggests that time to PR3 ANCA negativity is long (14 months median) [24]. With sequential therapy, we predict that ANCA levels will fall quicker. Achieving PR3 ANCA negativity is associated with clinical remission. The binary endpoint of time to ANCA negativity was chosen for the primary endpoint, to reflect both clinical and mechanistic importance.

#### Primary outcome

- ➢ Time to PR3 ANCA negativity (ELISA)

#### Secondary outcomes

- ➢ Proportion of participants with ANCA negativity (ELISA) at months 3, 6, 12, 18 and 24
- ➢ Proportion of participants with sustained PR3 ANCA negativity at month 12 and month 24
- ➢ Change in PR3 ANCA level from baseline to months 3, 6, 12, 18 and 24
- ➢ Time to PR3 ANCA < 5 iU/L
- ➢ Time to PR3 ANCA < 10 iU/L
- ➢ Time to 50% fall in PR3 ANCA (from baseline)
- ➢ Time to rise in PR3 ANCA (relative increase of at least 25% from the lowest measured level and an absolute increase of at least 10 iU/L)
- ➢ Absolute and percentage change from baseline in CD4 and CD8 T cells, B cells (CD19) and natural killer cells in the blood at months 3, 12, 18 and 24
- ➢ Change from baseline in naïve, transitional, memory, activated, plasmablast subsets in the blood at months 3, 12, 18 and 24
- ➢ Time to clinical remission (as measured by BVAS/WG at baseline, months 1–6, 9, 12, 15, 18, 21 and 24)
- ➢ Proportion of participants in complete remission at months 6, 12 and 24
- ➢ Time to first relapse (major or minor), as measured by BVAS/WG at baseline, months 1–6, 9, 12, 15, 18, 21 and 24 and at unscheduled (relapse) visits or prohibited medication in those who have achieved remission
- ➢ Time to first relapse (major only, as measured by BVAS/WG at baseline, months 1–6, 9, 12, 15, 18, 21 and 24 and at unscheduled (relapse) visits or prohibited medication in those who have achieved remission)
- ➢ Proportion of participants with progressive disease before remission (major or minor)
- ➢ Proportion of participants with progressive disease before remission (major only)
- ➢ Incidence of severe adverse events (SAEs)

Incidence and severity of AEs of special interest (AESIs): all infections requiring antimicrobial, antiviral or antifungal treatment, hypogammaglobulinaemia (IgG < 400 mg/dL (grade 3) and < 250 mg/dL (grade 4)), systemic infusion/injection reactions, hypersensitivity reactions, malignancy, psychiatric events (including suicidality), severe skin reactions (including toxic epidermal necrolysis and Stevens-Johnson syndrome), cardiac disorders (including angina, myocardial infarction, arrhythmia, heart failure), thromboembolic events, posterior reversible encephalopathy (PRES) and pregnancy.

#### Participant timeline

See Additional file 3 for the schedule of activities.

#### Sample size

Since the study is exploratory in nature, the sample size is based only on feasibility. However, the sample size can be justified statistically, using an assumption regarding treatment effect size. Rituximab treatment is known to be associated with falls in PR3 ANCA levels and clinical remission. Our own preliminary data from AAV patients treated with rituximab found a median time to PR3 ANCA ELISA negativity of 14 months. With a two-sided 5% type I error rate, 15 patients per arm results in 80% power to detect a difference when the rituximab-belimumab group has a median time to PR3 ANCA negativity of 3 months. This is calculated assuming a log-rank test using the following formula for a two-sided test with type I error rate *α* and power 1 − *β*:

$$n = \frac{{\left( {Z_{{\frac{\alpha }{2}}} + Z_{\beta } } \right)^{2} }}{{d\log^{2} \lambda }}$$

where *Z*_*α*/2_ and Z_*β*_ are the quantiles of the standard normal distribution, *λ* is the hazard ratio and *d* is the probability a participant in either group will eventually have an event.

No sample size re-estimation was planned for this trial. However, prior to cessation of recruitment, protocol adherence was assessed, and furthermore, participants were recruited to ensure approximately 30 participants would be evaluable by a per-protocol analysis.

#### Recruitment

Thirty-six patients have been recruited from 5 UK sites between February 2019 and April 2021 (see Additional file 4). In March 2020, the UK government introduced a national lockdown to limit the spread of COVID-19. Recruitment was temporarily paused while risk assessments were performed, and trial procedures were modified to ensure the safety of participants, including telephone assessments when face-to-face assessments are not deemed clinically essential, couriering belimumab/placebo to patients’ homes and use of blood testing in facilities local to patients’ homes with the shipment of samples, staff to wear PPE and adhere to social distancing policies for face-to-face assessments and COVID-19 screening swabs to be performed before all nasal biopsy procedures. Recruitment was restarted in June 2020 following sponsor and Data and Safety Monitoring Board (DSMB) approval of trial adaptations for patient safety during the pandemic. During the pandemic, every effort was made to adhere to visit time windows; however, a pragmatic approach was adopted allowing greater flexibility of study windows when needed to avoid missing samples during the pandemic. COVID-19 vaccinations were introduced in the UK in 2021. Participants were encouraged to have vaccinations at the earliest opportunity. All vaccines and infections are captured in the trial database. Sensitivity analyses will be performed to assess the impact of infections and vaccinations on trial outcomes.

Several samples from key time points were not collected at the start of the pandemic when the research laboratories were unable to function at full capacity. To minimise the potential impact caused by missed time points on the primary endpoint analysis, PR3 ANCA results from trial samples will be combined with PR3 ANCA values performed during routine clinical care using the same assay.

### Assignment of interventions: allocation

#### Sequence generation

All patients screened for the trial were assigned a unique trial ID number, and eligible participants were assigned to trial treatment in accordance with the randomisation schedule. Randomisation of participants was stratified by screening PR3 ANCA levels (low vs. high by median value). A randomisation schedule was generated such that within each stratum, participants were randomised to one of the two following treatment assignments in a 1:1 ratio using an online randomisation system (Sealed Envelope) accessible via password-protected access:

**Table:** 

Arm	Treatment description
Sequential	Belimumab SC weekly for 52 weeks + rituximab at days 8 and 22
Control	Belimumab placebo SC weekly for 52 weeks + rituximab at days 8 and 22

#### Concealment mechanism and implementation

Access to the web-based randomisation system (Sealed Envelope) was given via individual user accounts provided to the site investigator and delegated members of the research team at each site. Immediate allocation of treatment was performed, with documentation of the decision in a confirmatory email. The system allocated the participant a medication pack code number at each dispensing which related uniquely to a supply of the investigational medicinal product (IMP) that was held at that trial site.

### Assignment of interventions: blinding

#### Who will be blinded

This is a double-blind trial in which neither the investigator, nor the subject has knowledge of which study treatment is being administered. The independent members of the DSMB are unblinded and perform safety reviews during the conduct of the trial. Safety data is provided by the unblinded trial coordinator. The trial statistician is blinded throughout the conduct of the trial.

It is not possible for the study team to determine treatment allocation using the laboratory data generated during the trial period. Laboratory data with the potential to unblind trial staff (e.g. serum BAFF) will only be measured at the end of the trial.

#### Procedure for unblinding if needed

The online Sealed Envelope randomisation system may be used for emergency unblinding.

### Data collection and management

#### Plans for assessment and collection of outcomes

A screening period of up to 14 days is followed by evaluations (including routine blood and urine tests, clinical assessments, safety assessments and patient-reported outcomes) performed monthly for the first 6 months, 2 monthly for the second 6 months and 3 monthly for the final 12 months. Assessments are also performed at the time of a second minor or major relapse, or withdrawal from the study drug for safety/other reasons.

#### Trial-specific biomarker assessments

Blood and urine samples are taken at multiple time points, with key analyses at 3 months (time of maximal B cell depletion), 12 months (end of treatment phase) and 24 months (time of maximal B cell reconstitution and end of follow-up). A key focus of this study is understanding the mechanistic effects of belimumab-rituximab sequential therapy in tissues. For this purpose, patients provided consent for paired nasal tissue biopsies and inguinal lymph node biopsies which were performed before initiating study treatment on day 1 (or during screening) and again at 3 months. Sino-nasal inflammation is a common manifestation of AAV [25]. Therefore, the nasal mucosa offers a disease-relevant and easily accessible site for tissue biopsies. Likewise, biopsy samples from the inguinal lymph nodes are safe to perform and permit direct assessment of T cell–B cell interaction and humoral responses within a secondary lymphoid organ. It is anticipated that biopsy data will be available for a subset of patents allowing for the withdrawal of consent or insufficient sampling in a minority of patents. The 3-month time point was chosen as the time of likely maximum B cell depleting effect of the sequential therapies based on the knowledge of rituximab pharmacokinetics [26], minimal ongoing corticosteroids exposure and the time at which clinical remission is usually expected (Table 2).

**Table 2:** Biomarker analyses

Compartment	Biomarker	Endpoint
Blood	➢ PR3 ANCA (ELISA) ➢ Lymphocyte immunophenotyping (flow cytometry) ➢ B cell receptor clonality, B cell and T cell functional assays, and whole blood transcriptomic analysis ➢ BAFF protein, BAFF-belimumab complex and cytokines	Primary Secondary Exploratory Exploratory
Urine	➢Lymphocyte immunophenotyping (flow cytometry) ➢Proteome analysis	Exploratory Exploratory
Tissue biopsies (subset of patients only)	➢Lymphocyte immunophenotyping (flow cytometry) ➢Single cell RNA sequencing	Exploratory Exploratory

The PR3 ANCA ELISA assay (EliA fluoro enzyme immune assay test reagents and the Phadia instrument 2500/5000) for the primary endpoint was validated before the trial. Samples were sent from participating sites to Cambridge via overnight shipping resulting in a delay in sample processing. As the viability of some B cell subsets is known to decline quickly after venepuncture, blood was collected in cell stabilisation tubes (CytoChex BCT) for secondary endpoint analyses. Pre-trial validation experiments were performed to verify the performance characteristics of custom flow cytometry assays designed to evaluate the measurement of B cell subsets in the whole blood collected in CytoChex BCT tubes. Pre-trial feasibility testing was also performed on the urine processing and analysis techniques and for the tissue biopsies which demonstrated acceptable tolerability and safety profile for both local anaesthetic biopsy techniques [27].

#### Plans to promote participant retention and complete follow-up

Participants who discontinue trial treatment early and/or start rescue therapy and/or withdraw from the trial should complete the following visits, wherever possible. Participants who discontinue trial treatment before week 52 but remain in the trial:

- Early withdrawal end of therapy visit 1 week ± 6 days after the last dose of belimumab/belimumab-placebo (prior to any rescue therapy if initiated)
- Continue remaining visits from the trial visit schedule

Participants withdrawing consent at any time, irrespective of reason will be encouraged to complete:

- Early withdrawal end of therapy visit 1 week ± 6 days after the last dose of belimumab/belimumab-placebo if discontinuing treatment before week 52
- Eight weeks post-last dose safety visit where the withdrawal occurs within the first 15 months

Participants who complete therapy and commence rescue therapy during follow-up but remain in the trial:

- Unscheduled visit at relapse (prior to rescue therapy initiation)
- Continue remaining visits from the trial visit schedule

In the event that a participant discontinues the trial or withdraws consent, an attempt will be made to ascertain survival status at approximately 52 weeks and 104 weeks after the first dose of trial treatment.

#### Data management

The sponsor or designee is responsible for the data management of this trial including quality checking of the data. Trial monitors perform ongoing source data verification and that the trial is being conducted in accordance with the currently approved protocol, GCP and all applicable regulatory requirements.

#### Confidentiality

All investigators and trial site staff involved in this trial must comply with the requirements of the EU General Data Protection Regulation (GDPR) and Trust Policy with regard to the collection, storage, processing, transfer and disclosure of personal information and will uphold the act’s core principles. Participants were assigned a unique identifier by the trial team upon enrolment to the trial. Any participant records or datasets that are transferred to the sponsor and/or IMP manufacturer, GSK (including SAE/SUSAR reports) contain the identifier only.

#### Plans for collection, laboratory evaluation and storage of biological specimens for genetic or molecular analysis in this trial/future use

Samples have been collected for bulk RNA sequencing on all participants (blood only at day 1, months 3, 12, 18 and 24) and single-cell RNA sequencing (scRNA-seq) on a subset of participants (blood and tissue biopsies at day 1 and month 3). Frozen samples for bulk RNA sequencing are currently stored in PaxGene RNA tubes at the University of Cambridge. ScRNA-seq libraries were generated at the University of Cambridge and sequenced by Genewiz (Leipzig, Germany). Genetic data will be uploaded onto a publicly accessible database as a supplement to the publication of this study.

### Statistical methods

#### Statistical methods for primary and secondary outcomes

Additional file 5 details the full statistical analysis plan for the study. Briefly, the primary endpoint of time to PR3 ANCA negativity will be compared between the sequential arm and the control arm using the Cox proportional hazard model with the *Exact* method for handling any ties and with covariates for screening PR3 ANCA levels (low vs. high by median value) and treatment group (i.e. belimumab vs. placebo). The median time to PR3 ANCA negativity will be presented for each randomised treatment group, along with the estimated hazard ratio (belimumab:placebo) of the treatments, its 95% confidence intervals and associated *p*-value. *p*-values will be based on the likelihood ratio test, and confidence intervals will use a profile likelihood approach. A HR less than 1 favours belimumab + rituximab.

Regarding key secondary endpoints, change from baseline in PR3 ANCA will be analysed using a Wilcoxon rank sum test. The Hodges-Lehmann method will be used to provide estimates of the median differences and non-parametric 95% confidence intervals at 3, 6, 12, 18 and 24 months. To assess the change from baseline in naïve, transitional, memory, activated and plasmablast subsets (using high sensitivity B cell flow cytometry) in the blood (3, 12, 24 months), the per cent change from baseline data will be compared between the belimumab treatment group and placebo group using a Wilcoxon rank sum test. A Hodges-Lehmann method will be used as described above. Time to clinical remission and time to first relapse will be analysed using the methods described for the primary endpoint.

#### Interim analyses

There are no planned interim analyses.

#### Methods in analysis to handle protocol non-adherence and any statistical methods to handle missing data

All randomised participants who receive at least one dose of belimumab/belimumab placebo will be included in the intention-to-treat analysis. However, as the aim of this experimental medicine study is to interrogate drug effect within the trial setting, the primary analysis will only include the pre-defined ‘evaluable’ participants as a per protocol analysis. The criteria for evaluable participants will include:

- ➢ Three months of belimumab/belimumab-placebo and ≥ 8 injections in the first 12 weeks.
- ➢ No contraindicated medications (immunosuppressive/immunomodulatory).
- ➢ Compliance with steroid taper (no more than one episode of temporary increase in corticosteroids above the scheduled steroid taper during the first 3 months of trial). Beyond 3 months, the average daily prednisolone dose should be 0 (± 10) mg/day and no more than one temporary increase in prednisolone for treatment of minor flare.
- ➢ Excluding participants with major protocol deviations with the potential to impact mechanistic efficacy assessments.

#### Plans to give access to the full protocol, participant-level data and statistical code

The full protocol, datasets and statistical code will be available from the corresponding author upon reasonable request.

### Oversight and monitoring

#### Composition of the coordinating centre and trial steering committee

The trial management committee (TMC) is responsible for the design, conduct and overall management of the study. The members meet on a regular basis in Cambridge. The trial steering committee (TSC) provide overall supervision of the study on behalf of the MRC-EMINENT programme. The TSC includes members of the TMC and three independent physicians with experience in vasculitis trials. The TSC meet regularly via teleconference and monitors the progress of the study and maximise the chances of completing the study within the agreed time scale and budget.

#### Composition of the data monitoring committee, its role and reporting structure

A Data and Safety Monitoring Board will provide oversight of this trial. The DSMB membership consists of three independent physicians with experience in vasculitis trials and two statisticians. It is independent from the sponsor and competing interests. Summary reports on recruitment, data quality, safety data and clinical efficacy endpoints are compiled by the trial statisticians and provided to the DSMB who meet approximately every 6 months. The DSMC does not make decisions about the trial, but rather makes recommendations to the TSC.

#### Adverse event reporting and harms

All serious adverse events are reported by the site investigators to the chief investigator within 24 h of their awareness of the event. Onward notification to the sponsor occurs no more than 24 h after the first notification. All AESIs are reported by the site investigators to the chief investigator within 14 calendar days of their awareness of the event. If the AESI is deemed to be serious, then the reporting procedure for an SAE should be followed (i.e. reported within 24 h).

#### Plans for communicating important protocol amendments to relevant parties (e.g. trial participants, ethical committees)

The sponsor has delegated responsibility for preparing and submitting amendments to the trial team. Substantial protocol amendments were reviewed by GSK prior to submission to the research ethics committee (REC).

#### Dissemination plans

The results of this trial will be published and presented at scientific meetings. A results summary will also be posted to publicly available clinical trial registers and a manuscript developed for publication in a peer-reviewed journal after the completion of the trial.

## Discussion

COMBIVAS is an experimental medicine study that encompasses a series of mechanistic investigations within blood, urine, lymph node and nasal tissue biopsies from AAV patients receiving sequential B cell targeting therapy with belimumab and rituximab compared to patients receiving rituximab alone. This study provides a unique opportunity to gain a detailed insight into the immunological mechanisms within tissues driving relapse and how synergistic effects of two B cell targeting monoclonal antibodies in combination may lead to long-lasting remission. The proposed mechanistic investigations rely heavily on techniques within the University of Cambridge, University College London, Imperial College London and GlaxoSmithKline (GSK). Without this collaborative scientific experience, such detailed mechanistic studies would be impractical. A barrier to the research of B cell-targeted therapy has been the difficulty in obtaining sequential cells from sites where the immune dysregulation occurs or sites of inflammation. The inclusion of both lymph node biopsies and nasal tissue biopsies in this trial will potentially permit the direct characterisation of pathogenic cells at key sites, their microenvironment and, critically, the interaction of B cells with T follicular helper cells, the primary drivers of the abnormal immune response.

The GSK MRC EMINENT programme has provided a unique opportunity to bring together vasculitis specialists from major UK institutions with GSK researchers facilitating a collaborative approach to scientific investigations and rapid recruitment of rare disease patients from specialist clinics. Combining the disease biology expertise of academic institutions with scientific expertise and access to therapies within the industry provides the opportunity for advancing scientific understanding of novel drug mechanisms in rare diseases such as AAV.

## Trial status

The final patient was recruited in April 2021. The last subject’s last visit will be in April 2023. A two-staged publication strategy is planned consisting of an initial report of the early mechanistic biomarkers of B cell depletion including (1) exploratory evaluation of the early pharmacodynamic effects of belimumab on B cells and B cell subsets within the first week before any rituximab administration and (2) exploratory analysis of baseline and month 3 blood, nasal biopsies, lymph node biopsies by flow cytometry and single-cell RNA sequencing, followed by the full study report when all patents have completed trial follow-up (primary and secondary endpoint analyses and remaining exploratory analyses).

Due to significant uncertainties over recruitment and trial conduct during the COVID-19 pandemic, publication of this manuscript was delayed until recruitment was complete and subsequent external and internal collaborator reviews had been undertaken, in order to provide a manuscript that was reflective of the final protocol.

## Supplementary Information

**Additional file 1.** SPIRIT 2013 Checklist.

**Additional file 2.** Full inclusion and exclusion criteria.

**Additional file 3.** Schedule of Activities.

**Additional file 4.** COMBIVAS recruitment graph.

**Additional file 5.** Statistical Analysis Plan.

**Additional file 6.** Participant information sheet summary.

## Data Availability

Data sharing is not applicable to this article as no datasets were generated or analysed during the current study. Any data required to support the protocol can be supplied on request.

## Abbreviations

AAV: ANCA-associated vasculitis
AESI: Adverse events of special interest
AUC: Area under the curve
BAFF: B cell activating factor
BREVAS: Belimumab in Remission of Vasculitis trial
BVAS-WG: Birmingham Vasculitis Activity Score for Wegener's Granulomatosis
CCTU: Cambridge Clinical Trials Unit
DSMB: Data and Safety Monitoring Board
eCRF: Electronic case report form
eGFR: Estimated glomerular filtration rate
ELISA: Enzyme-linked immunosorbent assay
EMINENT: Experimental Medicine Initiative to Explore New Therapies
GBM: Glomerular basement membrane
GCP: Good Clinical Practice
GDPR: General Data Protection Regulation
GSK: GlaxoSmithKline
Ig: Immunoglobulin
IV: Intravenous
IVIG: Intravenous immunoglobulin
IMP: Investigational medicinal product
MPO: Myeloperoxidase
MRC: Medical Research Council
PJP: Pneumocystis jirovecii pneumonia
PML: Progressive multifocal leukoencephalopathy
PR3: Proteinase 3
PRES: Posterior reversible encephalopathy
PROs: Patient-reported outcomes
REC: Research Ethics Committee
SAE: Serious adverse event
SC: Subcutaneous
scRNA-seq: Single-cell RNA sequencing
SLE: Systemic lupus erythematosus
SUSAR: Suspected unexpected serious adverse event
TMC: Trial management committee
TSC: Trial steering committee
