# Supplementary material for: A randomised study of rituximab and belimumab sequential therapy in PR3 ANCA-associated vasculitis (COMBIVAS): design of the study protocol
Source: Trials. 2023 Mar 11;24:180. doi: 10.1186/s13063-023-07218-y (PMC10007661; doi:10.1186/s13063-023-07218-y)
Supplement: Supplementary file 2 — Additional file 2. Full inclusion and exclusion criteria. [file 13063_2023_7218_MOESM2_ESM.docx]

Additional file [2](https://trialsjournal.biomedcentral.com/articles/10.1186/s13063-017-1857-z#MOESM2): Full inclusion and exclusion criteria

**Inclusion Criteria**

Participants are eligible to be included in the trial only if all of the following criteria apply:

1. Participant must be ≥18 of age at the time of signing the informed consent form.

2. Have a diagnosis of AAV (granulomatosis with polyangiitis or microscopic polyangiitis), according to the definitions of the Chapel Hill Consensus Conference

3. PR3 ANCA positivity by ELISA at screening

4. Have active disease defined by one major or three minor disease activity items on the Birmingham Vasculitis Activity Score for Wegener’s (BVAS/WG)

5. Women of child-bearing potential (WOCBP) must agree to use effective contraception methods and agree to follow these methods during the treatment period and for at least 16 weeks after the last dose of belimumab-placebo, or at least 12 months after the last dose of rituximab, whichever is greater.

6. Capable of giving signed informed consent

**Exclusion Criteria**

Participants are excluded from the trial if any of the following criteria apply:

1. Women who are pregnant, plan to become pregnant or are lactating during the trial period

2. Participation in any other investigational treatment trials unless observational in nature

3. MPO ANCA or anti–GBM antibody positivity by ELISA

4. Presence of pulmonary haemorrhage with hypoxia

5. Estimated glomerular filtration rate (eGFR) <15 ml/min/1.73m2

6. Symptomatic herpes zoster within 3 months prior to screening

7. Evidence of active or latent tuberculosis (TB) as documented by medical history and examination, chest X-rays (if required as part of SoC), and TB testing: a positive (not indeterminate) QuantiFERON®-TB Gold test (or equivalent)

8. Significant allergies to humanised monoclonal antibodies

9. Clinically significant multiple or severe drug allergies and/or history of hypersensitivity to belimumab and/or rituximab or known to have titres of human anti-mouse antibody or history of hypersensitivity reactions when treated with other diagnostic or therapeutic monoclonal antibodies

10. Malignant neoplasm within the past 5 years (from screening) with the exception of basal cell or squamous epithelial carcinoma of the skin treated with local resection only or carcinoma in situ of the uterine cervix treated locally and with no evidence of metastatic disease for 3 years.

11. Have a history of a primary immunodeficiency

12. IgA deficiency (IgA level < 10 mg/dL)

13. IgG < 400 mg/dL

14. Neutrophils < 1.5 x 109 cells/L

15. Pre-existing B cell lymphopenia (total CD19+ count < lower limit of detection)

16. Alanine transferase (ALT) >2x upper limit of normal (ULN)

17. Bilirubin >1.5xULN (isolated bilirubin >1.5xULN is acceptable if bilirubin is fractionated and direct bilirubin <35%)

18. Current or chronic history of liver disease, or known hepatic or biliary abnormalities (with the exception of Gilbert's syndrome or asymptomatic gallstones)

19. Active bleeding disorders, and/or inability to support interruption to anticoagulant (including but not limited to warfarin, rivaroxaban) or anti-platelet therapies (including but not limited to aspirin, clopidogrel, etc.), as assessed jointly by trial physician and physician responsible for managing anticoagulant / anti-platelet therapies

20. Severe heart failure (New York Heart Association Class IV) or other severe, uncontrolled cardiac disease

21. QT >450 msec or QTc >480 msec in participants with bundle branch block

22. Have a history of a major organ transplant (e.g., heart, lung, kidney, liver) or hematopoietic stem cell/marrow transplant

23. Have an acute or chronic infection requiring management as follows:

- Currently on any suppressive therapy for a chronic infection such as pneumocystis, cytomegalovirus, herpes simplex virus, herpes zoster, or atypical mycobacteria)
- Hospitalisation solely for treatment of proven infection within 60 days of Day 1
- Have had proven severe infection requiring treatment with parenteral (IV or IM) antibiotics (antibacterials, antivirals, antifungals, or anti-parasitic agents) within 60 days of Day 1. Prophylactic anti-infective treatment is allowed. Precautionary PO/IV antibiotics in a patient with active vasculitis will be permitted.

24. Have evidence of serious suicide risk, including any history of suicidal behaviour in the last 6 months and/or any suicidal ideation of type 4 or 5 on the C-SSRS in the last 2 months or who, in the investigator's opinion, pose a significant suicide risk

25. Have clinical evidence of significant unstable or uncontrolled acute or chronic diseases not due to vasculitis (i.e., cardiovascular, pulmonary, haematological, gastrointestinal, hepatic, renal, neurological, malignancy, or infectious diseases) which, in the opinion of the principal investigator, could confound the results of the trial or put the participant at undue risk

26. Have a planned surgical procedure, laboratory abnormality, or condition (e.g., poor venous access) that, in the opinion of the principal investigator, makes the participant unsuitable for the trial.

Prior/Concomitant Therapy

27. Live vaccine(s) within 30 days prior to Day 1, or plans to receive such vaccines during the treatment phase

28. Have received anti-CD52 [alemtuzumab] within 5 years of Day 1 (or total CD3 count <0.7 x109/L) at screening following previous anti-CD52 [alemtuzumab] exposure.

29. Have received any of the following within 180 days of Day 1:

Belimumab

Rituximab

- Any B cell targeted therapy (anti-CD20 agents other than rituximab, anti-CD22 [epratuzumab], BLyS-receptor fusion protein [BR3], TACI Fc, anti-BAFF (LY2127399), anti-Interferon alpha agents or anti-BLyS other than belimumab)
- A biologic investigational agent other than B cell targeted therapy or abatacept (e.g., abetimus sodium, anti CD40L antibody [BG9588/ IDEC 1311]). (Investigational agent applies to any drug not approved for sale in the country in which it is being used.)

30. Have received any of the following within 90 days of Day 1:

- More than 3 infusions of cyclophosphamide
- Anti-TNF or anti-IL-6 therapy (e.g., adalimumab, etanercept, infliximab, tocilizumab)
- Abatacept
- Interleukin-1 receptor antagonist (e.g., anakinra)
- Intravenous immunoglobulin (IVIG)
- Plasmapheresis, leukapheresis.

31. Have received a non-biologic investigational agent (investigational agent applies to any drug not approved for sale in the country in which it is being used) within 60 days of Day 1.

32. Have received any steroid injection (e.g., intramuscular [IM], intraarticular, or IV) within 60 days of Day 1 (unless given during or 30 days before screening period)

33. Have received emergency steroid >3g methylprednisolone (IV) or equivalent dose of oral prednisolone between 30 days prior to Screening Visit and Day 1 (including Day 1) - (please see section 10.2.2 for further information).

34. Positive human immunodeficiency virus (HIV) antibody test

35. Positive serology for Hepatitis B (HB), defined as: (i) HB surface antigen positive (HBsAg+) OR (ii) HB core antibody positive (HBcAb+)

36. Positive Hepatitis C (HCV) antibody test

37. Participants with current drug/alcohol dependence or drug/alcohol dependence within the last 6 months prior to Day 1

38. Compliance: is unlikely to comply with scheduled trial visits based on investigator judgment or has a history of substance abuse, psychiatric disorder or condition that may compromise communication with the investigator

39. Sensitivity to any of the trial treatments, or components thereof, or drug or other allergy that, in the opinion of the investigator, contraindicates participation in the trial

40. Unable to administer trial treatment (belimumab/ belimumab-placebo) by SC injection and has no other reliable resource to administer the injection

41. Unable to safely and reliably store trial treatment (belimumab/ belimumab-placebo). E.g., no permanent residence, no fridge
