# Supplementary material for: A randomised study of rituximab and belimumab sequential therapy in PR3 ANCA-associated vasculitis (COMBIVAS): design of the study protocol
Source: Trials. 2023 Mar 11;24:180. doi: 10.1186/s13063-023-07218-y (PMC10007661; doi:10.1186/s13063-023-07218-y)
Supplement: Supplementary file 3 — Additional file 3. Schedule of Activities. [file 13063_2023_7218_MOESM3_ESM.pdf]

Table 3 Treatment and follow-up period

Procedure	Year 1 – Treatment Period																Year 2 – Follow-up				Unscheduled visit[14,15]	Early Withdrawal[16]	
Trial Day[1]	1	8	22	29	57	85	113	141	169	197	225	253	281	309	337	365	456	547	638	729		End of Therapy visit[17]	8 week post last dose
Trial Week[1]		1	3	4	8	12	16	20	24	28	32	36	40	44	48	52	65 (M15)	78 (M18)	91 (M21)	104 (M24)			
Visit to trial centre	x	x	x	x	x	x	x	x	x	x		x		x		x	x	x	x	x	x	x	x
Randomisation[2]	x																						
Training on use of prefilled syringe	x																						
Dispense Belimumab / belimumab-placebo[3]	x			x	x	x	x	x	x	x		x		x									
Rituximab (IV)[4]		x	x																				
AE/SAE review[5,6]	x	x	x	x	x	x	x	x	x	x	x[6]	x	x[6]	x	x[6]	x	x	x	x	x	x	x	x
Concomitant medication review	x	x	x	x	x	x	x	x	x	x		x		x		x	x	x	x	x	x	x	x
Dispense / review patient diaries	x			x	x	x	x	x	x	x		x		x		x							
Return patient injection diary																x							
AAV-PRO[7]	x					x			x							x		x		x		x	
BVAS/WG	x			x	x	x	x	x	x	x		x		x		x	x	x	x	x	x	x	
Vasculitis Damage Index (VDI)	x								x							x		x		x		x	
C-SSRS	x	x	x	x	x	x	x	x	x	x		x		x		x	x	x	x	x	x	x	x
Symptom-driven physical exam	x	x	x	x	x	x	x	x	x	x		x		x		x	x	x	x	x	x	x	x
Neurological Assessment	x	x	x	x	x	x	x	x	x	x		x		x		x	x	x	x	x	x	x	x
Urine pregnancy test (WOCBP only)[8]	x			x	x	x	x	x	x	x	x[8]	x	x[8]	x	x[8]	x	x	x			x	x	
Urine dipstick, urine microscopy, UPCR	x			x	x	x	x	x	x	x		x		x		x	x	x	x	x	x	x	

Procedure	Year 1 – Treatment Period																Year 2 – Follow-up				Unscheduled visit[14,15]	Early Withdrawal[16]	
**Trial Day[1]**	1	8	22	29	57	85	113	141	169	197	225	253	281	309	337	365	456	547	638	729		End of Therapy visit[17]	8 week post last dose
**Trial Week[1]**		1	3	4	8	12	16	20	24	28	32	36	40	44	48	52	65 (M15)	78 (M18)	91 (M21)	104 (M24)			
Visit to trial centre	x	x	x	x	x	x	x	x	x	x		x		x		x	x	x	x	x	x	x	x
Routine bloods[9,10]	x			x	x	x[10]	x	x	x	x		x		x		x	x	x	x	x	x	x	x
PR3 ANCA (research sample)	x			x	x	x	x	x	x	x		x		x		x	x	x	x	x	x[15]	x	
Blood leukocyte analysis	x	x				x										x		x		x	x[15]	x	
Transcriptomics (whole blood)	x					x										x		x		x	x[15]	x	
Exploratory blood biomarkers	x	x[11]				x										x		x		x	x[15]	x	
Urine proteomics	x					x										x				x	x[15]	x	
Urine lymphocytes	x					x										x				x	x[15]	x	
BLyS, BLyS-Beli complex, cytokines	x					x			x			x				x	x	x	x	x	x[15]	x	
Lymph node and nasal biopsy (if consented)[12,13]	x					x																	
Nasal swab (microbiome)	x					x																	

Footnotes:

1. Visits for Day 8, Day 22 and Week 4 must occur within a +/-3day window of the scheduled visit. Visits for Week 8 to Week 52 must occur within a +/-7day window of the scheduled visit. Visits for Month 15 to Month 24 must occur within a +/-14-daywindow of the scheduled visit.
2. Randomisation can occur on D1 or during screening period after all eligibility criteria have been met.
3. All samples must be taken before first belimumab/belimumab-placebo. First dose to be given in clinic under supervision with 3 hours observation post-dose. Thereafter, the patient will self-administer at home on weekly basis. Patients will be provided with sufficient doses of belimumab/belimumab-placebo to cover the time between scheduled visits.
4. **The 2nd and 4th dose of belimumab should not be given on the same day as rituximab. Administration of each drug must remain within the defined time window (belimumab: Day 8 and 22 +/-1day; rituximab: Day 8 and 22 +/-3days).** On Days of Rituximab infusion, pre-medication will be administered 30 minutes prior to infusion. Observation is required for 1 hour post dose.
5. Please note: recording of all adverse events must start from the point of informed consent regardless of whether a participant has yet received a medicinal product.
6. At Weeks 32, 40, 48, when no visit is scheduled, site must telephone the participants to check for AE/SAE (including suicidal ideation) and if needed arrange an unscheduled visit.
7. AAV-PRO should be completed prior to other clinical assessments.
8. Home urine pregnancy test on weeks 32, 40 and 48, with telephone follow-up. Pregnancy testing is only mandated for WOCBP up until 16 weeks post last MP dose.
9. Routine bloods include FBC (incl. WCC with differential), urea, creatinine, eGFR, sodium, potassium, bilirubin, ALT (or AST), ALP, CRP, ESR, glucose, immunoglobulins.
