# Supplementary figures and images for: A randomised study of rituximab and belimumab sequential therapy in PR3 ANCA-associated vasculitis (COMBIVAS): design of the study protocol
Source: Trials. 2023 Mar 11;24:180. doi: 10.1186/s13063-023-07218-y (PMC10007661; doi:10.1186/s13063-023-07218-y)

Additional file 4: COMBIVAS recruitment graph


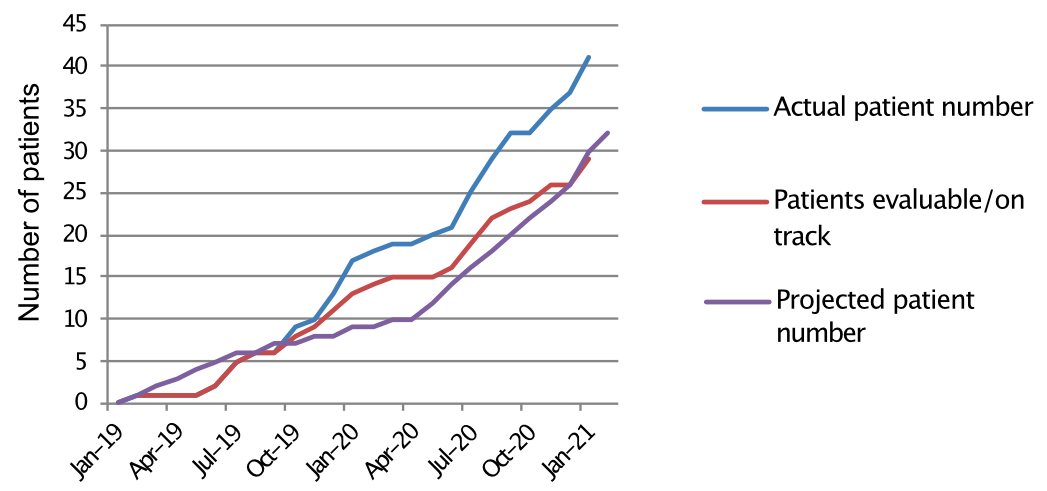

Supplement: Supplementary file 4 — Additional file 4. COMBIVAS recruitment graph. [file 13063_2023_7218_MOESM4_ESM.docx]
