# Supplementary material for: A randomised study of rituximab and belimumab sequential therapy in PR3 ANCA-associated vasculitis (COMBIVAS): design of the study protocol
Source: Trials. 2023 Mar 11;24:180. doi: 10.1186/s13063-023-07218-y (PMC10007661; doi:10.1186/s13063-023-07218-y)
Supplement: Supplementary file 5 — Additional file 5. Statistical Analysis Plan. [file 13063_2023_7218_MOESM5_ESM.pdf]

Biostatistics Research Group,
Population Health Sciences Institute, Newcastle University

A randomised, double blind, controlled mechanistic study of rituximab and belimumab combination therapy in PR3 ANCA-associated vasculitis (COMBIVAS)

Statistical Analysis Plan
SAP Version number: 1.0
SAP Date: 27/02/2023

This statistical analysis plan is based on protocol version V5.0 [20/07/2021]

ISRCTN Number:
EudraCT Number: 2017-004645-24
REC Reference: 18/EE/0275

Sponsor: Cambridge University Hospitals NHS Foundation Trust and University of Cambridge
Sponsor protocol number:

Funder: MRC
Funder reference number: MR/R502145/1
GSK support: Ref 206852

Authorised by:

Name	James Wason	Role	Senior Statistician
Signature	[signature]	Date	27/02/2023
Name	Rachel Jones	Role	Chief Investigator
Signature	[signature]	Date	27/2/2023

This statistical analysis plan (SAP) provides a framework and guidelines for the statistical analysis and reporting of the *COMBIVAS* trial.

The SAP applies to a clean and validated dataset.

Any deviation from the methods outlined in this SAP will be documented in the statistical end of trial report. Example Tables, Figures and Listings are for illustrative purposes only and are subject to change.

This SAP, along with all other documents relating to the analysis of this trial, will be stored in the 'Statistical Section' of the Trial Master File (TMF) held and maintained by the PHSI Biostatistics Research Group. The final signed SAP will also be stored in section 16 of the main TMF (16. Statistics / 16.1 Final signed Statistical Analysis Plan).

Revision history

Version	Date	Changes made	Justification for change	Timing of change
V1.0	27/02/2022	First version	NA	

Abbreviations

AAV:	ANCA-associated vasculitis
AESIs:	AEs of special interest
BLyS:	B lymphocyte stimulator
BVAS/WG:	Birmingham vasculitis activity score / Wegener's Granulomatosis
CUH:	Cambridge University Hospitals
DSMB:	Data and Safety Monitoring Board
eGFR:	Estimated glomerular filtration rate
ELISA:	Enzyme-linked immunosorbent assay
GBM:	Glomerular basement membrane
IgG:	Immunoglobulin G
IM:	Intramuscular
IV:	Intravenous
MPO ANCA:	Myeloperoxidase anti-neutrophil cytoplasmic antibodies
PR3 ANCA:	Proteinase 3 anti-neutrophil cytoplasmic antibodies
VDI:	Vasculitis Damage Index
WBC:	White blood cell

Table of Contents

1. INTRODUCTION 5
1.1 BACKGROUND AND RATIONALE 5
1.2 OBJECTIVES 5

2. STUDY METHODS 6
2.1 TRIAL DESIGN 6
2.2 STUDY SETTING AND PATIENT POPULATION 6
2.3 RANDOMISATION AND BLINDING 7
2.4 DEFINITION OF OUTCOME MEASURES 7
2.4.1 PRIMARY ENDPOINT 7
2.4.2 SECONDARY ENDPOINTS 8
2.4.3 EXPLORATORY ENDPOINTS 10
2.5 STUDY ASSESSMENTS 11
2.6 SAMPLE SIZE AND POWER 13

3. STATISTICAL CONSIDERATIONS 15
3.1 TIMING OF ANALYSES 15
3.2 INTERIM ANALYSES, DATA MONITORING AND STOPPING GUIDELINES 15
3.3 ANALYSIS POPULATIONS 15

4. STUDY POPULATION 17
4.1 PARTICIPANT FLOW THROUGH TRIAL 17
4.2 FOLLOW-UP 18
4.3 BASELINE CHARACTERISTICS 18
4.4 TREATMENT COMPLIANCE 22

5. ANALYSIS METHODS 23
5.1 ANALYSIS OF PRIMARY OUTCOME 23
5.2 ANALYSIS OF SECONDARY OUTCOMES 24
5.3 ADDITIONAL / EXPLORATORY ANALYSES 51

6. SAFETY 54
6.1 ADVERSE EVENTS 55
6.2 SERIOUS ADVERSE EVENTS 56

7. STATISTICAL SOFTWARE 56

REFERENCES 57

APPENDIX 58

1. INTRODUCTION

1.1 Background and rationale

B lymphocyte stimulator (BLyS) is a B cell survival cytokine for which there is accumulating evidence supporting its role in the pathogenesis of ANCA-associated vasculitis (AAV). Elevated levels of circulating BLyS have been demonstrated in patients with AAV [1-4], and experimental models have shown that ANCA can activate neutrophils which release BLyS and promote increased survival of a B cell line in vitro [7]. Thus, ANCA-activated neutrophils arriving into inflammatory sites may directly promote the survival of B cells and may explain the elevated levels of BLyS observed in AAV.

Belimumab is human monoclonal antibody which binds soluble BLyS with high affinity and inhibits its biological activity. This results in apoptotic B cell death and reduction in circulating B cell numbers. In vivo, neutralisation of BLyS blocks the differentiation of transitional B cells to naïve B cells and the survival of these B cell subsets, thus reducing the number of B cells in the mature resting stage.

Rituximab is a chimeric mouse/human monoclonal antibody against CD20, resulting in depletion of B cells and is used in specific hematopoietic malignancies and in autoimmune disease such as rheumatoid arthritis and ANCA associated vasculitis.

1.2 Objectives

1.2.1 Primary objective

To compare the efficacy of belimumab (versus placebo) in combination with a single cycle of rituximab and corticosteroids in achieving proteinase 3 anti-neutrophil cytoplasmic antibodies (PR3 ANCA) negativity in participants with active AAV.

1.2.2 Secondary objectives

Efficacy (Major): To assess changes in PR3 ANCA following belimumab (or placebo) in combination with a single cycle of rituximab and corticosteroids in patients with active AAV.

Pharmacodynamic: To assess the changes in key white blood cell (WBC) populations and B and T cell subsets in blood during B cell depletion and B cell reconstitution.

Clinical: To compare the clinical efficacy of belimumab (versus placebo) in combination with a single cycle of rituximab and corticosteroids in participants with active AAV.

Safety: To compare the safety and tolerability of belimumab (versus placebo) in combination with a single cycle of rituximab and corticosteroids in participants with active AAV.

2. STUDY METHODS

2.1 Trial design

COMBIVAS is a multicentre, randomised, double blind, placebo-controlled mechanistic study. The trial population will be adult participants (at least 18 years of age) with active disease, who will be randomised to one of two treatment groups in a 1:1 ratio and receive rituximab plus prednisolone combined with either belimumab (combination arm) or placebo (control arm).

2.2 Study setting and patient population

This multi-centre trial includes 7 UK trial sites. The following is a summary of the eligibility criteria and the key exclusion criteria for participants in this trial:

Summary of eligibility criteria

- Participants must be ≥18 of age
- Have a diagnosis of AAV (granulomatosis with polyangiitis or microscopic polyangiitis)
- Have PR3 ANCA positivity by ELISA at screening
- Have active disease defined by one major or three minor disease activity items on BVAS/WG
- Be capable of giving signed informed consent

Key exclusion criteria:

- MPO ANCA or anti–GBM antibody positivity by ELISA at screening
- Presence of pulmonary haemorrhage with hypoxia at screening
- Estimated glomerular filtration rate (eGFR) <15 ml/min/1.73m2 at screening
- Have an acute serious or chronic infection at screening
- Have received any B cell targeted therapy within 180 days of Day 1
- Have received >3 infusions of cyclophosphamide within 90 days of Day 1
- Have received any steroid injection (e.g., intramuscular [IM], intraarticular, or IV) within 60 days of Day 1 (unless given during or 30 days before screening period)
- Have received emergency steroid >3g methylprednisolone (IV) or equivalent dose of oral prednisolone between 30 days prior to Screening Visit and Day 1 (including Day 1).
- Have undetectable peripheral blood B cells at screening
- Have IgG <400mg/dl at screening

For the full details of the eligibility and exclusion criteria please see protocol V5.0 20/07/2021.

2.3 Randomisation and blinding

Up to 40 participants will be randomised such that approximately 30 participants are evaluable.

Randomisation of participants will be stratified by screening PR3 ANCA levels (low vs. high by historical median value of 24). A randomisation schedule will be generated such that within each stratum, participants will be randomised to one of the two following treatment assignments in a 1:1 ratio using an online randomisation system accessible via password-protected access. Subjects will be randomly assigned to receive Belimumab drug product or matching placebo in a double-blind fashion. Thus, neither the investigator, nor the subject will know which study treatment is being administered.

The Data and Safety Monitoring Board (DSMB) will be unblinded and will perform safety reviews of adverse events and laboratory data during the conduct of the trial. It will not be possible for the study team to determine treatment allocation using the laboratory data generated during the trial period. Results of laboratory data with the potential to unblind trial staff (e.g. serum BLyS) will be measured on frozen samples and results provided when unblinding occurs.

2.4 Definition of outcome measures

2.4.1 Primary endpoint

Time to PR3 ANCA negativity (<2 iU/L) as measured at baseline, Weeks 4, 8, 12, 16, 20, 24, 28, 36, 44, 52 and Months 15, 18, 21, 24.

PR3 ANCA is assessed using the Phadia Assay on frozen samples analysed in batched. This is the same assay as the Cambridge University Hospitals (CUH) in-house assay used in routine clinical care. To minimise the potential impact on the primary endpoint analysis caused by missed samples that were not collected at the start of the COVID-19 pandemic, we plan to combine the ANCA values from trial samples with ANCA values collected as part of routine care. Experiments to validate the comparability between the two datasets are in progress.

Samples for which the reported ANCA value is >177 iU/L will be diluted in University of Cambridge Laboratories to allow the calculation of the actual value. ANCA negativity will be defined based on the reference ranges provided by the manufacturer (<2.0 iU/L). However, recognising the cut-off value for negativity is arbitrary, thresholds of <5.0 iU/L and <10 iU/L will also be examined as secondary endpoints. Where a planned

measurement is missing, the previous measurement will be carried over (Last Observation Carried Forward), except for endpoints requiring two or more consecutive measurements (e.g. sustained PR3 ANCA negativity).

2.4.2 Secondary endpoints

Efficacy (Major)

- Proportion of participants with PR3 ANCA negativity (ELISA) at Months 3 (W12) ,6 (W24), 12 (W52), 18 and 24.
- Proportion of participants with sustained PR3 ANCA negativity at Month 12 and Month 24.
- Percentage change in PR3 ANCA level from baseline to Months 3 (W12), 6 (W24), 12 (W52), 18 and 24.
- Time to PR3 ANCA <5 iU/L
- Time to PR3 ANCA <10 iU/L
- Time to 50% fall in PR3 ANCA (from baseline)
- Time to rise in PR3 ANCA (a rise constitutes a relative increase of at least 25% from the lowest measured level and an absolute increase of at least 10 iU/L)

Sustained PR3 ANCA negativity is defined as continued measurements of <2iU/L after the first negative value (<2iU/L) has been achieved.

Pharmacodynamic

- Absolute and percentage change from baseline (Day 1) in CD4 and CD8 T cells, B cells (CD19) and Natural Killer cells in blood at Months 3 (W12), 12 (W52), 18 and 24.
- Percentage change from baseline and absolute change from baseline in B cell subsets- naïve, transitional, memory, activated and plasmablast subsets in blood at Months 3 (W12), 12 (W52), 18 and 24.

Analysis will exclude patients with missing samples.

Table 2.4.1: Flow cytometric surface markers used to define cell types (validated assay used in GSK CUC laboratory)

Cell type	Definition
B cells	CD19+
B cells (CD20+)	CD19+CD20+
Naïve B cells	CD19+IgD+CD27-
Transitional B cells	CD19+CD24+CD38+
Memory B cells	CD19+CD27+
Activated memory B cells CD69+	CD19+CD27+CD69+

Activated memory B cells CD95+	CD19+CD27+CD95+
Switched memory B cells	CD19+IgD-CD27+
Activated switched memory B cells CD69+	CD19+IgD-CD27+CD69+
Activated switched memory B cells CD95+	CD19+IgD-CD27+CD95+
Non-switched memory B cells	CD19+IgD+CD27+
Activated non-switched memory B cells CD69+	CD19+IgD+CD27+CD69+
Activated non-switched memory B cells CD95+	CD19+IgD+CD27+CD95+
Double negative B cells	CD19+IgD-CD27-
Activated double negative B cells CD69+	CD19+IgD-CD27-CD69+
Activated double negative B cells CD95+	CD19+IgD-CD27-CD95+
Plasmablasts	CD19+CD27+CD38+
Activated plasmablasts CD69+	CD19+CD27+CD38+CD69+
Activated plasmablasts CD95+	CD19+CD27+CD38+CD95+

Efficacy (Clinical)

- Time to clinical remission (as measured by BVAS/WG at baseline, Weeks 4, 8, 12, 16, 20, 24, 36, 52 and Months 15, 18, 21, 24)
- Proportion of participants in complete remission at Months 6 (W24), 12 (W52) and 24 (W104)
- Time to first relapse (major or minor), as measured by BVAS/WG at baseline, Weeks 4, 8, 12, 16, 20, 24, 36, 52 and Months 15, 18, 21, 24 and at unscheduled (relapse) visits or prohibited medication in those who have achieved remission.
- Time to first relapse (major only, as measured by BVAS/WG at baseline, Weeks 4, 8, 12, 16, 20, 24, 36, 52 and Months 15, 18, 21, 24 and at unscheduled (relapse) visits or prohibited medication in those who have achieved remission.
- Proportion of participants with progressive disease before remission (major or minor).
- Proportion of participants with progressive disease before remission (major only).

Table 2.4.2: Clinical definitions

Minor progressive disease	Worsening disease activity with new/worse minor BVAS item(s) before remission is achieved.
Major progressive disease	Worsening disease activity with new/worse major BVAS item(s) before remission is achieved.
Remission	BVAS/WG≤1 (one minor persistent BVAS/WG item) confirmed by a consecutive assessment at least 30 days apart, and corticosteroid dose <10mg/day. Remission start dates from the first BVAS/WG≤1

Complete Remission	BVAS/WG = 0 confirmed by a consecutive assessment at least 30 days apart, and corticosteroid dose <10mg/day. Remission start dates from the first BVAS/WG=0
Relapse	The occurrence of any new BVAS/WG item after remission
Minor Relapse	Any increase in disease activity after remission that does not meet the definition of Major Relapse
Major Relapse	The development of a new or recurrent major disease activity item after remission using the BVAS/WG assessment tool

Safety

- Incidence of SAEs
- Incidence and severity of AEs of special interest (AESIs): all infections requiring antimicrobial, antiviral or antifungal treatment, hypogammaglobulinaemia (IgG <400 mg/dL (Grade 3) and <250 mg/dL (Grade 4)), systemic infusion/injection reactions, hypersensitivity reactions, malignancy, psychiatric events (including suicidality), severe skin reactions (including Toxic Epidermal Necrolysis and Stevens-Johnson syndrome), cardiac disorders (including angina, myocardial infarction, arrhythmia, heart failure),thromboembolic events, PRES, pregnancy.

2.4.3 Exploratory endpoints

The exploratory endpoints that are analysed by the Trial Statistician and therefore covered in this SAP are highlighted in bold. All other exploratory endpoints are analysed elsewhere with oversight by Mark McClure and James Wason.

Exploratory assessments of mechanism may include but are not limited to:

- Change from baseline (day biopsy samples were taken which was on or before day 1) in the proportions of various subsets of B cells and T cells from lymph node and nasal biopsy and blood samples at Months 3, 12, 18, and 24.

Flow cytometric surface markers used to define cell types (exploratory panel used in University of Cambridge Laboratories)

Cell type	Definition
B cells	CD19+
B cells (CD20+)	CD19+CD20+
Naïve B cells	CD19+IgD+CD27-
Transitional B cells	CD19+CD24+CD38+

Switched memory B cells	CD19+IgD-CD27+
Non-switched memory B cells	CD19+IgD+CD27+
Double negative B cells	CD19+IgD-CD27-
Plasmablasts	CD19+IgD-CD27+CD20-CD38+
Markers of activation	CD95, CD69
T cells	CD3+
CD4+ T cells	CD4+
Naïve T cells	CD4+CD45RA+CCR7+
Effector memory T cells	CD4+CD45RA-CCR7-
Central memory T cells	CD4+CD45RA-CCR7+
TEMRA cells	CD4+CD45RA+CCR7-
TH1 cells	CD4+CXCR3+CCR6-
Th17 cells	CD4+CXCR3-CCR6+
Th2 cells	CD4+CXCR3-CCR6-
T regulatory cells	CD4+CD127loCD25hi
TfH cells	CD4+CXCR5+PD1hi
Markers of activation	HLA-DR, CD38, CD69, CD25

- Single cell transcriptomic analysis of immune and non-immune cells from blood, lymph node and nasal tissue at baseline (Day 1) and Month 3 (W12)
- BCR clonality analysis in blood at baseline, Month 12 and Month 24
- Change in BLyS levels and cytokine/chemokine arrays from baseline by visit.
- Transcriptomic analysis
- Functional B and T cell assays
- Proteomic assessment of urine
- Quantification of urinary lymphocytes
- **Change from baseline in AAV-PRO by visit** (measured at baseline (Day 1), Months 3 (W12), 6 (W24), 12 (W52), 18 and 24)
- **Change from baseline in Vasculitis Damage Index (VDI) by visit** (measured at baseline (Day 1), Months 6 (W24), 12 (W52), 18 and 24.

2.5 Study assessments

Table 2.5.1: Schedule of assessments

	Screening	Year 1 - Treatment Period	Year 2 - Follow-up	Unscheduled visit	Early Withdrawal

Eligibility assessment	*X*				End of Therapy visit	8 week post last dose
Informed consent	*X*					
Medical history	*X*					
Demographics	*X*					
AE/SAE review (to be recorded from point of informed consent)	*X*	*x*				
Inclusion and exclusion criteria	*X*					
Full physical examination including height and weight, vital signs	*X*					
12-lead ECG	*X*					
BVAS/WG	*X*	*x*	*x*	*x*	*x*	
C-SSRS Screening	*X*					
Neurological Assessment	*X*	*x*	*x*	*x*	*x*	*x*
Laboratory Tests	*X*					
PR3 ANCA*, MPO ANCA, anti-GBM antibody	*X*					
Serology (HIV antibody, hepatitis B surface antigen [HBsAg], hepatitis B core antibody [HbcAb] and hepatitis C virus antibody)	*X*					
TB Quantiferon (or equivalent)	*X*					
Immunoglobulins, Total CD19 count	*X*					
FBC (Hb, WCC with differential, platelets), urea, creatinine, eGFR, sodium, potassium, ALT (or AST), ALP, bilirubin, albumin, CRP, ESR, glucose Urinalysis	*x*					
INR and aPTT	*X*					
Follicle-stimulating hormone (FSH) and oestradiol (women of non-childbearing potential only)	*X*					
Serum human chorionic gonadotropin (hCG) pregnancy test (for women of childbearing potential)	*X*					

AE/SAE review			x	x	x	X
Visit to trial centre		x	x	x	x	X
Randomisation		x				
Training on use of prefilled syringe		x				
Dispense Belimumab / belimumab-placebo		x				
Rituximab (IV)		x				
Concomitant medication review		x	x	x	x	x
Dispense / review patient diaries		x				
Return patient injection diary		x				
AAV-PRO		x	x		x	
Vasculitis Damage Index (VDI)		x	x		x	
Symptom-driven physical exam		x	x	x	x	x
C-SSRS		x	x	x	x	x
Urine pregnancy test (WOCBP only)		x	x	x	x	
Urine dipstick, urine microscopy, UPCR		x	x	x	x	
Routine bloods		x	x	x	x	x
PR3 ANCA (research sample)		x	x	x	X	
Blood leukocyte analysis		x	x	x	X	
Transcriptomics (whole blood)		x	x	x	X	
Exploratory blood biomarkers		x	x	x	X	
Urine proteomics		x	x	x	X	
Urine lymphocytes		x	x	x	X	
BLyS, BLyS-Beli complex, cytokines		x	x	x	x	
Lymph node and nasal biopsy (if consented)		x		x		
Nasal swab (microbiome)		x		x		

2.6 Sample size and power

Since the trial is exploratory in nature the sample size is based only on feasibility. However, the sample size may be justified statistically, albeit by making assumptions regarding the treatment effect. Rituximab leads to falls in PR3 ANCA levels and clinical remission. Persistent PR3 ANCA positivity at 6 months is associated with higher relapse [10]. Using data from 24 PR3 AAV patients treated with rituximab at Addenbrooke's Hospital, rituximab

was associated with a median time to PR3 ANCA ELISA negativity of 9 months. Time to ANCA negativity is the primary endpoint for this trial. With a two-sided 5% type I error rate, 15 patients per arm results in 80% power to detect a difference when the rituximab-belimumab group has a median time to PR3 ANCA negativity of 3 months. This is calculated assuming a log-rank test using the following formula for a two-sided test with type I error rate α and power 1-β:

$$n = \frac{\left(Z_{\frac{\alpha}{2}} + Z_{\beta}\right)^2}{d \log^2 \lambda}$$

where $Z_{\alpha/2}$ and Z_{β} are the quantiles of the standard normal distribution, hazard ratio is λ, d is the probability a participant in either group will eventually have an event.

No sample size re-estimation is currently planned for this trial. However, prior to cessation of recruitment, the drop-out rate will be assessed and further participants may be recruited to ensure approximately 30 participants are evaluable.

Figure 2.6.1: Study flowchart

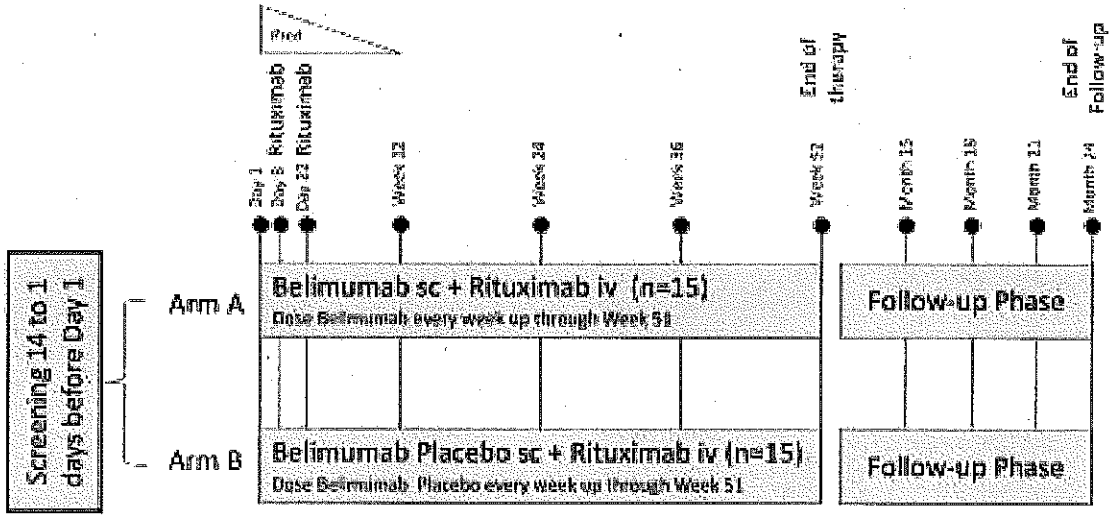

3. STATISTICAL CONSIDERATIONS

3.1 Timing of analyses

The final analysis will take place at the end of the trial after the data has been locked and all participants have stopped the study. The trial completed recruitment in March 2021 and follow will be complete by the March 2023 as the trial is 2 years + 4 weeks long (2 weeks screening and 2 weeks window on the final visit). Allowing 2 months for the final data cleaning and database lock then the analysis will start June 2023.

3.2 Interim analyses, data monitoring and stopping guidelines

There are no formal planned interim analyses. The DSMB will review unblinded safety data on an ongoing basis until all participants have completed the 2-year trial. The DSMB will meet approximately every 6 months. Ad hoc meetings of the DSMB can also be requested as needed to review urgent safety information or AEs. Events to be monitored during the safety review will include at a minimum: all SAEs (including deaths, serious psychiatric events, and serious infections), opportunistic infections (serious and non-serious), malignancies, and hypersensitivity/anaphylactic reactions during the double blind 52-week treatment and the 52-week follow-up phases of the trial.

3.3 Analysis populations

The following populations are defined:

Population	Description
Enrolled	All participants who sign the Informed Consent Form
Randomised	All participants who are randomised. This population will be comprised of participants according to<br>the treatment that a participant was randomised to receive, regardless of the actual treatment received.
Intention to Treat (ITT)	All randomised participants who receive at least one dose of belimumab / belimumab-placebo
Per Protocol (PP)	All randomised participants who receive at least one dose of rituximab and 3 months of belimumab/ belimumab-placebo and ≥ 8 injections in first 12 weeks.<br><br>No contraindicated medications (immunomodulatory).<br><br>Compliance with steroid taper (no more than 14 days of temporary increase in corticosteroids above the scheduled steroid taper during the first 3 months of trial). Beyond 3 months, average daily dose should be 0 +/- 10 mg/day and no more than one temporary increase in prednisolone for treatment of minor flare.<br><br>Excluding participants with major protocol deviations with potential to impact efficacy assessments.

	This population also defines 'evaluable' for sample size considerations.
Safety	All randomised participants who take at least 1 dose of trial treatment.<br><br>Participants will be analysed according to the treatment they actually received.

4. Study Population

Participant flow through the trial will be presented using a CONSORT diagram. Information will be provided on numbers and reasons for randomisation, patients discontinuing the intervention, patients not evaluable for the primary endpoints and withdrawal from follow-up.

4.1 Participant flow through trial

Figure 1: **CONSORT Flow Diagram**

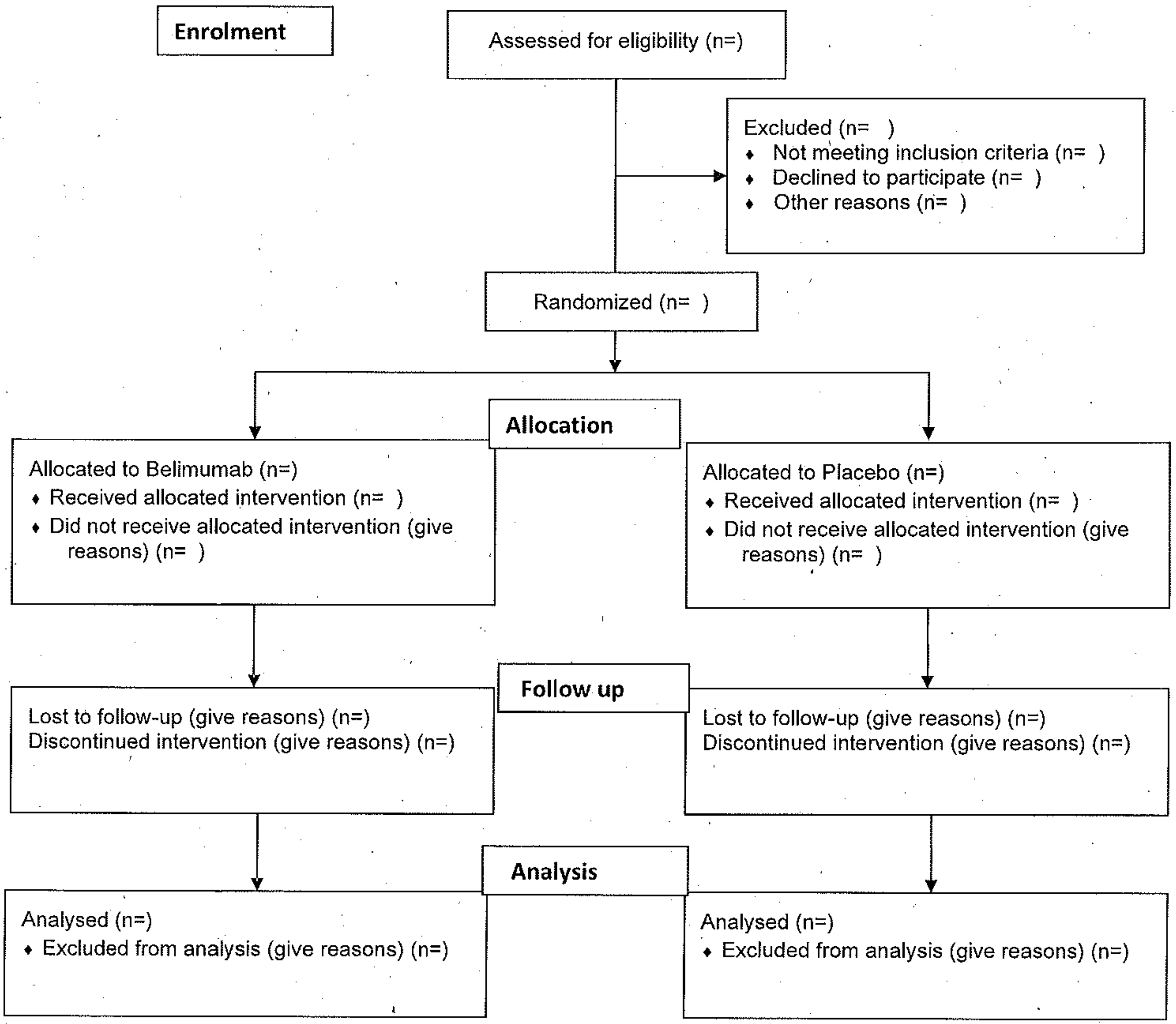

4.2 Follow-Up

The number of patients lost to follow up and/or withdrawn or died, with reasons given, will be reported. Follow-up will be compared across treatment conditions based on the number and percentage of participants who completed each scheduled data collection timepoint of the trial.

The number of patients lost to follow up and/or withdrawn or died, with reasons given, will be reported. Follow-up will be compared across treatment conditions based on the number and percentage of participants who completed each scheduled data collection timepoint of the trial.

4.3 Baseline characteristics

Descriptive statistics will be used to report the patient level baseline data by randomisation groups. This will consist of data and measures reported in or derived from data in the screening and baseline visits as detailed in the tables below. Categorical data will be presented as frequencies and percentages, continuous data will be presented as mean (sd), median (IQR, range) or both as appropriate.

Table 4.3.1: Patient demographics at baseline

**Demographics**	**Belimumab**	**Placebo**	**Overall**
**Number**			
**Age at Randomisation, median (Range)**			
**Sex, x (%)**<br>**Female**<br>**Male**			
**Race, x (%)**<br>**White**<br>**Asian**			

Data are n; %, mean (SD) or median (IQR); range, unless otherwise state

Table 4.3.2: Medical history (comorbidities)

**Demographics**	**Belimumab**	**Placebo**	**Overall**
**Number**			
**Relapsing/ newly diagnosed*, number (%)**<br>Newly Diagnosed<br>Relapsing			

** If the patient was enrolled within 3 months from the demographic CRF date they would be classed as newly diagnosed but if longer than 3 months then they would be relapsing.*

Table 4.3.3: Medication history

Medication history – has the subject ever received any of the following as treatment for their vasculitis?	**Belimumab**	**Placebo**	**Overall**
N			

Methylprednisolone 4 weeks prior to screening and during screening, x (%)<br>No<br>Yes<br>If Yes, cumulative dose, median (range)<br>N=			
Prednisolone dose prior to Day 1, mg per day<br>Nb: data extracted from conmeds form			
Oral Cyclophosphamide ever, x (%)<br>No<br>Yes<br>If Yes, cumulative dose, median g (range)<br>N=			
IV Cyclophosphamide ever, x (%)<br>No<br>Yes<br>If Yes, cumulative dose, median g (range)<br>N=			
IV Cyclophosphamide within 3 months of trial entry<br>No<br>Yes<br>If Yes, cumulative dose, median g (range)<br>N=			
Rituximab ever (last dose more than 6 months from screening), x (%)<br>No<br>Yes<br>If Yes, cumulative dose, median mg (range)<br>N=			
Azathioprine, x (%)<br>No<br>Yes			
Methotrexate, x (%)<br>No<br>Yes			
Mycophenolate Mofetil, x (%)<br>No<br>Yes			
Sulfamethoxazole/Trimethoprim, x (%)<br>No<br>Yes			
Plasma Exchange, x (%)<br>No<br>Yes			
IVIg, x (%)<br>No<br>Yes			
Anti-TNFs, x (%)<br>No			

Yes			
Other immunosuppression, x (%)<br>No<br>Yes			

Methylprednisolone ever, x (%)<br>No<br>Yes<br>If Yes, cumulative dose, median (range)			
Prednisolone ever, x (%)<br>Yes<br>If Yes, cumulative dose, median (range)			
Oral Cyclophosphamide ever, x (%)<br>No<br>Yes			
IV Cyclophosphamide ever, x (%)<br>No<br>Yes<br>If Yes, cumulative dose, median (range)			
Rituximab ever, x (%)<br>No<br>Yes<br>If Yes, cumulative dose, median (range)			
Azathioprine ever, x (%)<br>No<br>Yes			
Methotrexate ever, x (%)<br>No<br>Yes			
Mycophenolate Mofetil ever, x (%)<br>No<br>Yes			
Sulfaethoxazole/Trimethoprim ever, x (%)<br>No<br>Yes			
Plasma Exchange ever, x (%)<br>No<br>Yes			
IVIg ever, x (%)<br>No<br>Yes			
Anti-TNFs ever, x (%)<br>No<br>Yes			
Other immunosuppression ever, x (%)<br>No<br>Yes			

Table 4.3.4: Haematology data at screening and day 1

		Belimumab		Placebo		Overall	
	N						
Screening	Haemoglobin, median (range) n						
	Platelets, median (range) n						
	WBC, median (range) n						
	Neutrophils, median (range) n						
	Lymphocytes, median (range) n						
	ESR, median (range) n						
	CD19, median (range) n						
	INR, median (range) n						
	aPTT, median (range) n						
Day 1	Haemoglobin, median (range) n						
	Platelets, median (range) n						
	WBC, median (range) n						
	Neutrophils, median (range) n						
	Lymphocytes, median (range) n						
	ESR, median (range) n						
	CD19, median (range) n						
	INR, median (range) n						
	aPTT, median (range) n						

Table 4.3.5: Chemistry Lab data at screening and day 1

		Belimumab		Placebo		Overall	
	N						
Screening	Creatinine, median (range) n						
	eGFR, median (range) n						
	Urea, median (range) n						
	Sodium, median (range) n						
	Potassium, median (range) n						
	Glucose, median (range) n						
	ALT, median (range) n						
	AST, median (range) n						
	ALP, median (range) n						
	Total Bilirubin, median (range) n						
	Albumin, median (range) n						
	CRP, median (range) n						
	FSH, median (range) n						
	Oestradiol						
	Serum pregnancy test result, x (%) n<br>Negative						
	IgG, median (range) n						
	IgM, median (range) n						
	IgA, median (range) n						
	Anti-PR3, x (%) n<br>Positive						
	Anti-MPO, x (%) n<br>Negative						
	Anti-GBM, x (%) n<br>Negative						
Day 1	Creatinine, median (range) n						

	**eGFR, median (range) n**						
	**Urea, median (range) n**						
	**Sodium, median (range) n**						
	**Potassium, median (range) n**						
	**Glucose, median (range) n**						
	**ALT, median (range) n**						
	**AST, median (range) n**						
	**ALP, median (range) n**						
	**Total Bilirubin, median (range) n**						
	**Albumin, median (range) n**						
	**CRP, median (range) n**						
	**FSH, median (range) n**						
	**Oestradiol, median (range) n**						
	**Serum pregnancy test result, n(%)**						
	**IgG, median (range) n**						
	**IgM, median (range) n**						
	**IgA, median (range) n**						
	**Anti-PR3, x (%) n**						
	**Anti-MPO , x (%) n**						
	**Anti-GBM, x (%) n**						

Table 4.5.6: Urinology at screening and day 1

		**Belimumab**		**Placebo**		**Overall**	
	**N**						
**Screening**	**Protein, x (%) n**<br>**Negative**<br>**+**						
	**Blood, x (%) n**<br>**Negative**<br>**Trace**<br>**+**						
	**RBC count by microscopy**						
	**Urinary Creatinine**						
	**uPCR**						
**Day 1**	**Protein, x (%) n**<br>**Negative**<br>**+**						
	**Blood, x (%) n**<br>**Negative**<br>**Trace**<br>**+**						
	**RBC count by microscopy**						
	**Urinary Creatinine**						
	**uPCR**						

4.4 Treatment compliance

A record of the number of prefilled syringes dispensed and used by each participant must be maintained and reconciled with trial treatment and compliance records. Treatment start and stop dates, including dates for treatment delays will also be recorded in the CRF. We will summarise treatment compliance descriptively.

Table 4.4.1: Summary of allocated treatment received

	**Belimumab (n=)**		**Placebo (n=)**	
Received at least one dose of allocated treatment				
Completed treatment				
Discontinued treatment prematurely<br>*If Yes, due to*<br>*AE*<br>*Patient choice*<br>*Other**				
Median duration of treatment (days)				
Median dose intensity (%)				

*Other reasons will be provided in a line listing

Data are n; % or median (IQR); range, unless otherwise stated

5. ANALYSIS METHODS

Unless otherwise stated, all analyses will be conducted in the PP population.

5.1 Analysis of primary outcome

The time to PR3 ANCA negativity will be compared between Belimumab and Placebo using the Cox proportional hazard model with the Exact method for handling any ties and with binary covariates for screening PR3 ANCA levels (low vs. high by historic median value of 24) and treatment group (i.e., belimumab vs. placebo). If a patient becomes PR3 ANCA negative between two timepoints, the event time will be included as an interval censored observation between those two times.

Because the Cox proportional hazards model relies on the hazards to be proportional, we will test for this with Schoenfeld residuals.

Kaplain-Meier plots and median time to PR3 ANCA negativity will be presented for each randomised treatment group, along with the estimated hazard ratio of the treatments, its 95% confidence intervals and associated p-value. P-values will be based on the likelihood ratio test and CIs will use a profile likelihood approach. A HR less than 1 favours Belimumab. This analysis will be in the PP population.

Sensitivity Analysis: The primary outcome analysis will be repeated in the ITT population.

Table 5.1.1: Time to PR3 ANCA negativity (<2.0 iU/L) modelled using a Cox proportional hazards model adjusted for screening PR3 ANCA (<=24 or >24)

Outcome: time to PR3 ANCA negativity		Median time to PR3 ANCA negativity	Number of events N/patients; (%)	Comparison to Placebo	
				HR (95% CI)	p-value
Treatment	Placebo			1.00 (-)	-
	Belimumab				
Screening PR3 ANCA	Low (PR3 ANCA<=24)			1.00 (-)	-
	High (PR3 ANCA>24)				

5.2 Analysis of secondary outcomes

The following analyses will be completed in the PP population.

Efficacy

The analysis of the proportion of participants with PR3 negativity (ELISA) at 3, 6, 12, 18 and 24 will be compared between randomisation groups using mixed-effects logistic regression adjusted for screening PR3 ANCA levels (<=24 or >24) and random intercept for participant. In all cases where mixed-effects logistic regression is used, if there is a problem with model fit we will analyse the time points separately using logistic regressions.

Table 5.2.1: Proportion of participants with PR3 negativity (ELISA) using mixed-effects logistic regression adjusted for screening PR3 ANCA (<=24 or >24)

Outcome: Proportion of participants with PR3 negativity (ELISA) at 3 months		Comparison to Placebo	
		OR (95% CI)	p-value
**Treatment**	Placebo	1.00 (-)	-
	Belimumab		
**Screening PR3 ANCA**	Low (PR3 ANCA<=24)	1.00 (-)	-
	High (PR3 ANCA>24)		
**6 months**			
		OR (95% CI)	p-value
**Treatment**	Placebo	1.00 (-)	-
	Belimumab		
**Screening PR3 ANCA***	Low (PR3 ANCA<=24)	1.00 (-)	-
	High (PR3 ANCA>24)		
**12 months**			
		OR (95% CI)	p-value
**Treatment**	Placebo	1.00 (-)	-
	Belimumab		
**Screening PR3 ANCA***	Low (PR3 ANCA<=24)	1.00 (-)	-
	High (PR3 ANCA>24)		
**18 months**			

		OR (95% CI)	p-value
**Treatment**	Placebo	1.00 (-)	-
	Belimumab		
**Screening PR3 ANCA***	Low (PR3 ANCA<=24)	1.00 (-)	-
	High (PR3 ANCA>24)		
**24 months**			
		**OR (95% CI)**	**p-value**
**Treatment**	Placebo	1.00 (-)	-
	Belimumab		
**Screening PR3 ANCA***	Low (PR3 ANCA<=24)	1.00 (-)	-
	High (PR3 ANCA>24)		

*Only reported if separate models fitted for each timepoint

The analysis of the proportion of participants with sustained PR3 negativity (ELISA) at 24 months will also be compared between randomisation groups using logistic regression adjusted for screening PR3 ANCA levels (<=24 or >24).

Table 5.2.2: Proportion of participants with sustained PR3 negativity* (ELISA) using mixed-effects logistic regression adjusted for screening PR3 ANCA (<=24 or >24)

**Outcome: Proportion of participants with sustained PR3 negativity (ELISA) at 24 months**		**Comparison to Placebo**	
**12 months**		**OR (95% CI)**	**p-value**
Treatment	Placebo	1.00 (-)	-
	Belimumab		
Screening PR3 ANCA	Low (PR3 ANCA<=24)	1.00 (-)	-
	High (PR3 ANCA>24)		
**24 months**		**OR (95% CI)**	**p-value**
Treatment	Placebo	1.00 (-)	-
	Belimumab		
Screening PR3 ANCA	Low (PR3 ANCA<=24)	1.00 (-)	-
	High (PR3 ANCA>24)		

*Sustained PR3 ANCA negativity is defined as continued measurements of <2iU/L after the first negative value (<2iU/L) has been achieved.

Change from baseline in PR3 ANCA will be analysed using a Wilcoxon Rank Sum test, with p-value reported. A Hodges-Lehmann method will be used to provide estimates of the median differences and non-parametric 95% confidence intervals at 3, 6, 12, 18 and 24 months.

Table 5.2.3: Percentage change from baseline in PR3 ANCA

**Percentage change from baseline in PR3 ANCA at 3 months**		**Wilcoxon Rank Sum test**	
		**Mean Scores**	**p-value**
Treatment	Placebo		-
	Belimumab		
		**Hodges-Lehmann**	
		**Median differences**	**95%CI**
Treatment	Placebo		
	Belimumab		
**Percentage change from baseline in PR3 ANCA at 6 months**		**Wilcoxon Rank Sum test**	
		**Mean Scores**	**p-value**
Treatment	Placebo		-
	Belimumab		
		**Hodges-Lehmann**	
		**Median differences**	**95%CI**
Treatment	Placebo		
	Belimumab		
**Percentage change from baseline in PR3 ANCA at 12 months**		**Wilcoxon Rank Sum test**	
		**Mean Scores**	**p-value**
Treatment	Placebo		-
	Belimumab		
		**Hodges-Lehmann**	
		**Median differences**	**95%CI**
Treatment	Placebo		
	Belimumab		
**Percentage change from baseline in PR3 ANCA at 18 months**		**Wilcoxon Rank Sum test**	
		**Mean Scores**	**p-value**
Treatment	Placebo		-
	Belimumab		
		**Hodges-Lehmann**	
		**Median differences**	**95%CI**
Treatment	Placebo		
	Belimumab		
**Percentage change from baseline in PR3 ANCA at 24 months**		**Wilcoxon Rank Sum test**	
		**Mean Scores**	**p-value**
Treatment	Placebo		-

	Belimumab		
		Hodges-Lehmann	
		Median differences	95%CI
Treatment	Placebo		
	Belimumab		

Subsequent endpoints will be analysed using the same approach as above, depending on the outcome type

Table 5.2.4: Time to PR3 ANCA <5 iU/L modelled using a Cox proportional hazards model adjusted for screening PR3 ANCA (<=24 or >24)

		Median time to PR3 ANCA <5 iU/L	Number of events n/patients; (%)	HR (95% CI)	p-value
Treatment	Placebo			1.00 (-)	-
	Belimumab				
Screening PR3 ANCA	Low (PR3 ANCA<=24)			1.00 (-)	-
	High (PR3 ANCA>24)				

Table 5.2.5: Time to PR3 ANCA <10 iU/L modelled using a Cox proportional hazards model adjusted for screening PR3 ANCA (<=24 or >24)

		Median time to PR3 ANCA <10 iU/L	Number of events n/patients; (%)	HR (95% CI)	p-value
Treatment	Placebo			1.00 (-)	-
	Belimumab				
Screening PR3 ANCA	Low (PR3 ANCA<=24)			1.00 (-)	-
	High (PR3 ANCA>24)				

Table 5.2.6: Time to 50% fall in PR3 ANCA from baseline modelled using a Cox proportional hazards model adjusted for screening PR3 ANCA (<=24 or >24)

		Median time to 50% fall in PR3 ANCA (from baseline)	Number of events n/patients; (%)	HR (95% CI)	p-value
Treatment	Placebo			1.00 (-)	-

	Belimumab				
Screening PR3 ANCA	Low (PR3 ANCA<=24)			1.00 (-)	-
	High (PR3 ANCA>24)				

Table 5.2.7: Time to rise in PR3 ANCA from nadir* modelled using a Cox proportional hazards model adjusted for screening PR3 ANCA (<=24 or >24)

		Median time to rise in PR3 ANCA*	Number of events n/patients; (%)	HR (95% CI)	p-value
Treatment	Placebo			1.00 (-)	-
	Belimumab				
Screening PR3 ANCA	Low (PR3 ANCA<=24)			1.00 (-)	-
	High (PR3 ANCA>24)				

***A rise constitutes a relative increase of at least 25% from the lowest measured level and an absolute increase of at least 10 iU/L**

Pharmacodynamic

The change from baseline in CD4 and CD8 T cells, B cells and NK cells in blood (3, 12, 24 months) and the change from baseline in naïve, transitional, memory, activated and plasmablast subsets in blood (3, 12, 24 months) will be analysed as a percentage change. The percent change from baseline data for will be compared between randomisation groups using a Wilcoxon Rank Sum test. A Hodges-Lehmann method will be used to provide estimates of the median differences and non-parametric 95% confidence intervals at 3, 12, and 24 months.

Table 5.2.8: Change from baseline in CD4 T cells

**Change from baseline in CD4 T cells at 3 months**		**Wilcoxon Rank Sum test**	
		**Mean Scores**	**p-value**
Treatment	Placebo		-
	Belimumab		
		**Hodges-Lehmann**	
		**Median differences**	**95%CI**
Treatment	Placebo		
	Belimumab		
**Change from baseline in CD4 T cells at 12 months**		**Wilcoxon Rank Sum test**	
		**Mean Scores**	**p-value**
Treatment	Placebo		-
	Belimumab		

		Hodges-Lehmann	
		**Median differences**	**95%CI**
Treatment	Placebo		
	Belimumab		
**Change from baseline in CD4 T cells at 18 months**		**Wilcoxon Rank Sum test**	
		**Mean Scores**	**p-value**
Treatment	Placebo		-
	Belimumab		
		**Hodges-Lehmann**	
		**Median differences**	**95%CI**
Treatment	Placebo		
	Belimumab		
**Change from baseline in CD4 T cells at 24 months**		**Wilcoxon Rank Sum test**	
		**Mean Scores**	**p-value**
Treatment	Placebo		-
	Belimumab		
		**Hodges-Lehmann**	
		**Median differences**	**95%CI**
Treatment	Placebo		
	Belimumab		

Table 5.2.9: Change from baseline in CD8 T cells

**Change from baseline in CD8 T cells at 3 months**		**Wilcoxon Rank Sum test**	
		**Mean Scores**	**p-value**
Treatment	Placebo		-
	Belimumab		
		**Hodges-Lehmann**	
		**Median differences**	**95%CI**
Treatment	Placebo		
	Belimumab		
**Change from baseline in CD8 T cells at 12 months**		**Wilcoxon Rank Sum test**	
		**Mean Scores**	**p-value**
Treatment	Placebo		-
	Belimumab		
		**Hodges-Lehmann**	
		**Median differences**	**95%CI**
Treatment	Placebo		

	Belimumab		
Change from baseline in CD8 T cells at 18 months		Wilcoxon Rank Sum test	
		Mean Scores	p-value
Treatment	Placebo		-
	Belimumab		
		Hodges-Lehmann	
		Median differences	95%CI
Treatment	Placebo		
	Belimumab		
Change from baseline in CD8 T cells at 24 months		Wilcoxon Rank Sum test	
		Mean Scores	p-value
Treatment	Placebo		-
	Belimumab		
		Hodges-Lehmann	
		Median differences	95%CI
Treatment	Placebo		
	Belimumab		

Table 5.2.10: Change from baseline in B cells (see Table 2.4.1 for definition)

Change from baseline in B cells at 3 months		Wilcoxon Rank Sum test	
		Mean Scores	p-value
Treatment	Placebo		-
	Belimumab		
		Hodges-Lehmann	
		Median differences	95%CI
Treatment	Placebo		
	Belimumab		
Change from baseline in B cells at 12 months		Wilcoxon Rank Sum test	
		Mean Scores	p-value
Treatment	Placebo		-
	Belimumab		
		Hodges-Lehmann	
		Median differences	95%CI
Treatment	Placebo		
	Belimumab		
Change from baseline in B cells at 18 months		Wilcoxon Rank Sum test	
		Mean Scores	p-value

Treatment	Placebo		-
	Belimumab		
		Hodges-Lehmann	
		Median differences	95%CI
Treatment	Placebo		
	Belimumab		
Change from baseline in B cells at 24 months		Wilcoxon Rank Sum test	
		Mean Scores	p-value
Treatment	Placebo		-
	Belimumab		
		Hodges-Lehmann	
		Median differences	95%CI
Treatment	Placebo		
	Belimumab		

Table 5.2.11: Change from baseline in B cells (CD20+) (see Table 2.4.1 for definition)

Change from baseline in B cells (CD20+) at 3 months		Wilcoxon Rank Sum test	
		Mean Scores	p-value
Treatment	Placebo		-
	Belimumab		
		Hodges-Lehmann	
		Median differences	95%CI
Treatment	Placebo		
	Belimumab		
Change from baseline in B cells (CD20+) at 12 months		Wilcoxon Rank Sum test	
		Mean Scores	p-value
Treatment	Placebo		-
	Belimumab		
		Hodges-Lehmann	
		Median differences	95%CI
Treatment	Placebo		
	Belimumab		
Change from baseline in B cells (CD20+) at 18 months		Wilcoxon Rank Sum test	
		Mean Scores	p-value
Treatment	Placebo		-
	Belimumab		
		Hodges-Lehmann	

		**Median differences**	**95%CI**
Treatment	Placebo		
	Belimumab		
**Change from baseline in B cells (CD20+) at 24 months**		**Wilcoxon Rank Sum test**	
		**Mean Scores**	**p-value**
Treatment	Placebo		-
	Belimumab		
		**Hodges-Lehmann**	
		**Median differences**	**95%CI**
Treatment	Placebo		
	Belimumab		

Table 5.2.12: Change from baseline in NK cells

**Change from baseline in NK cells at 3 months**		**Wilcoxon Rank Sum test**	
		**Mean Scores**	**p-value**
Treatment	Placebo		-
	Belimumab		
		**Hodges-Lehmann**	
		**Median differences**	**95%CI**
Treatment	Placebo		
	Belimumab		
**Change from baseline in NK cells at 12 months**		**Wilcoxon Rank Sum test**	
		**Mean Scores**	**p-value**
Treatment	Placebo		-
	Belimumab		
		**Hodges-Lehmann**	
		**Median differences**	**95%CI**
Treatment	Placebo		
	Belimumab		
**Change from baseline in NK cells at 18 months**		**Wilcoxon Rank Sum test**	
		**Mean Scores**	**p-value**
Treatment	Placebo		-
	Belimumab		
		**Hodges-Lehmann**	
		**Median differences**	**95%CI**
Treatment	Placebo		

	Belimumab		
Change from baseline in NK cells at 24 months		Wilcoxon Rank Sum test	
		Mean Scores	p-value
Treatment	Placebo		-
	Belimumab		
		Hodges-Lehmann	
		Median differences	95%CI
Treatment	Placebo		
	Belimumab		

Table 5.2.13: Change from baseline in naïve B cells (see Table 2.4.1 for definition)

Change from baseline in naïve B cells at 3 months		Wilcoxon Rank Sum test	
		Mean Scores	p-value
Treatment	Placebo		-
	Belimumab		
		Hodges-Lehmann	
		Median differences	95%CI
Treatment	Placebo		
	Belimumab		
Change from baseline in naïve B cells at 12 months		Wilcoxon Rank Sum test	
		Mean Scores	p-value
Treatment	Placebo		-
	Belimumab		
		Hodges-Lehmann	
		Median differences	95%CI
Treatment	Placebo		
	Belimumab		
Change from baseline in naïve B cells at 18 months		Wilcoxon Rank Sum test	
		Mean Scores	p-value
Treatment	Placebo		-
	Belimumab		
		Hodges-Lehmann	
		Median differences	95%CI
Treatment	Placebo		
	Belimumab		
Change from baseline in naïve B cells at 24 months		Wilcoxon Rank Sum test	
		Mean Scores	p-value

Treatment	Placebo		-
	Belimumab		
		**Hodges-Lehmann**	
		**Median differences**	**95%CI**
Treatment	Placebo		
	Belimumab		

Table 5.2.14: Change from baseline in transitional B cells (see Table 2.4.1 for definition)

**Change from baseline in transitional B cells at 3 months**		**Wilcoxon Rank Sum test**	
		**Mean Scores**	**p-value**
Treatment	Placebo		-
	Belimumab		
		**Hodges-Lehmann**	
		**Median differences**	**95%CI**
Treatment	Placebo		
	Belimumab		
**Change from baseline in transitional B cells at 12 months**		**Wilcoxon Rank Sum test**	
		**Mean Scores**	**p-value**
Treatment	Placebo		-
	Belimumab		
		**Hodges-Lehmann**	
		**Median differences**	**95%CI**
Treatment	Placebo		
	Belimumab		
**Change from baseline in transitional B cells at 18 months**		**Wilcoxon Rank Sum test**	
		**Mean Scores**	**p-value**
Treatment	Placebo		-
	Belimumab		
		**Hodges-Lehmann**	
		**Median differences**	**95%CI**
Treatment	Placebo		
	Belimumab		
**Change from baseline in transitional B cells at 24 months**		**Wilcoxon Rank Sum test**	
		**Mean Scores**	**p-value**
Treatment	Placebo		-
	Belimumab		
		**Hodges-Lehmann**	

		Median differences	95%CI
Treatment	Placebo		
	Belimumab		

Table 5.2.15: Change from baseline in memory B cells (see Table 2.4.1 for definition)

**Change from baseline in memory B cells at 3 months**		**Wilcoxon Rank Sum test**	
		**Mean Scores**	**p-value**
Treatment	Placebo		-
	Belimumab		
		**Hodges-Lehmann**	
		**Median differences**	**95%CI**
Treatment	Placebo		
	Belimumab		
**Change from baseline in memory B cells at 12 months**		**Wilcoxon Rank Sum test**	
		**Mean Scores**	**p-value**
Treatment	Placebo		-
	Belimumab		
		**Hodges-Lehmann**	
		**Median differences**	**95%CI**
Treatment	Placebo		
	Belimumab		
**Change from baseline in memory B cells at 18 months**		**Wilcoxon Rank Sum test**	
		**Mean Scores**	**p-value**
Treatment	Placebo		-
	Belimumab		
		**Hodges-Lehmann**	
		**Median differences**	**95%CI**
Treatment	Placebo		
	Belimumab		
**Change from baseline in memory B cells at 24 months**		**Wilcoxon Rank Sum test**	
		**Mean Scores**	**p-value**
Treatment	Placebo		-
	Belimumab		
		**Hodges-Lehmann**	
		**Median differences**	**95%CI**
Treatment	Placebo		
	Belimumab		

Table 5.2.16: Change from baseline in activated memory B cells CD69+ (see Table 2.4.1 for definition)

**Change from baseline in activated memory B cells CD69+ at 3 months**		**Wilcoxon Rank Sum test**	
		**Mean Scores**	**p-value**
Treatment	Placebo		-
	Belimumab		
		**Hodges-Lehmann**	
		**Median differences**	**95%CI**
Treatment	Placebo		
	Belimumab		
**Change from baseline in activated memory B cells CD69+ at 12 months**		**Wilcoxon Rank Sum test**	
		**Mean Scores**	**p-value**
Treatment	Placebo		-
	Belimumab		
		**Hodges-Lehmann**	
		**Median differences**	**95%CI**
Treatment	Placebo		
	Belimumab		
**Change from baseline in activated memory B cells CD69+ at 18 months**		**Wilcoxon Rank Sum test**	
		**Mean Scores**	**p-value**
Treatment	Placebo		-
	Belimumab		
		**Hodges-Lehmann**	
		**Median differences**	**95%CI**
Treatment	Placebo		
	Belimumab		
**Change from baseline in activated memory B cells CD69+ at 24 months**		**Wilcoxon Rank Sum test**	
		**Mean Scores**	**p-value**
Treatment	Placebo		-
	Belimumab		
		**Hodges-Lehmann**	
		**Median differences**	**95%CI**
Treatment	Placebo		
	Belimumab		

Table 5.2.17: Change from baseline in memory B cells CD95+ (see Table 2.4.1 for definition)

**Change from baseline in in memory B cells CD95+ at 3 months**		**Wilcoxon Rank Sum test**	
		**Mean Scores**	**p-value**
Treatment	Placebo		-
	Belimumab		
		**Hodges-Lehmann**	
		**Median differences**	**95%CI**
Treatment	Placebo		
	Belimumab		
**Change from baseline in in memory B cells CD95+ at 12 months**		**Wilcoxon Rank Sum test**	
		**Mean Scores**	**p-value**
Treatment	Placebo		-
	Belimumab		
		**Hodges-Lehmann**	
		**Median differences**	**95%CI**
Treatment	Placebo		
	Belimumab		
**Change from baseline in in memory B cells CD95+ at 18 months**		**Wilcoxon Rank Sum test**	
		**Mean Scores**	**p-value**
Treatment	Placebo		-
	Belimumab		
		**Hodges-Lehmann**	
		**Median differences**	**95%CI**
Treatment	Placebo		
	Belimumab		
**Change from baseline in in memory B cells CD95+ at 24 months**		**Wilcoxon Rank Sum test**	
		**Mean Scores**	**p-value**
Treatment	Placebo		-
	Belimumab		
		**Hodges-Lehmann**	
		**Median differences**	**95%CI**
Treatment	Placebo		
	Belimumab		

Table 5.2.18: Change from baseline in switched memory B cells (see Table 2.4.1 for definition)

**Change from baseline in switched memory B cells at 3 months**		**Wilcoxon Rank Sum test**	
		**Mean Scores**	**p-value**
Treatment	Placebo		-
	Belimumab		
		**Hodges-Lehmann**	
		**Median differences**	**95%CI**
Treatment	Placebo		
	Belimumab		
**Change from baseline in switched memory B cells at 12 months**		**Wilcoxon Rank Sum test**	
		**Mean Scores**	**p-value**
Treatment	Placebo		-
	Belimumab		
		**Hodges-Lehmann**	
		**Median differences**	**95%CI**
Treatment	Placebo		
	Belimumab		
**Change from baseline in switched memory B cells at 18 months**		**Wilcoxon Rank Sum test**	
		**Mean Scores**	**p-value**
Treatment	Placebo		-
	Belimumab		
		**Hodges-Lehmann**	
		**Median differences**	**95%CI**
Treatment	Placebo		
	Belimumab		
**Change from baseline in switched memory B cells at 24 months**		**Wilcoxon Rank Sum test**	
		**Mean Scores**	**p-value**
Treatment	Placebo		-
	Belimumab		
		**Hodges-Lehmann**	
		**Median differences**	**95%CI**
Treatment	Placebo		
	Belimumab		

Table 5.2.19: Change from baseline in activated switched memory B cells CD69+ (see Table 2.4.1 for definition)

**Change from baseline in activated switched memory B cells CD69+ at 3 months**		**Wilcoxon Rank Sum test**	
		**Mean Scores**	**p-value**
Treatment	Placebo		-
	Belimumab		
		**Hodges-Lehmann**	
		**Median differences**	**95%CI**
Treatment	Placebo		
	Belimumab		

**Change from baseline in activated switched memory B cells CD69+ at 12 months**		**Wilcoxon Rank Sum test**	
		**Mean Scores**	**p-value**
Treatment	Placebo		-
	Belimumab		
		**Hodges-Lehmann**	
		**Median differences**	**95%CI**
Treatment	Placebo		
	Belimumab		

**Change from baseline in activated switched memory B cells CD69+ at 18 months**		**Wilcoxon Rank Sum test**	
		**Mean Scores**	**p-value**
Treatment	Placebo		-
	Belimumab		
		**Hodges-Lehmann**	
		**Median differences**	**95%CI**
Treatment	Placebo		
	Belimumab		

**Change from baseline in activated switched memory B cells CD69+ at 24 months**		**Wilcoxon Rank Sum test**	
		**Mean Scores**	**p-value**
Treatment	Placebo		-
	Belimumab		
		**Hodges-Lehmann**	
		**Median differences**	**95%CI**
Treatment	Placebo		
	Belimumab		

Table 5.2.20: Change from baseline in activated switched memory B cells CD95+ (see Table 2.4.1 for definition)

**Change from baseline in activated switched memory B cells CD95+ at 3 months**		**Wilcoxon Rank Sum test**	
		**Mean Scores**	**p-value**
Treatment	Placebo		-
	Belimumab		
		**Hodges-Lehmann**	
		**Median differences**	**95%CI**
Treatment	Placebo		
	Belimumab		
**Change from baseline in activated switched memory B cells CD95+ at 12 months**		**Wilcoxon Rank Sum test**	
		**Mean Scores**	**p-value**
Treatment	Placebo		-
	Belimumab		
		**Hodges-Lehmann**	
		**Median differences**	**95%CI**
Treatment	Placebo		
	Belimumab		
**Change from baseline in activated switched memory B cells CD95+ at 18 months**		**Wilcoxon Rank Sum test**	
		**Mean Scores**	**p-value**
Treatment	Placebo		-
	Belimumab		
		**Hodges-Lehmann**	
		**Median differences**	**95%CI**
Treatment	Placebo		
	Belimumab		
**Change from baseline in activated switched memory B cells CD95+ at 24 months**		**Wilcoxon Rank Sum test**	
		**Mean Scores**	**p-value**
Treatment	Placebo		-

	Belimumab		
		Hodges-Lehmann	
		Median differences	95%CI
Treatment	Placebo		
	Belimumab		

Table 5.2.21: Change from baseline in non-switched memory B cells (see Table 2.4.1 for definition)

**Change from baseline in non-switched memory B cells at 3 months**		**Wilcoxon Rank Sum test**	
		**Mean Scores**	**p-value**
Treatment	Placebo		-
	Belimumab		
		**Hodges-Lehmann**	
		**Median differences**	**95%CI**
Treatment	Placebo		
	Belimumab		
**Change from baseline in non-switched memory B cells at 12 months**		**Wilcoxon Rank Sum test**	
		**Mean Scores**	**p-value**
Treatment	Placebo		-
	Belimumab		
		**Hodges-Lehmann**	
		**Median differences**	**95%CI**
Treatment	Placebo		
	Belimumab		
**Change from baseline in non-switched memory B cells at 18 months**		**Wilcoxon Rank Sum test**	
		**Mean Scores**	**p-value**
Treatment	Placebo		-
	Belimumab		
		**Hodges-Lehmann**	
		**Median differences**	**95%CI**
Treatment	Placebo		
	Belimumab		
**Change from baseline in non-switched memory B cells at 24 months**		**Wilcoxon Rank Sum test**	
		**Mean Scores**	**p-value**
Treatment	Placebo		-
	Belimumab		
		**Hodges-Lehmann**	

		**Median differences**	**95%CI**
Treatment	Placebo		
	Belimumab		

Table 5.2.22: Change from baseline in activated non-switched memory B cells CD69+ (see Table 2.4.1 for definition)

**Change from baseline in activated non-switched memory B cells CD69+ at 3 months**		**Wilcoxon Rank Sum test**	
		**Mean Scores**	**p-value**
Treatment	Placebo		-
	Belimumab		
		**Hodges-Lehmann**	
		**Median differences**	**95%CI**
Treatment	Placebo		
	Belimumab		
**Change from baseline in activated non-switched memory B cells CD69+ at 12 months**		**Wilcoxon Rank Sum test**	
		**Mean Scores**	**p-value**
Treatment	Placebo		-
	Belimumab		
		**Hodges-Lehmann**	
		**Median differences**	**95%CI**
Treatment	Placebo		
	Belimumab		
**Change from baseline in activated non-switched memory B cells CD69+ at 18 months**		**Wilcoxon Rank Sum test**	
		**Mean Scores**	**p-value**
Treatment	Placebo		-
	Belimumab		
		**Hodges-Lehmann**	
		**Median differences**	**95%CI**
Treatment	Placebo		
	Belimumab		
**Change from baseline in activated non-switched memory B cells CD69+ at 24 months**		**Wilcoxon Rank Sum test**	

		Mean Scores	p-value
Treatment	Placebo		-
	Belimumab		
		Hodges-Lehmann	
		Median differences	95%CI
Treatment	Placebo		
	Belimumab		

Table 5.2.23 Change from baseline in activated non-switched memory B cells CD95+ (see Table 2.4.1 for definition)

Change from baseline in activated non-switched memory B cells CD95+ at 3 months		Wilcoxon Rank Sum test	
		Mean Scores	p-value
Treatment	Placebo		-
	Belimumab		
		Hodges-Lehmann	
		Median differences	95%CI
Treatment	Placebo		
	Belimumab		
Change from baseline in activated non-switched memory B cells CD95+ at 12 months		Wilcoxon Rank Sum test	
		Mean Scores	p-value
Treatment	Placebo		-
	Belimumab		
		Hodges-Lehmann	
		Median differences	95%CI
Treatment	Placebo		
	Belimumab		
Change from baseline in activated non-switched memory B cells CD95+ at 18 months		Wilcoxon Rank Sum test	
		Mean Scores	p-value
Treatment	Placebo		-
	Belimumab		
		Hodges-Lehmann	
		Median differences	95%CI

Treatment	Placebo		
	Belimumab		
**Change from baseline in activated non-switched memory B cells CD95+ at 24 months**		**Wilcoxon Rank Sum test**	
		**Mean Scores**	**p-value**
Treatment	Placebo		-
	Belimumab		
		**Hodges-Lehmann**	
		**Median differences**	**95%CI**
Treatment	Placebo		
	Belimumab		

Table 5.2.24: Change from baseline in double negative B cells (see Table 2.4.1 for definition)

**Change from baseline in double negative B cells at 3 months**		**Wilcoxon Rank Sum test**	
		**Mean Scores**	**p-value**
Treatment	Placebo		-
	Belimumab		
		**Hodges-Lehmann**	
		**Median differences**	**95%CI**
Treatment	Placebo		
	Belimumab		
**Change from baseline in double negative B cells at 12 months**		**Wilcoxon Rank Sum test**	
		**Mean Scores**	**p-value**
Treatment	Placebo		-
	Belimumab		
		**Hodges-Lehmann**	
		**Median differences**	**95%CI**
Treatment	Placebo		
	Belimumab		
**Change from baseline in double negative B cells at 18 months**		**Wilcoxon Rank Sum test**	
		**Mean Scores**	**p-value**
Treatment	Placebo		-
	Belimumab		
		**Hodges-Lehmann**	
		**Median differences**	**95%CI**
Treatment	Placebo		

	Belimumab		
Change from baseline in double negative B cells at 24 months		Wilcoxon Rank Sum test	
		Mean Scores	p-value
Treatment	Placebo		-
	Belimumab		
		Hodges-Lehmann	
		Median differences	95%CI
Treatment	Placebo		
	Belimumab		

Table 5.2.25: Change from baseline in activated double negative B cells CD69+ (see Table 2.4.1 for definition)

Change from baseline in activated double negative B cells CD69+ at 3 months		Wilcoxon Rank Sum test	
		Mean Scores	p-value
Treatment	Placebo		-
	Belimumab		
		Hodges-Lehmann	
		Median differences	95%CI
Treatment	Placebo		
	Belimumab		
Change from baseline in activated double negative B cells CD69+ at 12 months		Wilcoxon Rank Sum test	
		Mean Scores	p-value
Treatment	Placebo		-
	Belimumab		
		Hodges-Lehmann	
		Median differences	95%CI
Treatment	Placebo		
	Belimumab		
Change from baseline in activated double negative B cells CD69+ at 18 months		Wilcoxon Rank Sum test	
		Mean Scores	p-value
Treatment	Placebo		-
	Belimumab		
		Hodges-Lehmann	

		Median differences	95%CI
Treatment	Placebo		
	Belimumab		
**Change from baseline in activated double negative B cells CD69+ at 24 months**		**Wilcoxon Rank Sum test**	
		**Mean Scores**	**p-value**
Treatment	Placebo		-
	Belimumab		
		**Hodges-Lehmann**	
		**Median differences**	**95%CI**
Treatment	Placebo		
	Belimumab		

Table 5.2.26: Change from baseline in activated double negative B cells CD95+ (see Table 2.4.1 for definition)

**Change from baseline in activated double negative B cells CD95+ at 3 months**		**Wilcoxon Rank Sum test**	
		**Mean Scores**	**p-value**
Treatment	Placebo		-
	Belimumab		
		**Hodges-Lehmann**	
		**Median differences**	**95%CI**
Treatment	Placebo		
	Belimumab		
**Change from baseline in activated double negative B cells CD95+ at 12 months**		**Wilcoxon Rank Sum test**	
		**Mean Scores**	**p-value**
Treatment	Placebo		-
	Belimumab		
		**Hodges-Lehmann**	
		**Median differences**	**95%CI**
Treatment	Placebo		
	Belimumab		
**Change from baseline in activated double negative B cells CD95+ at 18 months**		**Wilcoxon Rank Sum test**	
		**Mean Scores**	**p-value**
Treatment	Placebo		-

	Belimumab		
		Hodges-Lehmann	
		Median differences	95%CI
Treatment	Placebo		
	Belimumab		
**Change from baseline in activated double negative B cells CD95+ at 24 months**		**Wilcoxon Rank Sum test**	
		**Mean Scores**	**p-value**
Treatment	Placebo		-
	Belimumab		
		**Hodges-Lehmann**	
		**Median differences**	**95%CI**
Treatment	Placebo		
	Belimumab		

Table 5.2.27: Change from baseline in plasmablasts (see Table 2.4.1 for definition)

**Change from baseline in plasmablasts at 3 months**		**Wilcoxon Rank Sum test**	
		**Mean Scores**	**p-value**
Treatment	Placebo		-
	Belimumab		
		**Hodges-Lehmann**	
		**Median differences**	**95%CI**
Treatment	Placebo		
	Belimumab		
**Change from baseline in plasmablasts at 12 months**		**Wilcoxon Rank Sum test**	
		**Mean Scores**	**p-value**
Treatment	Placebo		-
	Belimumab		
		**Hodges-Lehmann**	
		**Median differences**	**95%CI**
Treatment	Placebo		
	Belimumab		
**Change from baseline in plasmablasts at 18 months**		**Wilcoxon Rank Sum test**	
		**Mean Scores**	**p-value**
Treatment	Placebo		-
	Belimumab		

		Hodges-Lehmann	
		**Median differences**	**95%CI**
Treatment	Placebo		
	Belimumab		
**Change from baseline in plasmablasts at 24 months**		**Wilcoxon Rank Sum test**	
		**Mean Scores**	**p-value**
Treatment	Placebo		-
	Belimumab		
		**Hodges-Lehmann**	
		**Median differences**	**95%CI**
Treatment	Placebo		
	Belimumab		

Table 5.2.28: Change from baseline in activated plasmablasts CD69+ (see Table 2.4.1 for definition)

**Change from baseline in activated plasmablasts CD69+ at 3 months**		**Wilcoxon Rank Sum test**	
		**Mean Scores**	**p-value**
Treatment	Placebo		-
	Belimumab		
		**Hodges-Lehmann**	
		**Median differences**	**95%CI**
Treatment	Placebo		
	Belimumab		
**Change from baseline in activated plasmablasts CD69+ at 12 months**		**Wilcoxon Rank Sum test**	
		**Mean Scores**	**p-value**
Treatment	Placebo		-
	Belimumab		
		**Hodges-Lehmann**	
		**Median differences**	**95%CI**
Treatment	Placebo		
	Belimumab		
**Change from baseline in activated plasmablasts CD69+ at 18 months**		**Wilcoxon Rank Sum test**	
		**Mean Scores**	**p-value**
Treatment	Placebo		-
	Belimumab		
		**Hodges-Lehmann**	
		**Median differences**	**95%CI**
Treatment	Placebo		

	Belimumab		
**Change from baseline in activated plasmablasts CD69+ at 24 months**		**Wilcoxon Rank Sum test**	
		**Mean Scores**	**p-value**
Treatment	Placebo		-
	Belimumab		
		**Hodges-Lehmann**	
		**Median differences**	**95%CI**
Treatment	Placebo		
	Belimumab		

Table 5.2.29: Change from baseline in activated plasmablasts CD95+ (see Table 2.4.1 for definition)

**Change from baseline in activated plasmablasts CD95+ at 3 months**		**Wilcoxon Rank Sum test**	
		**Mean Scores**	**p-value**
Treatment	Placebo		-
	Belimumab		
		**Hodges-Lehmann**	
		**Median differences**	**95%CI**
Treatment	Placebo		
	Belimumab		
**Change from baseline in activated plasmablasts CD95+ at 12 months**		**Wilcoxon Rank Sum test**	
		**Mean Scores**	**p-value**
Treatment	Placebo		-
	Belimumab		
		**Hodges-Lehmann**	
		**Median differences**	**95%CI**
Treatment	Placebo		
	Belimumab		
**Change from baseline in activated plasmablasts CD95+ at 18 months**		**Wilcoxon Rank Sum test**	
		**Mean Scores**	**p-value**
Treatment	Placebo		-
	Belimumab		
		**Hodges-Lehmann**	
		**Median differences**	**95%CI**
Treatment	Placebo		
	Belimumab		
**Change from baseline in activated plasmablasts CD95+ at 24 months**		**Wilcoxon Rank Sum test**	
		**Mean Scores**	**p-value**

Treatment	Placebo		-
	Belimumab		
		**Hodges-Lehmann**	
		**Median differences**	**95%CI**
Treatment	Placebo		
	Belimumab		

Clinical Efficacy

Definitions of events are given in Table 2.4.2.

Table 5.2.30: Time to clinical remission modelled using a Cox proportional hazards model adjusted for screening PR3 ANCA (<=24 or >24)

		**Median time to PR3 ANCA negativity**	**Number of events n/patients; (%)**	**HR (95% CI)**	**p-value**
Treatment	Placebo			1.00 (-)	-
	Belimumab				
Screening PR3 ANCA	Low (PR3 ANCA<=24)			1.00 (-)	-
	High (PR3 ANCA>24)				

Table 5.2.31: Proportion of participants in complete remission using mixed-effects logistic regression adjusted for screening PR3 ANCA (<=24 or >24)

**Outcome: Proportion of participants with PR3 negativity(ELISA) at 6 months**		**Comparison to Placebo**	
		**OR (95% CI)**	**p-value**
**Treatment**	Placebo	1.00 (-)	-
	Belimumab		
**Screening PR3 ANCA**	Low (PR3 ANCA<=24)	1.00 (-)	-
	High (PR3 ANCA>24)		
**12 months**			
		**OR (95% CI)**	**p-value**
**Treatment**	Placebo	1.00 (-)	-
	Belimumab		
**Screening PR3 ANCA**	Low (PR3 ANCA<=24)	1.00 (-)	-
	High (PR3 ANCA>24)		
**24 months**			
		**OR (95% CI)**	**p-value**
**Treatment**	Placebo	1.00 (-)	-
	Belimumab		
**Screening PR3 ANCA**	Low (PR3 ANCA<=24)	1.00 (-)	-
	High (PR3 ANCA>24)		

Table 5.2.32: Time to first relapse modelled using a Cox proportional hazards model adjusted for screening PR3 ANCA (<=24 or >24)

		Median time to first relapse	Number of events n/patients; (%)	HR (95% CI)	p-value
Treatment	Placebo			1.00 (-)	-
	Belimumab				
Screening PR3 ANCA	Low (PR3 ANCA<=24)			1.00 (-)	-
	High (PR3 ANCA>24)				

Table 5.2.33: Time to first major relapse modelled using a Cox proportional hazards model adjusted for screening PR3 ANCA (<=24 or >24)

		Median time to first major relapse	Number of events n/patients; (%)	HR (95% CI)	p-value
Treatment	Placebo			1.00 (-)	-
	Belimumab				
Screening PR3 ANCA	Low (PR3 ANCA<=24)			1.00 (-)	-
	High (PR3 ANCA>24)				

5.3 Additional / Exploratory Analyses

Table 5.3.1: Change from baseline in AAV-PRO

**Change from baseline in AAV-PRO at 3 months**		**Wilcoxon Rank Sum test**	
		**Mean Scores**	**p-value**
Treatment	Placebo		-
	Belimumab		
		**Hodges-Lehmann**	
		**Median differences**	**95%CI**
Treatment	Placebo		
	Belimumab		
**Change from baseline in AAV-PRO at 6 months**		**Wilcoxon Rank Sum test**	
		**Mean Scores**	**p-value**
Treatment	Placebo		-
	Belimumab		
		**Hodges-Lehmann**	

		**Median differences**	**95%CI**
Treatment	Placebo		
	Belimumab		
**Change from baseline in AAV-PRO at 12 months**		**Wilcoxon Rank Sum test**	
		**Mean Scores**	**p-value**
Treatment	Placebo		-
	Belimumab		
		**Hodges-Lehmann**	
		**Median differences**	**95%CI**
Treatment	Placebo		
	Belimumab		
**Change from baseline in AAV-PRO at 18 months**		**Wilcoxon Rank Sum test**	
		**Mean Scores**	**p-value**
Treatment	Placebo		-
	Belimumab		
		**Hodges-Lehmann**	
		**Median differences**	**95%CI**
Treatment	Placebo		
	Belimumab		
**Change from baseline in AAV-PRO at 24 months**		**Wilcoxon Rank Sum test**	
		**Mean Scores**	**p-value**
Treatment	Placebo		-
	Belimumab		
		**Hodges-Lehmann**	
		**Median differences**	**95%CI**
Treatment	Placebo		
	Belimumab		

Table 5.3.2: Change from baseline in Vasculitis Damage Index (VDI)

**Change from baseline in VDI at 6 months**		**Wilcoxon Rank Sum test**	
		**Mean Scores**	**p-value**
Treatment	Placebo		-
	Belimumab		
		**Hodges-Lehmann**	
		**Median differences**	**95%CI**

Treatment	Placebo		
	Belimumab		
**Change from baseline in AAV-PRO at 12 months**		**Wilcoxon Rank Sum test**	
		**Mean Scores**	**p-value**
Treatment	Placebo		-
	Belimumab		
		**Hodges-Lehmann**	
		**Median differences**	**95%CI**
Treatment	Placebo		
	Belimumab		
**Change from baseline in AAV-PRO at 18 months**		**Wilcoxon Rank Sum test**	
		**Mean Scores**	**p-value**
Treatment	Placebo		-
	Belimumab		
		**Hodges-Lehmann**	
		**Median differences**	**95%CI**
Treatment	Placebo		
	Belimumab		
**Change from baseline in AAV-PRO at 24 months**		**Wilcoxon Rank Sum test**	
		**Mean Scores**	**p-value**
Treatment	Placebo		-
	Belimumab		
		**Hodges-Lehmann**	
		**Median differences**	**95%CI**
Treatment	Placebo		
	Belimumab		

Missing data: for all secondary analyses, missing outcome data will be excluded from the analysis. For endpoints where a mixed-effects model is used, participants with missing outcomes for one or more timepoints will still be included in the analysis. For other endpoints, a complete cases analysis will be conducted.

For endpoints representing a negative clinical event, if an outcome is missing due to death, we will do a secondary analysis that treats death as part of the negative clinical event. For example, when analysing the outcome 'major progressive disease prior to remission', if a participant died prior to this being determinable, results from excluding that participant and including that participant as major progressive disease would be presented separately.

6. SAFETY

Table 6.1: Line listing of laboratory abnormalities.

ID	Visit Date	Event Status	Lab Abnormality	Date of Test	Action taken	Outcome	Date of Resolution	Randomization Date

Table 6.2: Line listing of the AEs from the *AESI - Other form.*

ID	Visit Date	Event No	Event Status	Details of event	Date of Onset	Outcome	Date of Resolution	Treatment of event including duration and dose	Action taken with IMP	Relatedness to IMP	Causality	Maximum Itensity	Oral prednisolone dose at time of event	Did the patient withdraw from the study	Randomisation Date

Table 6.3: Infection form.

ID	Infection Number	Infection Status	Site of Infection	Organisms Name (or 'not known')	Cultured?	Date of Onset	Outcome	Date of Resolution	Treatment administered for infection	Action taken with IMPs	Maximum Itensity	Oral prednisolone dose at time of infection	Most recent IgG value	Was this infection serious?

6.1 Adverse events

These analyses will be performed in the safety population

Only AEs of special interest (AESI) should be recorded.

Adverse Events of Special Interest:

- Infections requiring antimicrobial, antiviral or antifungal treatment (including opportunistic, PML)
- Hypogammaglobinaemia
- Systemic infusion/injection reactions
- Hypersensitivity reactions
- Malignancy
- Psychiatric events (including suicidality)
- Severe skin reactions (including Toxic Epidermal Necrolysis and Stevens-Johnson syndrome)
- Cardiac disorders (including angina, myocardial infarction, arrhythmia, heart failure)
- Thromboembolic event
- PRES
- Pregnancy

Adverse events (AEs) will be provided coded according to the latest version of MedDRA available. All AEs will be tabulated by preferred term and (worst reported) severity, graded on a three-point scale (mild, moderate, severe), in each randomisation group, sorted by body system. This means that only one of each type of event (worst reported) is reported per participant. The number of subjects and number of occurrences of each event will also be tabulated as required by EudraCT.

Table 6.1.1: Adverse events (worst reported)

Adverse event	Grade	Belimumab (N=)		Placebo (N=)	
		N	%	N	%
	None<br>Mild<br>Moderate<br>Severe				
	None<br>Mild<br>Moderate<br>Severe				
	None<br>Mild<br>Moderate				

	Severe				
	None<br>Mild<br>Moderate<br>Severe				

Table 6.1.2 Number of subjects affected by non-serious adverse events – for EudraCT

	Belimumab (N=)		Placebo (N=)		Overall (N=)	
	N	%	N	%	N	%
Event 1						
Event 2						
Event 3						
**No. affected by non-serious AE**						

Table 6.1.3 Occurrence of non-serious adverse events – for EudraCT

	Belimumab (N=)		Placebo (N=)		Overall (N=)	
	N	%	N	%	N	%
Event 1						
Event 2						
**No. affected by non-serious AE**						

6.2 Serious adverse events

Table 6.2.1: Line listing of all SAEs

ID	SAE no.	Tx. group	Tx. Start	Tx. end	Onset date	Description	Severity	Causality	Outcome	Outcome date

7. Statistical software

Data will be output directly from MACRO into a STATA-readable format by the CTU. Statistical analyses will be carried out by the Trial Statistician at the PHSI using Stata version 15 or R version 4. All programs and output will be stored in the School Statistics folder on the PHSI server.

References

1. Nagai M, Hirayama K, Ebihara I, Shimohata H, Kobayashi M, Koyama A. Serum Levels of BAFF and APRIL in Myeloperoxidase Anti-Neutrophil Cytoplasmic Autoantibody-Associated Renal Vasculitis: Association with Disease Activity. Nephron Clinical Practice 2011; 118(4):c339–45.

2. Krumbholz M, Specks U, Wick M, Kalled SL, Jenne D, Meinl E. BAFF is elevated in serum of patients with Wegener's granulomatosis. Journal of Autoimmunity. 2005; 25(4):298–302.

3. Xin G, Su Y, Li K-S, Chen M, Zhao M-H, Xu L-X. Serum B-cell Activating Factor in Myecloperoxiase-antineutrophil Cytoplasmic Antibodies-associated Vasculitis. The American Journal of the Medical Sciences. 2014; 348(1):25–9.

4. Schneeweis C, Rafalowicz M, Feist E, Buttgereit F, Rudolph P-E, Burmester G-R, et al. Increased levels of BLyS and sVCAM-1 in anti-neutrophil cytoplasmatic antibody (ANCA)-associated vasculitides (AAV). Clinical and Experimental Rheumatology. 2010 28(1 Suppl 57):62–6.

5. Sanders J-SF, Huitma MG, Kallenberg CGM, Stegeman CA. Plasma levels of soluble interleukin 2 receptor, soluble CD30, interleukin 10 and B cell activator of the tumour necrosis factor family during follow-up in vasculitis associated with proteinase 3-antineutrophil cytoplasmic antibodies: associations with disease activity and relapse. Annals of the Rheumatic Diseases. 2006 Nov; 65(11):1484–9.

6. Bader L, Koldingsnes W, Nossent J. B-lymphocyte activating factor levels are increased in patients with Wegener's granulomatosis and inversely correlated with ANCA titer. Clinical Rheumatology 2010 Sep; 29(9):1031-5.

7. Holden NJ, Williams JM, Morgan MD, Challa A, Gordon J, Pepper RJ, et al. ANCA-stimulated neutrophils release BLyS and promote B cell survival: a clinically relevant cellular process. Annals of the Rheumatic Diseases. 2011; 70(12):2229–33.

8. Jennette JC, Falk RJ, Bacon PA, Basu N, Cid MC, Ferrario F, et al. International Chapel Hill Consensus Conference Nomenclature of Vasculitides. Arthritis & Rheumatology. 2013 Jan;65(1):1–11.

9. Stone JH, Hoffman GS, Merkel PA, Min YI, Uhlfelder ML, Hellmann DB, et al. A disease-specific activity index for Wegener's granulomatosis: modification of the Birmingham Vasculitis Activity Score. International Network for the Study of the Systemic Vasculitides (INSSYS). Arthritis & Rheumatology. 2001 Apr; 44(4):912–20.

10. Sanders J-SF, Huitma MG, Kallenberg CGM, Stegeman CA. Prediction of relapses in PR3-ANCA-associated vasculitis by assessing responses of ANCA titres to treatment. Rheumatology. 2006; 45(6):724–9.

APPENDIX

Appendix 1: COMBIVAS 3-month exploratory biomarker analysis plan

3-month exploratory endpoint analysis overview

Whilst the final data analysis (including all primary and secondary endpoints) will occur following trial completion (LSLV March 2023), an unblinded analysis of early mechanistic biomarker endpoints is planned prior to trial completion - report due in Oct 2022. Non-patient-facing members of the COMBIVAS trial team will be unblinded to allow the analysis of baseline demographic data and mechanistic exploratory endpoints up to 3 months.

The four planned analyses include:

1) Assessment of belimumab-induced early B cell mobilisation from the tissue (Day 1- pre first belimumab/placebo SC dose) to the circulation (Day 8- pre first rituximab dose)
2) Assessment of the effect of rituximab + belimumab/placebo on blood and tissue at 3 months.
3) Quantification of urinary lymphocytes at baseline and Month 3
4) Assessment of serum BLyS concentrations at baseline and Month 3

Flow cytometric and single cell RNA-sequencing (scRNA-seq) data is available for samples from Day 1 (blood, lymph node, nasal tissue), Day 8 (blood only) and Month 3 (blood, lymph node, nasal tissue). Flow cytometry was performed (B lymphocytes) on blood using standardized GSK panels by the GLP approved CUC lab on Day 1 and Day 8 (appendix 1.1), and also by the University of Cambridge using extended panels (B and T lymphocytes) on blood, nasal and lymph node samples taken simultaneously (at baseline and month 3)- to allow detailed cross compartment comparisons (appendix 1.2). Flow cytometric data is available for samples from Day 1 and Month 3 on a subset of patients. Measurements of serum BLyS concentration is available from Day 1 and Month 3 samples for all patients.

Analysis 1: Assessment of belimumab-induced B cell mobilisation from the tissue (Day 1) to the circulation (Day 8)

Flow cytometric analysis of circulating B cells and proliferation markers on Day 1 and Day 8 samples is available for a subset of the cohort (paired samples for n=19). This data has been analysed in the GSK CUC, GLP accredited laboratory (see appendix 1.1 for flow cytometry

panel and reportables). Baseline demographics will be available; key variables include steroid, cyclophosphamide and previous rituximab exposure prior to trial entry.

scRNA-seq data (University of Cambridge) is available for a subset of patients for Day 1 (blood, lymph node, nasal tissue) and Day 8 (blood only) samples. Most (but not all) patients with scRNA-seq data also have complementary flow cytometric data.

Table 1 scRNA-seq data available for analysis 1.

Timepoint	Compartment	Number of samples
Baseline (on or before Day 1)	Blood	16
	Nasal	14
	Lymph node	11
Day 8	Blood	15
	Total	56

Suggested approaches to evaluate belimumab-induced B cell mobilisation from the tissue to the circulation by scRNA-seq:

1. Accurate identification and annotation of B cells and B cell subsets in blood.
2. Attempt to identify blood B cells from Day 8 post-belimumab samples that express tissue-specific molecular signatures defined from Day 1 tissue samples (nasal and lymph node).
3. Attempt to identify blood B cell clusters that are predominantly made up of cells from Day 8 belimumab samples to permit differential gene expression analysis and identification of biological processes or pathways specific to belimumab treatment.
4. Directly compare the expression profiles of lymphocyte tissue residency markers and lymphocyte trafficking markers expressed in belimumab versus placebo treated B cells

Analysis 2: Assessment of the effect of rituximab +/- belimumab on blood and tissue at 3 months compared to baseline.

Flow cytometric data on B cells and T cells (see appendix 1.2 for full panels; data collected in University of Cambridge) from baseline and Month 3, with samples taken simultaneously from blood, nasal and lymph node biopsies is available for a subset of patients. Baseline demographics also available.

Table 2. Flow cytometric data available for analysis 2.

Timepoint	Compartment	Number of samples
Baseline (on or before Day 1)	Blood	36
	Nasal	28
	Lymph node	18
Month 3	Blood	31
	Nasal	26
	Lymph node	21

Complementary scRNA-seq data is available for a subset of patients for Day 1 and Month 12 (blood only) samples. See Table 3 for available data.

Table 3 scRNA-seq data available for analysis 2.

Timepoint	Compartment	Number of samples
Baseline (on or before Day 1)	Blood	16
	Nasal	14
	Lymph node	11
Month 3	Blood	12
	Nasal	12
	Lymph node	8
	Total	73

Suggested approaches to evaluate the effects of rituximab +/- belimumab on immune and non-cells in the tissue by scRNA-seq:

1. Accurate identification and annotation of immune and non-immune cells
2. Quantification of B cell and T cell subsets across compartments/timepoints and comparison between belimumab and placebo
3. Differential gene expression of tissue B cells between belimumab and placebo
4. Pathway enrichment analysis / GSEA

Type of data

The scRNA-seq libraries were generated using 10x Genomics Chromium Single Cell V(D)J Reagents Kits (v1 Chemistry, https://support.10xgenomics.com/single-cell-vdj/library-prep/doc/user-guide-chromium-single-cell-vdj-reagent-kits-v1-chemistry). Cells were suspended in PBS at ~2000 cells/μL

and loaded onto a single lane of the Chromium Controller. Captured cell number was 5000-10000 cells/channel (1 sample = 1 channel). Libraries were sequenced by Genewiz (Leipzig, Germany) on an Illumina Novaseq S4 flowcell gaining reads per cell of >20000. Mapping and quantification was performed using the 10X Genomics Cell Ranger software package.

Appendix 1.1

Data from the abbreviated B cell panel and MTBNK panel will be analysed (in GSK CUC laboratory) to provide the following outputs for each subject on blood:

Cell type	Analyte	Reportable	Day 1 (pre-first belimumab/placebo dose)	Day 8 (pre first rituximab dose)
B cells	CD45+CD19+	Absolute cell count<br>%CD45		
Memory B cells	CD45+CD19+CD27+	Absolute cell count<br>%CD19<br>%CD45		
Memory B cells Ki67+	CD45+CD19+CD27+Ki67+	Absolute cell count<br>%CD45+CD19+CD27+		
Non-switched memory B cells	CD45+CD19+CD27+IgD+	Absolute cell count<br>%CD19<br>%CD45		
Non-switched memory B cells Ki67+	CD45+CD19+CD27+IgD+Ki67+	Absolute cell count<br>%CD45+CD19+CD27+IgD+		
Switched memory B cells	CD45+CD19+CD27+IgD-	Absolute cell count<br>%CD19<br>%CD45		
Switched memory B cells Ki67+	CD45+CD19+CD27+IgD-Ki67+	Absolute cell count<br>%CD45+CD19+CD27+IgD-		

Appendix 1.2

Data from the exploratory B and T cell panel will be analysed (in University of Cambridge) to provide the following outputs for each subject on simultaneous blood, nasal and lymph node samples:

Cell type	Analyte	Reportable	Day 1	Week 12
B cells	CD45+CD19+	%CD45		
B cells (CD20)	CD19+CD20+	%CD19		
Memory B cells	CD45+CD19+CD27+	%CD19		
Non-switched memory B cells	CD45+CD19+CD27+IgD+	%CD19		
Switched memory B cells	CD45+CD19+CD27+IgD-	%CD19		
Double negative B cells	CD19+IgD-CD27-	%CD19		
Transitional B cells	CD19+CD24+CD38+	%CD19		
Plasmablasts	CD19+IgD-CD27+CD20-CD38+	%CD19		
T cells	CD3+	%CD45		
CD4+ T cells	CD4+	%CD3		
Naïve T cells	CD4+CD45RA+CCR7+	%CD4		
Effector memory T cells	CD4+CD45RA-CCR7-	%CD4		
Central memory T cells	CD4+CD45RA-CCR7+	%CD4		
TEMRA cells	CD4+CD45RA+CCR7-	%CD4		
T regulatory cells	CD4+CD127loCD25hi	%CD4		

TfH cells	CD4+CXCR5+PD1hi	%CD4		

Data exported from FACS Diva software will be loaded into two separate FlowJoTM worksheet templates designed for B cell and T cell immunophenotyping. Compensation was calculated at acquisition for T cell panel, and so no further compensation alterations are required. A compensation matrix shall be made for each time compensation samples were analysed for the B cell panel and will be applied to the samples analysed after the compensation matrix was made, until a new compensation matrix is made. To maintain consistency with gating, a standardised gating strategy with a clear set of rules will be used for each panel will be followed.

Figure 1. B cell gating strategy

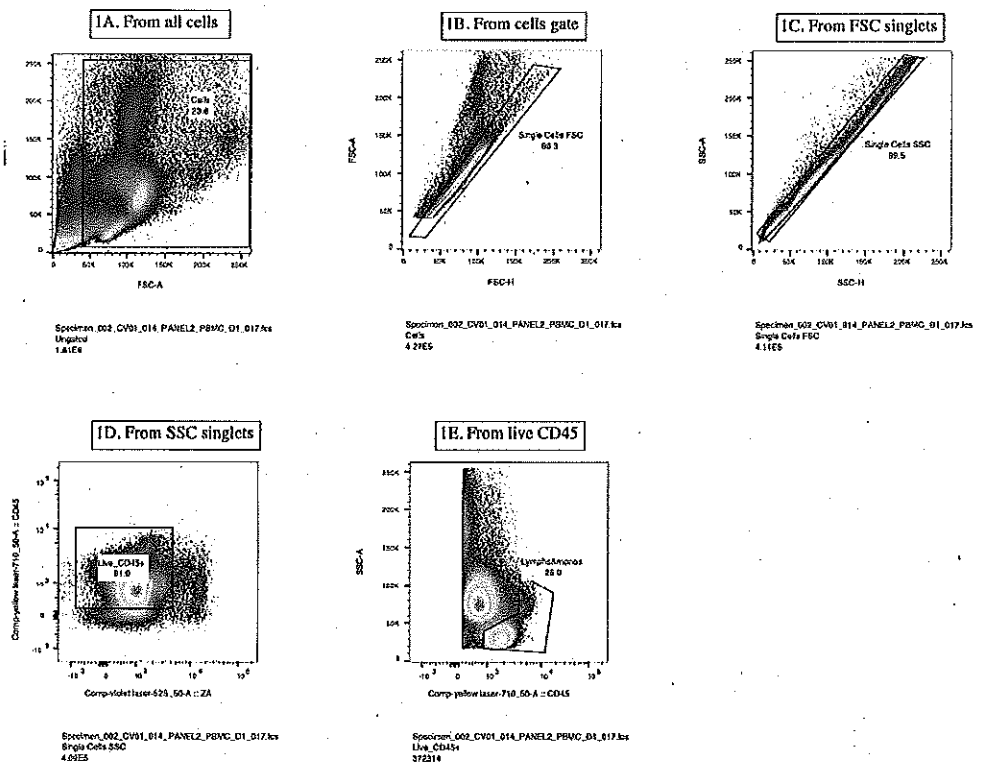

1F. From lymphs&monos

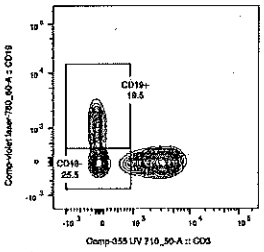

Specimen_002_CV01_014_PANEL2_PBMC_D1_017.fcs
Lymphs&monos
93165

1G. From lymphs&monos

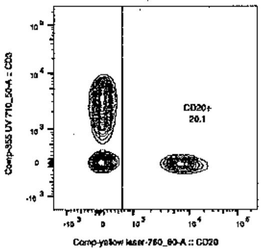

Specimen_002_CV01_014_PANEL2_PBMC_D1_017.fcs
Lymphs&monos
93165

1H. From CD19+

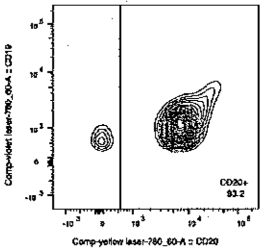

Specimen_002_CV01_014_PANEL2_PBMC_D1_017.fcs
CD19+
18107

1I. From lymphs&monos

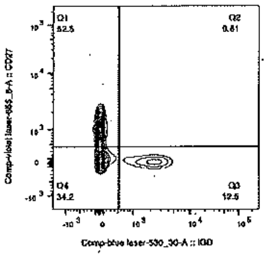

Specimen 002 CV01 014 PANEL2 PBMC D1 017.fcs

1J. From CD19+

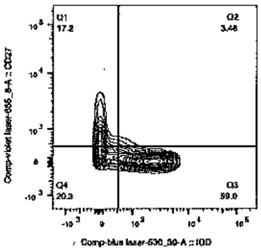

Specimen 002 CV01 014 PANEL2 PBMC D1 017.fcs

1K. From CD19-

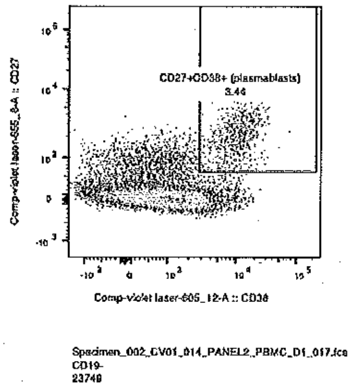

1L. From CD19+

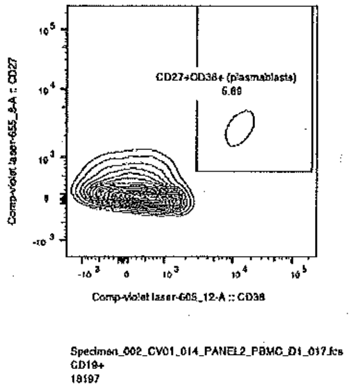

1M. From CD19+

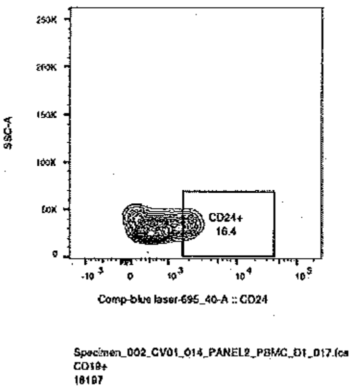

1N. From CD19+

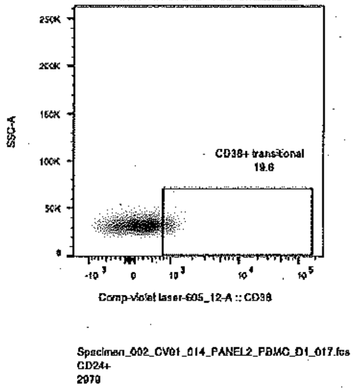

1O. From CD19+CD27-IgD+

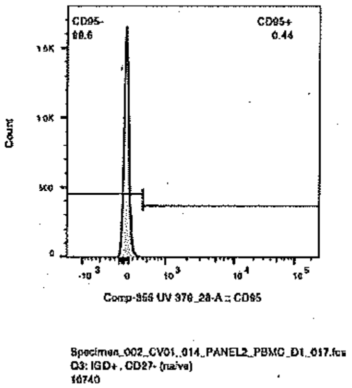

1. A gate called “Cells” will be applied to the root gate. This wide gate will exclude debris and granulocytes with either a low or a high forward scatter (FSC-A) value and a very high side scatter (SSC-A) value. This gate will ensure only cell population of interest will be retained (Figure 1A).
2. Doublets will be excluded by gating on FSC-A and FSC-H (Figure 1B) followed by SSC-A and SSC-H dimensions (Figure 1C).
3. Live CD45+ cells will be identified by gating CD45+ cells and excluding cells that stain for Zombie aqua (Figure 1D).

4. Lymphocytes and monocytes will be identified by CD45 expression and SSC-A characteristics (Figure 1E). The position will be confirmed by back-gating on CD19 B cells.
5. From the Lymphocytes and monocytes gate, CD19+ B cells will be identified by plotting CD19 (y-axis) vs CD3 (x-axis). Using contour plots, the top edge of the CD19- population will be used as the bottom edge of the CD19+ population (Figure 1F).
6. The CD20+ gate will be identified by plotting CD3 (y-axis) vs CD20 (x-axis) on the lymphocyte and monocyte gate. Using contour plots, the right-hand edge of the CD3+CD20- population will be the reference for CD20 positivity (Figure 1G) and applied to the CD19+ population (Figure 1H).
7. The distinction between memory B cell subsets and naïve B cells will be standardised by placing a quadrant gate firstly on the lymphocyte and monocyte population whilst plotting CD27 (y-axis) vs IgD (x-axis) (using contour plots, Figure 1I), and then applying this gate to the CD19+ population (Figure 1J).
8. Plasmablasts will be identified by first setting a CD27+CD38+ gate on the CD19- population when visualizing CD27 (y-axis) vs CD38 (x-axis) with contour plots. The bottom edge of the CD27+ population and the left-hand edge of the CD38Br population will be used to standardise the gate position (Figure 1K) which will be then transferred onto the CD19+ population (Figure 1L).
9. Transitional B cells will be identified in two stages using contour plots: firstly, CD24+ cells will be identified from the CD19+ population (Figure 1M); then CD38+ gate will be applied to the CD19+CD24+ population (Figure 1M).
10. To find activated cells, CD69 and CD95 positivity will be defined using histograms for each marker on naïve B cells (Figure 10). The positive gate can then be applied to switched memory B cells (CD19+IgD-CD27+), non-switched memory B cells (CD19+IgD+CD27+), double negative B cells (CD19+IgD-CD27-), plasmablasts (CD19+ CD27+CD38+) and all B cells (CD19+).
11. Mean fluorescence intensity (MFI) of CD69 and CD95 will also be calculated for switched memory B cells (CD19+IgD-CD27+), non-switched memory B cells (CD19+IgD+CD27+), double negative B cells (CD19+IgD-CD27-), plasmablasts (CD19+ CD27+CD38+) and all B cells (CD19+).

<u>Figure 2: T Cell Panel</u>

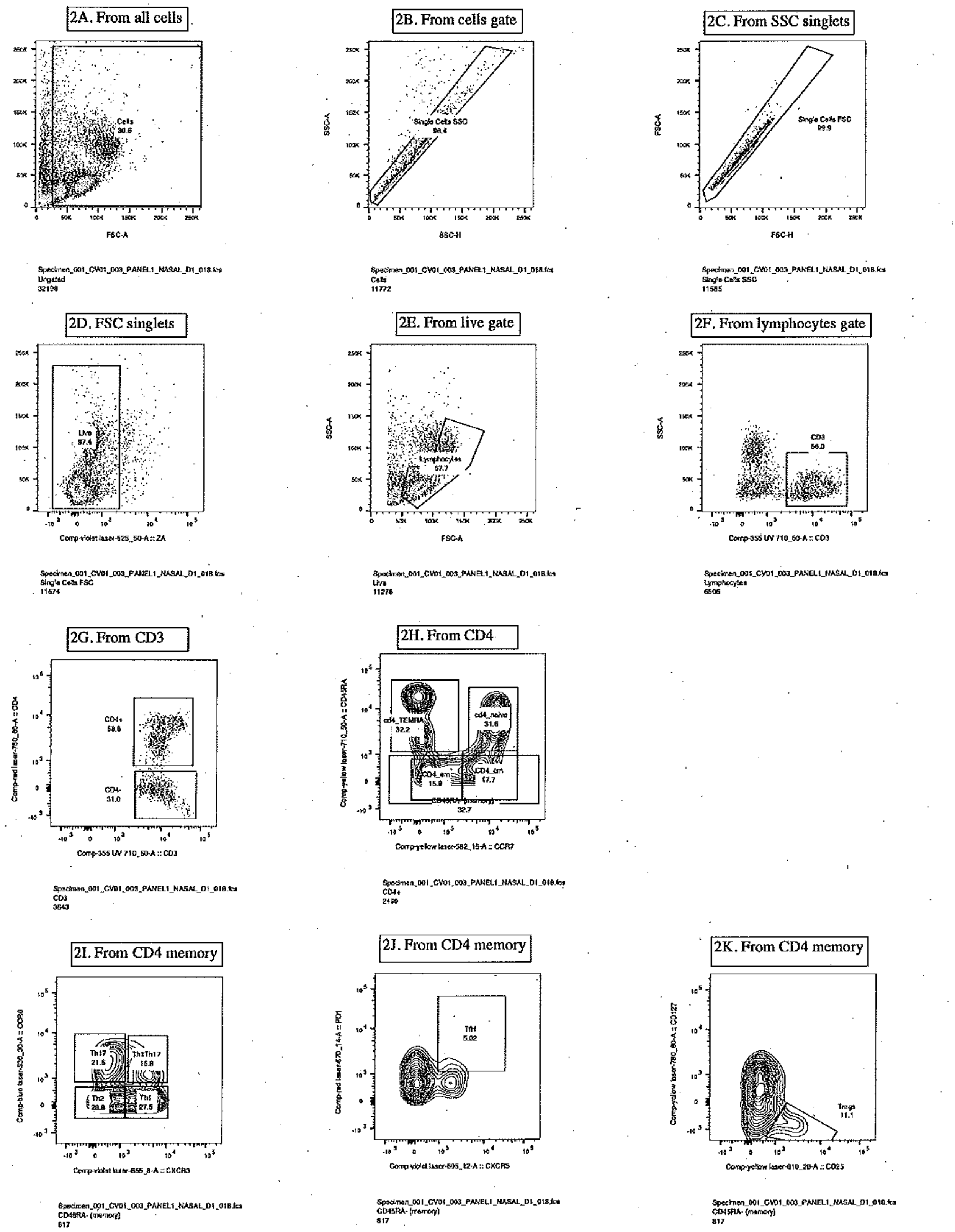
2A. From all cells
Cells
FSC-A
2B. From cells gate
Single Cells SSC
SSC-A
SSC-H
2C. From SSC singlets
Single Cells FSC
FSC-A
FSC-H
2D. FSC singlets
Live
97.4
2E. From live gate
Lymphocytes
57.7
SSC-A
FSC-A
2F. From lymphocytes gate
CD3
SSC-A
2G. From CD3
CD4+
CD4-
31.0
2H. From CD4
cd4_TEMRA
32.2
cd4_naive
31.6
CD4_em
15.9
CD4_cm
17.7
32.7
2I. From CD4 memory
Th17
21.5
Th1Th17
Th2
Th1
27.5
2J. From CD4 memory
Tfh
5.02
2K. From CD4 memory
Tregs
11.1

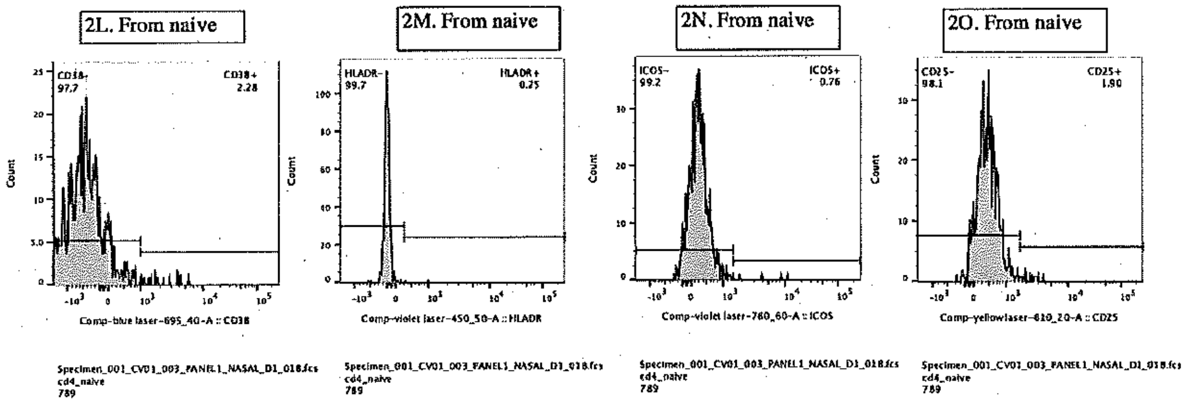

1. A gate called "Cells" will be applied to the root gate. This wide gate will exclude debris and granulocytes with a low forward scatter (FSC-A) value and a high side scatter (SSC-A) value (Figure 2A).
2. Doublets will be excluded by gating on SSC-A and SSC-H (Figure 2B) followed by FSC-A and FSC-H dimensions (Figure 2C).
3. Live cells will be identified by gating SSC-A cells (y-axis) versus Zombie aqua (x-axis) and excluding cells that stained for Zombie aqua (Figure 2D).
4. Lymphocytes will be identified by FSC-A and SSC-A characteristics (Figure 2E).
5. CD3 T cells will be identified as those expressing CD3 marker within the lymphocyte population (Figure 2F).
6. CD4 T cells will be gated on cells expressing CD4 marker within the CD3 population (Figure 2G). CD8 T cells will be identified as the CD3+CD4- population.
7. From the CD4 population, naïve cells will be identified as CCR7+ and CD45RA+. TEMRA cells are identified as CCR7- and CD45RA+. The boundary between CD45RA- CCR7+ and CCR7- populations can be sometimes difficult to determine so the whole population would be defined as memory cells for further analysis. If two clear populations can be visualized by contour plot and using the TEMRA and Naïve populations as gating location, T effector memory CCR7- and T central memory CCR7- can then be gated (Figure 2H). If no distinct populations can be identified, only naïve and memory populations will be recorded.
8. For CD4 T helper subsets, a quadrant gate will be placed whilst plotting CCR6 (y-axis) vs CXCR3 (x-axis) on the CD4 memory population. Only gate around clearly defined populations (Figure 2I). If distinct subsets cannot be visualized, then 'NA' will be recorded.
9. For CD4 T follicular helper cells, a gate will be placed whilst plotting PD1 (y-axis) vs CXCR5 (x-axis). Only gate around clearly defined populations. If distinct subsets cannot be visualized, then 'NA' will be recorded (Figure 2J)

10. T regulatory cells (Tregs) will be defined by CD127loCD25hi markers. The border between non-Tregs and Tregs is defined by the line of lowest density between these populations in a biplot of CD127 versus CD25 (Figure 2K).
11. To find activated cells, use the naïve population on a histogram of CD38, CD25, HLA-DR and ICOS and set the negative gate at the naïve population (Figure 2L-O). Then use the same gate for all other cells.
12. Mean fluorescence intensity (MFI) of CD38, CD25, HLA-DR and ICOS will also be calculated for all subsets.
