# Supplementary material for: A randomised study of rituximab and belimumab sequential therapy in PR3 ANCA-associated vasculitis (COMBIVAS): design of the study protocol
Source: Trials. 2023 Mar 11;24:180. doi: 10.1186/s13063-023-07218-y (PMC10007661; doi:10.1186/s13063-023-07218-y)
Supplement: Supplementary file 6 — Additional file 6. Participant information sheet summary. [file 13063_2023_7218_MOESM6_ESM.pdf]

PARTICIPANT INFORMATION SHEET SUMMARY

COMBIVAS: A randomised, double blind, controlled mechanistic trial of rituximab and belimumab combination therapy in PR3 ANCA-associated vasculitis

In this trial, we want to test whether combining two medicines (rituximab and belimumab) that target specific immune cells called B cells is better than just giving rituximab alone for treating ANCA associated vasculitis.

30 patients will be randomly assigned to receive either rituximab plus belimumab or rituximab plus placebo (dummy drug).

Rituximab is given on Day 8 and Day 22. It will be administered by a trial nurse through a needle placed in a vein (IV infusion).

Belimumab or placebo is given on Day 1 and then once a week for 52 weeks (52 doses in total). It is given by injection delivered just under the skin (subcutaneous). We will teach you how to do the injection so you can do it at home.

Both groups will also receive low dose steroid, aiming to stop completely by 3 months.

There are 3 phases to the trial:

1. Screening period (lasts up to 14 days): when we will do some tests (e.g. blood tests) to check you are suitable for the trial.

2. Treatment period (lasts for 12 months): 11 visits to the clinic are required during this period (every 4 weeks for the first 6 months, then every 8 weeks for 6 months. 2 extra visits during the first month are needed for the rituximab infusions (Day 8 and Day 22).

3. Follow-up period (lasts for 12 months): 4 visits to the clinic are required during this period (every 3 months).

Therefore, you will need to come to hospital 18 times over a period of 2 years and 2 weeks.

At each visit, information about how the trial drug that you receive affects your body and your health will be collected through a number of tests, procedures and questions.

At the beginning of the trial and at Week 12, we will ask if you are willing to undergo a lymph node biopsy which is a minor surgical procedure to sample minor lymph glands from your groin, and a nasal tissue biopsy, which is commonly done to investigate the severity of vasculitis in the nose. These are generally well tolerated procedures that are done under local anaesthetic.

If you would like to know more about this trial, then please read the full participant information sheet and consent form attached.

Many thanks for your time and interest.

PARTICIPANT INFORMATION SHEET & INFORMED CONSENT FORM

COMBIVAS: A randomised, double blind, controlled mechanistic trial of rituximab and belimumab combination therapy in PR3 ANCA-associated vasculitis

You are being invited to take part in a research trial. Before deciding whether to take part, you need to understand why this research is being done and what it involves. Please take time to read the following information carefully and talk to others about the trial if you wish. Please ask us if anything is not clear or if you would like more information. Please take time to decide whether or not you wish to take part.

Section 1 tells you the purpose of this trial and what will happen to you if you take part.
Section 2 gives you more detailed information about the conduct of the trial.

Section 1: Purpose of the trial and what will happen

1. What is the purpose of the trial?

The purpose of this trial is to test the safety and effectiveness of belimumab in combination with rituximab in vasculitis. We want to find out how the 2 trial drugs work together.

The trial will also form part of the PhD (Doctor of Philosophy) higher education degrees for the trial physicians Dr Mark McClure and Seerapani Gopaluni.

2. What is the drug being tested?

The drugs being tested are belimumab (also known as Benlysta™) and rituximab (also known as Truxima™, MabThera™ or Rixathon™).

ANCA vasculitis is a disease in which the immune system (the system that fights infection) attacks your own cells and tissues, causing inflammation of the blood vessels. It can affect almost any organ in the body, and is thought to involve ANCA antibodies (proteins made in the body in response to a foreign substance) produced by a type of white blood cell called B-cells. The drugs being tested, belimumab and rituximab, both work in different ways to inhibit B-cells. Therefore, it is hoped that belimumab given in combination with rituximab, may produce a beneficial effect on the symptoms and progression of the disease.

In many countries, belimumab and rituximab are approved medicines when given as individual therapies (not combined) for the treatment of diseases which involve excess activity of the immune system. However, only rituximab is currently approved for doctors to use to treat patients with ANCA vasculitis. Belimumab is not currently approved for doctors to treat patients with vasculitis.

There is a good scientific rationale for combining these two medications for the treatment of vasculitis and the combination is currently being tested in similar inflammatory conditions including systemic lupus erythematosus (also called “lupus” or “SLE”). It is not yet known if the combination is effective and safe in people with ANCA vasculitis.

3. Why have I been invited?

You have been invited to participate in this trial because you have a particular type of vasculitis (ANCA associated vasculitis) with active disease, and we believe that the addition of

belimumab or placebo to the standard of care treatment with rituximab may be a suitable treatment for you.

We plan to include at least 30 participants with ANCA vasculitis from 5 hospitals across the UK in this trial.

4. Do I have to take part?

Participating in this trial is completely voluntary. If you decide to participate you will be asked to sign an Informed Consent Form, however you are still free to change your mind and leave the trial at any time without giving a reason. If you choose not to participate or to leave the trial, your future medical treatment and normal standard of care will not be affected in any way.

5. What will happen to me if I take part?

If you agree to take part in the trial, you will sign the Informed Consent Form at the end of this document and will be given a copy of this to take away and refer to later.

Before you enter into the trial, you will undergo some 'screening assessments' to check if you are eligible to take part in the trial. During the 'screening visit', your trial doctor will ask you about your medical history and any medications you are taking. You will have a physical examination including an ECG (tracing of the heart), and a blood sample (approximately 40 mL, equivalent to 2 tablespoons) and a urine sample will be taken for laboratory tests.

If you qualify for the trial and decide to participate, a computer will put each participant into treatment groups by chance. Neither you nor the trial doctor can choose a group. This is called 'randomisation'. You have a 50% chance of being placed in either group.

The two trial arms (treatment groups) are as follows:

1. Belimumab treatment arm:

People in this arm will receive belimumab subcutaneously once a week for the first 52 weeks and will then be followed up for an additional 52 weeks. In addition, rituximab infusion will be given on days 8 and 22.

2. Placebo treatment arm:

People in this arm will receive belimumab placebo (a medication that looks the same but does not have any drug in it) once a week for the first the 52 weeks and will then be followed up for an additional 52 weeks. In addition rituximab will be given on days 8 and 22.

This is a 'double-blind' trial - neither you nor the trial doctor will know which group you are in. However, your trial doctor can find out if necessary. This way, the findings from the two groups will be looked at in the same way.

Information about how the trial drug that you receive affects your body and your health will be collected through a number of tests, procedures and questions.

Additionally, at the beginning of the trial and at Week 12, we will ask you if you are willing to undergo a lymph node biopsy which is a minor surgical procedure to sample minor lymph

glands from your groin. At the beginning of the trial and at week 12, we will also ask you if you are willing to undergo a nasal tissue biopsy, which is commonly done to investigate the severity of vasculitis in the nose.
These procedures are optional, if you are not willing to undergo these procedures you can still take part in this trial.

6. What will I have to do?

Your expected participation in this trial will last about 2 years. During this time, you will have some tests, visit the clinic on schedule, and tell the trial staff about any changes to your health.

You will receive belimumab (200mg per dose) or matching placebo every week for a maximum of 52 weeks as an injection delivered just under the skin (subcutaneous). You/your carer will inject the first dose of belimumab/placebo in the trial clinic under the supervision of your doctor to ensure you understand what to do and that your doctor is satisfied that you can do it properly. You will need to stay in the clinic for 3 hours for observation after the first dose. You/your carer will then inject the remaining doses of belimumab or placebo at home.

At the time of the first belimumab or matching Placebo injection, you will also be given a course of steroids (prednisolone) to help get your vasculitis symptoms under control. The dose will start at 20 mg/day and will gradually be reduced with a plan to stop by Week 12. You will be given written instructions about what dose to take and when to reduce the dose. If you are on immunosuppressants (e.g., methotrexate, azathioprine, or mycophenolate), these drugs will be stopped at Day 1 of the trial before the trial drugs are administered.

You will receive a supply of belimumab or matching placebo (referred to as the trial drug kit) at each trial visit which will be required to be kept in the refrigerator. You will also be given detailed instructions on how to self-inject, a sharps container to throw away your used syringes, a cool bag for transportation, and instructions on how to store your trial drug at home. You will need to bring your sharps container with all used syringes back to the clinic at each visit, as well as any unused syringes. You will also receive a self-injection diary which you will take home and complete after each dose and which you will bring back to the clinic with you at each scheduled visit.

The injections are to be given subcutaneously (under the skin in the abdomen or thigh) once every week, on the same day (or 1 day either side) each week. If you miss your dose by more than one day, you should wait until the next week to take the next dose on your usual day. If you miss 4 or more injections in a row, you will need to have your next injection at the clinic and stay for 3 hours for observation.

When injecting in the same region, use a different injection site each week. Do not inject into areas where the skin is tender, bruised, red, or hard.

You will receive rituximab (1000mg per dose) on days 8 and 22 at your local hospital, administered by a trial nurse or trial doctor through a needle placed in a vein (IV infusion). Prior to receiving rituximab, you will receive medicines to reduce the risk of an allergic reaction. These will be an antihistamine, a steroid and paracetamol (to prevent pain and fever). It takes at least 3-4 hours to give you the full dose followed by an observation period of 1 hour.

The trial is split into a number of periods as shown in the table below. The table describes the tests you will receive. During the screening period you will have examinations, tests and procedures to find out if you can take part in the trial.

The treatment period is when you receive the trial medicines and lasts for 1 year. After the treatment period, you will enter a follow up period of 1 year with visits every 3 months for a year during which you will continue to be monitored and assessed by your doctor (but will not receive any more trial treatments).

**Procedure** (D=Day, W=Week)	**Year 1 - Treatment Period (Visit day ± days window)**														**Year 2 - Follow-up (Visit day ± window)**				**Unscheduled visit**	**Early Withdrawal**	
	Screen	D1	D8 ±3	D22 ±3	W4 ± 3	W8 ± 7	W12 ±7	W16 ± 7	W20 ±7	W24 ± 7	W28 ± 7	W36 ± 7	W44 ± 7	W52 ± 7	M15 ± 14	M18 ± 14	M21 ± 14	M24 ± 14		End of Therapy visit	8 week post last dose
Eligibility review	•																				
Informed Consent	•																				
Training on use of prefilled syringe		•																			
Pick up supply of Belimumab / placebo[1]		•			•	•	•	•	•	•	•	•	•								
Rituximab (intravenous infusion)[2]			•	•																	
Side effects review[3]	•	•	•	•	•	•	•	•	•	•	•	•	•	•	•	•	•	•	•	•	•
Medication check	•	•	•	•	•	•	•	•	•	•	•	•	•	•	•	•	•	•	•	•	•
Mental and physical health questionnaires	•	•	•	•	•	•	•	•	•	•	•	•	•	•	•	•	•	•	•	•	•
Physical examination	•	•	•	•	•	•	•	•	•	•	•	•	•	•	•	•	•	•	•	•	•
Routine blood tests and urine tests	•	•			•	•	•	•	•	•	•	•	•	•	•	•	•	•	•	•	•
Blood pregnancy test / menopause test[4]	•																				
Research blood tests		•	•		•	•	•	•	•	•	•	•	•	•	•	•	•	•	•	•	
Urine pregnancy test (WOCBP only)[4]		•			•	•	•	•	•	•	•	•	•	•	•	•					
Research urine tests		•			•	•	•	•	•	•	•	•	•	•	•	•	•	•	•	•	
Groin lymph gland and nose biopsy[5]		•					•														
Nose swab (optional)		•					•														

1. First belimumab/ placebo given in clinic under supervision followed by 3 hours observation. Thereafter, you will self-administer at home on weekly basis. Patients will be provided with sufficient doses of belimumab/ placebo to cover a 4 week period of weekly injections.
2. Observation is required for 1 hour post rituximab.
3. In-between visits from Month 6-12 (Weeks 32, 40, 48), the study team will telephone the participant once to check for any side effects
4. WOCBP: women of child bearing potential (i.e. fertile women pre-menopause). Home pregnancy test is required in-between visits from Month 6-12 (on weeks 32, 40 and 48) with telephone follow-up. A home pregnancy test will need to be taken at 16 weeks after the last dose of belimumab.

5. If biopsies have been consented to and if the biopsy is not performed on a scheduled trial visit day, then an extra research blood sample will be taken to allow comparison between the tissue and blood on that day.

Further details regarding the trial assessments are provided below. Please ask your doctor if you want more information.

- Medication check: You will be asked about medicines you are currently taking
- Physical examination: You will receive a complete physical examination including weight and vital signs (e.g. blood pressure measurements).
- Side effects review: You will be asked about any side effects that may be related to the trial medicines.
- Blood: will be taken from a vein for laboratory tests to help monitor your health and assess the safety and effectiveness of the treatments.

  The approximate total blood volume taken is as follows:
    - At each trial visit, approximately 20 mL up to 100 mL (approximately 5-15 teaspoons), depending on the assessments being made at each visit day. No more than 500 mL (approximately 2-3 cups) will be taken over any 6 month period.
    - If you remain in the trial up to the maximum period of 104 weeks, up to 900 mL of blood will have been drawn. For comparison, a person donating blood to the national blood bank would usually give up to 568 mL in one go. The 900 mL for the COMBIVAS trial will be spread over the 2 years.

  The blood samples will be used to check that it is safe for you to continue in the trial, to measure special markers to monitor your vasculitis disease activity and to measure any effects on your immune system. You will also be tested for hepatitis B, hepatitis C (viruses that cause inflammation of the liver), human immunodeficiency virus (HIV) antibodies, and evidence of previous or current tuberculosis (TB) infection. This is usual practice for any patient starting a new 'biologic' therapy like belimumab or rituximab. The tests may also include looking at changes in ribonucleic acid (RNA), which makes proteins in your body.

- Pregnancy test: If you are a woman and can have children, urine pregnancy tests will be carried out at regular intervals throughout the trial to ensure your safety. You will carry out some of these tests at home.
- You will give urine samples to test for vasculitis activity or damage within the kidneys.
- Questionnaires: You will be asked to complete several questionnaires to help assess your general health, your disease symptoms, your neurological health and your risk for suicide.

Nasal (inside the nose) biopsy

Vasculitis commonly causes inflammation of the inside of the nose and sinuses. Small nasal biopsies can be performed under local anaesthetic (injection to numb that part of the body) and may be useful in assessing the number of certain cell types in your immune system, disease activity and response to treatment at the tissue level.

The nasal biopsies will be performed under local anaesthetic by an Ear Nose & Throat consultant in the outpatients' clinic. The inside of your nose will be numbed with a local anaesthetic spray as well as an anaesthetic-soaked gauze that will be put into your nostrils for 10 minutes before the procedure. This will ensure adequate numbing of the inside of your nose and therefore the procedure should not be painful. We will take up to 2 small samples of tissue from the back of the nose. The procedure should take no longer than 20 minutes.

Blood tests will be taken before the procedure to ensure it is safe for you to have the biopsy. An extra research blood sample may also be taken if the biopsy is not done on a scheduled trial visit day.

What are the risks of a nasal biopsy?
Nasal biopsies are routine procedures with a very low complication rate. The most common risk of having a nasal biopsy is minor bleeding and infection. Bleeding can be easily treated using packing gauze or cauterising techniques (matchstick-like implement applied to small blood vessels to stop bleeding). Risk of infection is extremely low as we use sterile equipment and will give you some topical antibiotic cream to put in the nose after the procedure.

The most common risk of local anaesthetic is minor discomfort when injecting the inside of the nose. As with any medication, there is a small risk of an allergic response to the anaesthetic. Other possible side effects include dizziness, nausea, vomiting, accelerated heart rate, slow heart rate and prolonged numbness.

Lymph node biopsy
Lymph glands are important structures that maintain the health of the immune system. If you are exposed to an infection, the white blood cells and proteins that fight the infection are made within the lymph glands. In vasculitis the white blood cells and proteins that damage blood vessels are also thought to be made in the lymph glands. Therefore, studying the white blood cells in lymph glands can provide us with valuable information, which could help us to understand vasculitis better, predict response to treatment and risk of relapse. In order to assess the white blood cells in the lymph glands, a biopsy of a lymph gland is required.

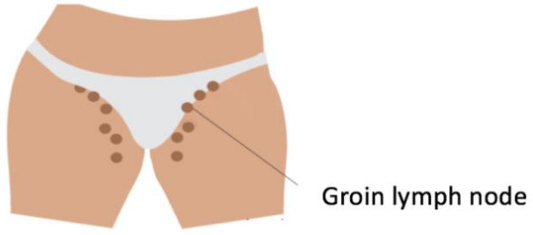

Lymph glands in the groin are just under the skin surface and are easily accessible. An ultrasound machine (jelly scan) is used to see the lymph glands in the groin easily. Using some jelly on your skin and a probe, the doctor can see the lymph glands on a screen. An injection of local anaesthetic is given under the skin to prevent pain. Two different techniques are used to obtain the biopsy sample. First, a small needle is used to suck out the cells from the lymph node. This is called fine needle aspiration (FNA). Following this a small (half a centimetre) cut is made in the skin. Through this small cut a larger needle is inserted to obtain another sample called a core needle biopsy. Wherever possible lymph node and nasal biopsies will be performed on the same day, so that the samples can be compared.

What are the risks of a lymph node biopsy?
The procedure is usually very well tolerated and the risk of major complications is very small. The main complication is minor pain and bruising. In a previous trial of lymph node biopsies, a short period of pain in the groin region was reported in up to 7% of participants and persistent pain for up to 2 months was reported in approximately 1%. There is a small risk of bleeding at the biopsy site, but applying pressure on the site immediately after the procedure and

applying a suitable wound dressing reduces this. There are no known long-term complications from this procedure. The risk of infection is extremely small.

Nose swab (for Cambridge and London participants only)
If you agree to undergo a nasal biopsy, and you are a participant in Cambridge or London, we will also ask you to have a nasal swab. During the process of nasal biopsy, it is usual practice to insert anaesthetic swabs in the nose during an anaesthetic procedure, therefore taking a swab during the procedure will cause no additional time or discomfort compared to performing the biopsy procedure alone. The nasal swab is an optional procedure.

The purpose of performing the nasal swab is to understand the effect of local infection on inflammatory cells in addition to the effects of trial medications.

The groin lymph node and nasal tissue biopsy procedures and the nasal swab are all optional. If you have agreed to undergo these procedures they will take place before starting the trial treatment and after 12 weeks of treatment. Your trial doctor will explain the procedures to you.

What is the "Genetic Research" part of this trial and why is it being done?
The purpose of the genetics part of this trial is to see why different people may react differently to medicines. We get our genes (DNA) from our parents, and different genes may affect who gets ANCA vasculitis and related conditions or how a body reacts to a drug.

Scientists look for differences in people's genes (DNA) that might explain this. Genetic research may include the study of certain genes or all your genes also called your whole genome. This may include genes involved in the way the drug works (both good and bad) or how the body deals with the drug. It may also include genes linked to the immune system, ANCA vasculitis or related conditions and so linked to drug response.

In COMBIVAS, we will collect data on RNA (similar to DNA) from your blood and tissue samples to help us understand more about the condition and how the trial treatments work.

How will being part of the COMBIVAS trial affect my lifestyle?
Participation in this trial should not affect your lifestyle. The frequency of the trial visits is greater than is usually needed for the average vasculitis patient, so you will be coming to the outpatient's clinic more often than you would if you don't join the trial. Other than this, we encourage you to live your normal life.

You will need to keep the belimumab (or placebo belimumab) in the fridge. You can still go away, (for example, on holiday), but you will need to check there is fridge in a safe place to store the trial drug. It is also important you commit to attending all of the trial visits and complete your patient diaries.

You must not donate blood for 16 weeks after last treatment/end of trial.

Pregnancy
Because the drugs being tested in this trial may affect an unborn baby you should not take part in this trial if:

- You are pregnant or planning to become pregnant.

- You are currently breastfeeding.

If you are a woman able to become pregnant, you must agree to avoid pregnancy by using a medically accepted method of contraception during your participation in this trial. Your doctor will tell you about the choices of contraception that you can use in this trial. Contraception and monthly pregnancy tests are required from the start of the trial until at least 4 months after the last dose of belimumab/placebo or 12 months after the last dose of rituximab, whichever is the latest. Some methods of contraception may not be approved for use during this trial.

If you become pregnant during this trial, you should contact the trial doctor right away using the details at the end of this information sheet. You will need to stop taking your trial drug. With your consent, you may be asked questions later about the pregnancy and the baby.

General advice

You will be given an emergency contact card with details of the trial you are in. You should carry this with you at all times and show this card to the medical staff if you need emergency care during the trial.

You should tell the trial team if you feel unwell or different in any way. If you have any major concerns or are feeling very unwell please contact your trial doctor immediately using the contact numbers at the end of this information sheet.

You should discuss your participation in this trial with any insurance provider you have (e.g. travel insurance, protection insurance, life insurance, income protection, critical illness cover and private medical insurance) and seek advice if necessary, as failure to notify them may affect or invalidate your cover.

What Medicines should I not take?

You should not take the following medicines during the trial:

- Any medicines that are part of other clinical trials
- Any immunosuppressive therapies (e.g. methotrexate, mycophenolate or azathioprine).
- Live vaccines
  - Vaccines help your body fight infections. If you get a vaccine while you are taking belimumab or rituximab, the vaccine might not work as well. Also, some types of vaccines may not be safe for you to get while you are taking belimumab or rituximab. Your trial doctor will review your vaccination history and any necessary vaccinations will be given prior to trial start. You will not be able to receive a live vaccine such as BCG (for tuberculosis), oral polio, measles, rotavirus and yellow fever vaccinations while taking part in the trial.

Talk with your trial doctor before you start any new medications or if you are unsure regarding whether you should be taking existing medications. In case of emergency, please do not delay taking necessary prescribed medication in order to talk to your trial doctor.

7. What are the side effects of the drug being tested?

You may have side effects while on this trial that may be related to rituximab or belimumab or a combination of both. Side effects may be mild or severe. We may give you medicine(s) to help lessen any side effects. Some side effects may go away as soon as you stop taking the

trial drug. In some cases, side effects can be serious, lasting or may never go away, or could rarely be fatal. If you are at all worried about your symptoms, then you can contact the trial doctor at any time (see contact name and number at the end of this leaflet). You may need to stop taking the trial drug after talking with the trial doctor.

Belimumab: possible side effects

Like all medicines, this medicine can cause side effects, although not everybody gets them.

Belimumab has mostly been studied in patients with systemic lupus erythematosus (also called "lupus" or "SLE"). In patients with diseases other than lupus, there is very little or nothing known about the side effects of belimumab. Safety information from lupus patients is given here.

Infections: Because of the type of medicine it is, belimumab may make it easier for you to get an infection. These infections can be serious and occasionally fatal. Up to 1 in 10 people may experience a serious infection. Other medicines that you may be receiving for your ANCA vasculitis can also increase your risk for infection. Tell your trial doctor or nurse right away if you have any of the following symptoms which may be a sign of an infection: fever, chills, pain or burning during urination, urinating often, bloody diarrhoea, or coughing up mucus. If you have any infection that keeps coming back or is hard to get rid of, you should not be treated with belimumab.

Progressive Multifocal Leukoencephalopathy (PML): PML is a rare but serious and life threatening brain condition. Your chance of getting PML may be higher if you are treated with medicines that weaken your immune system, including belimumab and rituximab. Tell your doctor immediately if you have memory loss, trouble thinking, difficulty with walking or talking, loss of vision, or similar problems.

Allergic (hypersensitivity) and infusion reactions: The most common side effects that happen during or soon after a belimumab dose are nausea, headache, rash, itching and fever. These side effects are usually not severe. Some patients (up to 1 out of 100) have more severe side effects. These may happen because of an allergic reaction. Patients who have had allergic reactions before may be more likely to have allergic reactions to belimumab. These reactions were observed in patients receiving belimumab by intravenous infusion and generally happened on the day of or day after the first or second belimumab dose was given. In this trial, belimumab will not be administered as an intravenous infusion but as an injection (under the skin). In a trial in lupus patients using the injection form of belimumab (under the skin), skin reactions in the area of injection occurred in less than 1 in 10 people, and were mild or moderate in severity. No serious reactions occurred.

Hypersensitivity reactions can be severe and in rare circumstances can be fatal. Symptoms of a severe reaction may include swelling of the face, mouth, throat, lips, or tongue; wheezing, trouble breathing or shortness of breath; rash, which may be raised and itchy (hives); dizziness or fainting; high or low blood pressure; headache; or nausea. Seek medical attention right away if you get any of these symptoms. Your trial doctor or trial nurse will watch you closely during and for 3 hours after your first injection of trial drug for signs of reaction. In addition, if you miss 4 or more weekly injections of belimumab in a row the trial doctor will observe you for injection reactions for 3 hours in the clinic after your next dose.

Delayed-type allergic reactions can occur with belimumab. These types of reactions generally

occur 5-10 days after a dose of medication (but can occur before or after that time) and include a combination of symptoms such as rash, nausea, fatigue, muscle aches, headache, and/or facial swelling. If you experience these symptoms, particularly if you experience a combination of such symptoms contact your trial doctor or nurse.

Cancer: Belimumab may reduce the activity of your immune system. Medicines that affect the immune system may increase your risk of certain cancers. So far, patients who have had belimumab have not developed cancer more often than patients who have not had belimumab.

Mental health problems and suicide: Mental health problems, such as depression, trouble sleeping, and anxiety, are common in patients with lupus and were reported in patients receiving belimumab. Symptoms of mental health problems can include thoughts of suicide or dying, attempt to commit suicide, trouble sleeping (insomnia), new or worse anxiety or depression, acting on dangerous impulses, other unusual changes in your behaviour or mood, or thoughts of hurting yourself or others. Tell your trial doctor or trial nurse right away if you have any of these symptoms. Tell your doctor even if these problems are not bothering you a lot right now. Also, tell your trial doctor if you are having any mood problems, are not acting or feeling like yourself, or are having behaviour problems. If your family or friends have told you that they think you have these problems, tell your doctor, even if you don't agree. You will be asked questions about your mood and any thoughts of harming yourself that you may have during the trial.

Very common side effects (may affect more than 1 in 10 people):

- Nausea
- Diarrhoea
- Infections, such as chest, bladder, nose, throat, or stomach infections
- Fever

Common side effects (may affect up to 1 in 10 people):

- Stomach virus, sore throat, runny nose
- Sleeplessness, depression
- Pain in hands or feet
- Migraine
- Low white blood cell count

Uncommon side effects (may affect up to 1 in 100 people)

- Severe allergic reactions, sometimes with swelling of face or mouth causing difficulty in breathing
- Swelling of the face, lips and tongue
- Wheezing, difficulty in breathing or shortness of breath
- Rash
- Itchy raised bumps or hives

Rituximab: possible side effects

Rituximab is considered as 'standard of care' for the treatment of vasculitis. This means that you are likely to receive rituximab as part of routine practice. Side effects of rituximab are therefore not specific to the trial.

Most side effects are mild to moderate but some may be serious and require treatment. Rarely, some of these reactions have been fatal.

Infections: Serious, including fatal, bacterial, fungal and new or reactivated viral infections can occur during and following rituximab therapy. You might get infections more easily during your treatment. Tell your doctor or nurse right away if you have any of the following symptoms which may be a sign of an infection: fever, chills, pain or burning with urination, urinating often, bloody diarrhoea, or coughing up mucus.

Progressive Multifocal Leukoencephalopathy (PML): As with belimumab, there is an increased risk of PML in patients receiving rituximab, although this is very rare.

Infusion Reactions: During or within the first 2 hours of the first rituximab infusion you may develop fever, chills and shivering. Less frequently, some patients may experience pain at the infusion site, blisters, itching, sickness, tiredness, headache, breathing difficulties, tongue or throat swelling, itchy or runny nose, vomiting, flushing or palpitations, heart attack or low number of platelets. If you have heart disease or angina, these reactions might get worse. Tell the person giving you the infusion immediately if you develop any of these symptoms, as the infusion may need to be slowed down or stopped. When these symptoms go away, or improve, the infusion can be continued. These reactions are less likely to happen after the second infusion. Your trial doctor may decide to stop your rituximab treatment if these reactions are serious.

Skin Reactions: Very rarely, severe blistering skin conditions that can be life-threatening may occur. Redness, often associated with blisters, may appear on the skin or on mucous membranes, such as inside the mouth, the genital areas or the eyelids, and fever may be present. Tell your trial doctor immediately if you experience any of these symptoms.

Very common side effects (may affect more than 1 in 10 people):

- Bacterial or viral infections, bronchitis
- Low number of white blood cells, with or without fever or blood cells called “platelets”
- Nausea
- Chills, headache
- Lower immunity – because of lower levels of anti-bodies called “immunoglobulins” in the blood which help protect against infection

Common side effects (may affect up to 1 in 10 people):

- Infections of the blood (sepsis), pneumonia, shingles, cold, bronchial tube infections, fungal infections, infections of unknown origin, sinus inflammation, hepatitis B
- Low number of red blood cells (anaemia), low number of all blood cells
- Allergic reactions (hypersensitivity)
- Unusual feelings of the skin – such as numbness, tingling, pricking, burning, a creeping skin feeling, reduced sense of touch
- Feeling restless, problems falling asleep
- Becoming very red in the face and other areas of the skin as a consequence of dilatation of the blood vessels
- Feeling dizzy or anxious

- Producing more tears, tear duct problems, inflamed eye (conjunctivitis)
- Ringing sound in the ears, ear pain
- Heart problems – such as heart attack, uneven or fast heart rate
- High or low blood pressure (low blood pressure especially when standing upright)
- Tightening of the muscles in the airways which causes wheezing (bronchospasm), inflammation, irritation in the lungs, throat or sinuses, being short of breath, runny nose
- Being sick (vomiting), diarrhoea, pain in the stomach, irritation or ulcers in the throat and mouth, problems swallowing, constipation, indigestion
- Eating disorders, not eating enough, leading to weight loss
- Hives, increased sweating, night sweats
- Muscle problems – such as tight muscles, joint or muscle pain, back and neck pain
- General discomfort or feeling uneasy or tired, shaking, signs of flu

Uncommon side effects (may affect up to 1 in 100 people):

- Blood clotting problems, decrease of red blood cell production and increase of red blood cell destruction (aplastic haemolytic anaemia), swollen or enlarged lymph nodes
- Low mood and loss of interest or enjoyment in doing things, feeling nervous
- Taste problems – such as changes in the way things taste
- Heart problems – such as reduced heart rate or chest pain (angina)
- Swelling of the stomach.

Very rare side effects (may affect up to 1 in 10,000 people):

- Nerve damage in arms and legs, paralysed face
- Heart failure
- Inflammation of blood vessels including those leading to skin symptoms
- Respiratory failure
- Damage to the intestinal wall (perforation)
- Severe skin problems causing blisters that can be life-threatening. Redness, often associated with blisters, may appear on the skin or on mucous membranes, such as inside the mouth, the genital areas or the eyelids, and fever may be present.
- Severe vision loss

Combination of belimumab and rituximab

In a recent vasculitis trial run by GSK, belimumab was compared to placebo. In this trial patients received either cyclophosphamide or rituximab 4 months before starting either belimumab or placebo. The patients who received rituximab followed by belimumab may have had infections at a greater frequency than those who received rituximab followed by placebo. The infections were mainly non-serious. There was a trend towards improved vasculitis disease control in the patients who received rituximab followed by belimumab compared to patients who received rituximab followed by placebo. However, because only a small number of patients received rituximab in this trial, we cannot make firm conclusions about the safety and effectiveness of rituximab followed by belimumab and more research is required.

Both belimumab and rituximab may increase your risk of infection and/or suffering an adverse allergic reaction when the drug is given to you. It is possible that this risk may be increased when both belimumab and rituximab are administered at the same time. Your trial doctor can explain to you the steps that will be taken to reduce this risk.

In addition, it is also possible that the risks of cancer and psychiatric side effects (feeling anxious or depressed) could be increased further when both belimumab and rituximab are administered at the same time.

Other possible side effects:
There may be other side effects that may happen that are not known now. For example, all drugs can cause an allergic reaction in some patients. Certain problems can become worse if not treated quickly. Call the trial doctor right away if:

- You feel very tired or faint
- You feel pain or sick in your stomach and you do not want to eat
- You bruise easily or develop itching
- You have yellow eyes or skin, or dark urine
- You become confused.

If you experience certain other serious problems (such as an allergic reaction, swelling, difficulty breathing, a bad skin rash, liver or kidney damage, or changes in your heart rhythm), you may be asked to return to the clinic for more tests. This may include more blood tests. The trial doctor will explain these tests to you if they are needed. You may also need to stop taking the belimumab after talking with the trial doctor.

8. What are the possible disadvantages and risks of taking part?

In addition to side effects related to medications, when giving blood you may feel faint, or experience mild pain, bruising, irritation or redness at the site. In rare cases, you may get an infection.

During the trial, (at the beginning and 3 months later), you will receive a local anaesthetic and undergo two minor surgical procedures to take samples of tissue from the lymph nodes in your groin, and from your nasal passage. After the procedure your nose and groin may be sensitive for a short period of time. Some bleeding may occur and as with all surgical procedures, there is a risk of infection.

9 visits to the clinic are required over the first 12 month period (every 4 weeks for 6 months, then every 8 weeks for 6 months). This is more frequent than is usually required for the standard treatment of vasculitis.

When deciding whether to take part in this trial, consider how the tests listed above and the visit schedule will affect your work and family schedules. Consider if you need transportation to get to the clinic. You may find that these tests and visits are inconvenient and require special effort. In addition, some tests may be uncomfortable. Ask us if you have any questions about the tests and procedures for the trial.

You will need to allow enough time to receive phone calls from trial staff. You will need to allow enough time each week to complete your self-injection diary.

9. What are the possible benefits of taking part?

There is no guarantee that you will benefit from taking part in this trial. You may experience relief in your symptoms or an improvement in your disease. However, information collected as part of your participation in this trial may benefit patients with ANCA vasculitis in the future.

10. What are the alternatives for treatment?

Your trial doctor can tell you about other treatment choices for your ANCA vasculitis. You may choose to:

- Continue to get regular care from your doctor
- Take part in another trial
- Get no treatment at this time.

Talk with your trial doctor about your choices.

11. What happens when the trial stops?

As belimumab is currently not a licenced treatment for ANCA associated vasculitis, you will not receive any more belimumab when the trial stops. Your standard vasculitis treatment will be continued as required, according to your local hospital practice.

After the whole trial is finished, we can tell you which drug you were given if you wish to know. The results of the trial will also be available. Your study doctor can provide these if requested.

12. Expenses & Payment?

You will not receive any payment for participating in this trial; however, we can reimburse any reasonable travel expenses and parking costs incurred for making extra visits to the hospital for the trial which are in addition to your regular clinical visits, upon production of receipts.

Section 2: Trial Conduct

13. What if new information becomes available?

Sometimes during the course of a trial, new information becomes available which might affect your decision to continue participating in this trial. Your trial doctor will contact you to discuss the new information and whether you wish to continue participating in the trial. If you still wish to continue on the trial, you will be asked to sign a new Informed Consent Form.

The trial sponsor, the regulatory authority or the trial doctor may decide to stop the trial at any time. If that happens we will tell you why the trial has been stopped and arrange for appropriate care and treatment for you.

14. What if I decide I no longer wish to participate in the trial?

You are free to stop taking part in the trial at any time without giving a reason and without affecting your future care or medical treatment. If you decide not to participate any further, you will no longer receive the trial treatment. No further tests will be performed on you and no further research samples will be collected. Any data already collected or results from tests already performed on you or your samples will continue to be used in the trial analysis unless you request that your samples are destroyed.

The trial doctor may also choose to withdraw you from the trial if they feel it is in your best interests or if you have been unable to comply with the requirements of the trial. Reasons for trial withdrawal could include:

- You have experienced a serious side effect
- You are unable to complete the visits, medication or trial documentation as required
- You become pregnant or plan to become pregnant

- The trial doctor feels you no longer appear to benefit from the treatment.

If you have experienced any serious side effects during the course of the trial which require you to withdraw from the trial, your trial doctor will follow-up with you regarding your progress until the side effect has stabilised or resolved.

If you decide to leave the trial and withdraw your consent, with your permission, we would still like to contact you to learn about your well-being. This will be done by phone calls and may include attending a final trial visit for assessments to ensure your safety.

Any leftover trial drug will need to be returned to the trial team.

Can I stop taking the medications but stay in the trial?
If you decide to stop taking trial medication, you and the trial doctor will discuss the best way to do this. We will ask you to return any leftover trial drug to the trial team.

It is possible to stop taking trial drug and stay in the trial. If you do not want to take trial drug for the rest of the trial, your health information is still very important information for the trial. You will be asked to return to your trial doctor's clinic for all appropriate trial visits and assessments and the trial doctor or nurse will contact you on the telephone to monitor your health but you will not need to continue taking trial treatment. If you are unable to continue with all the remaining trial visits and assessments, your trial doctor will arrange for you to attend a final visit for assessments before you leave the trial.

All the data and samples collected before you left the trial will still be used for the trial, unless otherwise specified.

15. What if there is a problem?

Any complaint about the way you have been dealt with during the trial or any possible harm you might suffer will be addressed. If you have any concerns about any aspect of this trial, you should speak to your trial doctor who will do their best to answer your questions.

In the event that something does go wrong and you are harmed by taking part in the research and this is due to someone's negligence then you may have grounds for a legal action for compensation against Cambridge University Hospitals NHS Foundation Trust or the University of Cambridge. The University has obtained insurance which provides no-fault compensation i.e. for non-negligent harm, you may be entitled to make a claim for this.

If you wish to complain or have any concerns about any aspect of the way you have been approached or treated during this trial, you can do this through the NHS complaints procedure. In the first instance it may be helpful to contact the Patient Advice and Liaison Service (PALS) at your hospital (see section 21 for contact details).

16. Will my taking part in this trial be kept confidential?

Cambridge University Hospitals NHS Foundation Trust (CUHFT) and the University of Cambridge are the sponsors for this trial based in the United Kingdom. They will be using information from you and your medical records in order to undertake this trial and will act as the data controller for this trial. This means that they are responsible for looking after your information and using it properly.

Your rights to access, change or move your information are limited, as the Sponsor organisations need to manage your information in specific ways in order for the research to be reliable and accurate. If you withdraw from the trial, we will keep the information about you that we have already obtained. To safeguard your rights, we will use the minimum personally-identifiable information possible.

You can find out more about how the Sponsors use your information by visiting the links below:

- For Cambridge University Hospitals NHS Foundation Trust, please visit: https://www.cuh.nhs.uk/corporate-information/about-us/our-responsibilities/looking-after-your-information, or email the Data Protection Officer at: gdpr.enquiries@addenbrookes.nhs.uk

- For University of Cambridge, please visit:

https://www.medschl.cam.ac.uk/research/information-governance/, or email the Information Governance team at: researchgovernance@medschl.cam.ac.uk

Cambridge University Hospitals NHS FT will collect your name, NHS number, date of birth and contact details to contact you about this trial, and make sure that relevant information about the trial is recorded for your care, and to oversee the quality of the trial. Individuals from the Sponsors and regulatory organisations may look at your medical and research records to check the accuracy of this trial. Cambridge University Hospitals NHS FT will pass these details to the Sponsors along with the information collected from you and/or your medical records. The only people in the Sponsor organisations who will have access to information that identifies you will be people who need to contact you in relation to this trial and to audit the data collection process. Cambridge University Hospitals NHS FT will keep identifiable information about you from this trial for 15 years after the trial has finished.

Once the trial has come to an end, we will be required to archive trial documentation, which may contain some personal data (e.g. your name will be recorded as part of completing the consent form). This trial documentation will be retained for at least 15 years after the trial has ended and will be securely archived according to Sponsor policy.

Your information could be used for research in any aspect of health or care, and could be combined with information about you from other sources held by researchers, the NHS or government. Where this information could identify you, the information will be held securely with strict arrangements about who can access the information. The information will only be used for the purpose of health and care research. It will not be used to make decisions about future services available to you, such as insurance. Where there is a risk that you can be identified your data will only be used in research that has been independently reviewed by an ethics committee.

We will need to inform your GP of your participation in this trial so that any medical decisions made by your GP account for any treatment you are receiving as part of this trial.

All information will be treated in the strictest confidence during the review process.

17. What will happen to my samples?

If you take part in this trial, you will be asked to give blood, urine, lymph node biopsy and nasal biopsy samples. If you give permission, some tissue from other biopsies you may undergo as part of your routine clinical care (e.g. kidney, lung), may be analysed as well. These samples will be used for various tests to assess safety, the effects of the trial treatments on your vasculitis, and the effects of the trial treatments on the underlying disease processes which cause your vasculitis. Similar to the information collected in the trial, your samples may also be used by the trial team or shared by the trial team with other companies or universities to better understand vasculitis, other diseases or conditions, or to further develop the trial drug or other drugs.

Your blood, urine, lymph node and nasal and other tissue samples will be given the same code as your other trial information and kept in locked storage within University of Cambridge for up to 1 year after the end of the trial. After this one year period, your samples will either:

1. Be destroyed
2. Be transferred to an approved licenced storage facility (e.g., tissue bank)
3. Be transferred for use in a separate ethically-approved research study

Anyone who works with your samples will hold the information and results in confidence.

Some of the specialist tests performed on your blood will be performed in GSK laboratories in the USA and therefore require transfer outside the EU. You will not be able to be identified from these samples.

18. What will happen to the results of the trial?

The results of the trial will be anonymous and you will not be able to be identified from any of the data produced. When the results of this trial are available they may be published in peer-reviewed medical journals and used for medical presentations and conferences. Your anonymised genetic data may also be published as a supplement alongside the publication. At no time, will any personal / confidential information about you, which might allow you to be individually recognised, be released in any publicly available or published material. The trial will be registered on a publically accessible database, for example the EU Clinical Trials Register and/or ClinicalTrials.gov.

GSK will receive anonymised trial data at the end of the trial. This will include data about you, your vasculitis and how your assessments (blood tests, urine tests, symptoms, questionnaires etc) have changed throughout the trial. They will also collect safety data. As the manufacturer of belimumab, GSK are required to keep track of all the data produced from trials that use their products. They want to collect information on how well the drug works in different conditions and look for trends in side effects that may not be apparent from smaller single studies. Ultimately, they are looking to see if belimumab works and is safe for patients with vasculitis. If they can show this, the data may be used for future drug licence applications to use belimumab in patients with vasculitis.

Fully anonymised data collected during the trial will be stored in a password protected database for up to 25 years. The purpose of the retention of data is to allow comparison with future studies, especially those involving your stored samples that may have been transferred for the use in other ethically-approved research studies.

The trial team may:

- Keep your coded information electronically, and analyse it by computer to find out what the trial is telling us. This work may be done by the University of Cambridge or GSK.

- share the information with regulatory agencies that approve new medicines,

- share the information with people who check that the trial is done properly (like the ethics committee or review boards),

- combine the information with results from other studies to learn more about the medicine and other medicines, and ANCA vasculitis and other diseases and conditions. This may help us to assess the risks and benefits of GSK (or other) medicines, or to improve disease understanding.

If you would like to obtain a copy of the published results, please contact your trial doctor directly who will be able to arrange this for you.

19. Who is organising (sponsoring) and funding the trial?

This trial is sponsored by Cambridge University Hospitals NHS Foundation Trust and the University of Cambridge.

The research is being funded by the Medical Research Council. GSK is providing the belimumab trial drug and its matching placebo, trial expertise, some specialist laboratory analyses and additional funding for the central running of the trial.

20. Who has reviewed this trial?

All research within the NHS is reviewed by an independent group of people called a Research Ethics Committee, to protect your interests. This trial has been reviewed and given favourable opinion by the Cambridgeshire and Hertfordshire research Ethics Committee. The Medicines and Healthcare Products Regulatory Agency (MHRA) who are responsible for regulating medicines in the UK have also reviewed this trial.

21. Further information and contact details

You can talk with the trial doctor about any questions or concerns you have about the trial, or if you think you have been hurt from taking part in the trial or if you have any questions about side effects. Please call:-

Dr. Rachel Jones ____________ Telephone no. 01223 254637________ at any time.
Dr. Mark McClure____________ Telephone no. 01223 256730__________ _at any time.
Nurse Karen Dahlsveen ________ Telephone no. 01223 336819_ ____Tues, Weds and Fri.
Nurse Valentina Ruffolo________ Telephone no. 01223 256729___________ at any time.

Your hospital's Patient Advice and Liaison Service (PALS) contact details are:
Email: pals@addenbrookes.nhs.uk Telephone no. 01223 216 756

In the event of an emergency please contact:
If you require advice out of normal office hours, please call Addenbrooke's switchboard on 01223 245151 and ask to speak to the on call renal registrar.

INFORMED CONSENT FORM

Trial Title: COMBIVAS: A randomised, double blind, controlled mechanistic trial of rituximab and belimumab combination therapy in PR3 ANCA-associated vasculitis

Principal Investigator: **Trial ID:** _________

If you agree with each sentence below, please initial the box **INITIALS**

1	I have read and understood the Participant Information Sheet version 2.0, dated 4th July 2019 for the above trial and I confirm that the trial procedures and information have been explained to me. I have had the opportunity to ask questions and I am satisfied with the answers and explanations provided.	
2	I understand that my participation in this trial is voluntary and that I am free to withdraw at any time, without giving a reason and without my medical care or legal rights being affected.	
3	I understand that my date of birth (personal information) will be collected on the trial documentation and stored in the trial database, but that this information will be kept in the strictest confidence and none of my personal data will be published.	
4	I understand that sections of my medical notes or information related directly to my participation in this trial may be looked at by responsible individuals from the sponsor, regulatory authorities and research personnel where it is relevant to my taking part in research. I give permission for these individuals to have access to my records.	
5	I understand that my GP will be informed of my participation in the COMBIVAS trial and will be sent details of the trial.	
6	I have read and understood the compensation arrangements for this trial as specified in section 12 of the Participant Information Sheet.	
7	I understand that the doctors in charge of this trial may close the trial, or stop my participation in it at any time without my consent.	
8	I have read and understood my responsibilities for the trial including using appropriate contraception as listed in section 6.	
9	I know what will happen to my blood/urine/tissue samples and information collected for this trial and understand that my anonymised, coded information may be shared with other companies, organisations or universities to carry out future ethically approved research (as stated in section 17 and 18).	
10	I agree to allowing my samples to be sent outside of the UK for testing.	
11	I consent to genetic analysis being performed on my blood and tissue samples and I understand that my anonymised genetic data may be uploaded onto a publically accessible database as a supplement to the publication of this study.	
12	I have been given the names of trial staff who I can call.	
13	I freely agree to take part in this trial.	

**OPTIONAL**		**YES**	**NO**
14	I agree to allowing some tissue from any biopsy samples taken as part of my routine clinical care to be used for ethically approved research purposes, as part of this trial. I understand that samples and results will be sent securely within the trial team.		
15	I agree to undergo the nasal biopsy procedures.<br>I understand I can withdraw consent at any time.		
16	I agree to have a nasal swab taken at the same time as the biopsy (Cambridge and London participants only).<br>I understand I can withdraw consent at any time.		
17	I agree to undergo the lymph node biopsy procedures.<br>I understand I can withdraw consent at any time.		
18	I agree to allow the use of my unused samples to be stored in a licenced storage facility (e.g. tissue bank) or be used for research purposes in other future ethically-approved studies.		

I agree to participate in this trial:

______________________________ ______________________________ ____________
Name of patient Signature Date

______________________________ ______________________________ ____________
Name of person taking consent Signature Date

Time of Consent (24hr clock) _______:_______

1 copy for the patient, 1 copy for the trial team, 1 copy to be retained in the hospital notes.
